# Supplementary material for: Narcolepsy risk loci outline role of T cell autoimmunity and infectious triggers in narcolepsy
Source: Nat Commun. 2023 May 15;14:2709. doi: 10.1038/s41467-023-36120-z (PMC10185546; doi:10.1038/s41467-023-36120-z)
Supplement: Supplementary file 1 — Supplementary Information [file 41467_2023_36120_MOESM1_ESM.pdf]

# Supplementary information for: "Narcolepsy risk loci outline role of T cell autoimmunity and infectious triggers in narcolepsy "

Ollila HM, Sharon E, Lin L, Sinnott-Armstrong N, Ambati A et al*

*Full list of authors appears in the main paper

**Contents**

1. Cohorts
    1.1. Japanese Narcolepsy cohort
    1.2. Chinese Narcolepsy cohort
    1.3. East Asian Stanford Narcolepsy cohort
    1.4. Stanford Narcolepsy samples with primary African Ancestry
    1.5. Stanford Narcolepsy AFFY 6.0/AFFY 500K legacy cohort
    1.6. Stanford and European Narcolepsy samples with primary European Ancestry
    1.7. Data from FinnGen
2. Main Analyses
    2.1. Analysis for P-value distribution
    2.2. Regional associations
    2.3. Vaccination-related narcolepsy
    2.4. Influenza infection and *IFNAR1*
    2.5. Functional analysis of T cell receptors
3. Bibliography

*The purpose of this document is to provide information on cohorts, methods and findings that are not included in the main paper.*

# 1. COHORTS

## 1.1 JAPANESE NARCOLEPSY COHORT

The Japanese cohort is a clinical cohort of 409 unrelated type 1 narcolepsy (NT1) patients and 1,562 unrelated Japanese controls (N total = 1,971) genotyped and analyzed in Japan. All individuals lived in mainland Japan. Clinical diagnosis for NT1 mandated: 1) clear-cut cataplexy and excessive daytime sleepiness, and 2) absence of any other sleep disorder. The study was approved by an ethics review committee and all individuals provided written informed consent. Study participants were genotyped with the Affymetrix 500k array. Variants and genotypes were filtered for missing data. Individuals with 1% or more missing genotypes were excluded. We also excluded variants with more than 1% missing genotypes. A total of 108 individuals (n=22 T1N cases and 86 controls) were removed because of Quality Control (QC) prior to imputation. 43,400

SNPs were excluded based on genotyping rate (1% or more missing genotypes). 503,239 variants and 1,863 subjects passed filters and QC. We computed relatedness using Plink (Chang et al., 2015) and there were no related individuals in the data set (Pi_hat score under 0.2). Genotypes were aligned into hg19 positions, pre-phased with SHAPEIT (Delaneau et al., 2011) and imputed to 1000 Genome reference V1 using IMPUTE2 (Howie et al., 2009). Variants with $r^2$ imputation quality < 0.7, minor allele frequency <0.01 and deviation from Hardy-Weinberg Equilibrium $p<10^{-6}$ were removed. We found no evidence of genomic inflation post association: lambda = 1.032. Association testing was run with SNPTEST v2.5 (Marchini et al., 2007) using a genotype threshold regression model and adjusting for population stratification using the first 10 principal components. Japanese-only analysis led to only two genome-wide significant loci post imputation: HLA DQB1*06:02 $p<10^{-200}$ and TRA rs1154155 $p<10^{-11}$.

## 1.2 CHINESE NARCOLEPSY COHORT

The Chinese Type 1 Narcolepsy cohort initially included 1,526 individuals with NT1 and 1,998 controls genotyped and analyzed in China. For inclusion, patients had to have excessive daytime sleepiness and either cataplexy and/or low cerebrospinal fluid (CSF) hypocretin-1 levels. For a subset of the sample, Genome Wide Association Study (GWAS) was performed on Affymetrix CHB1 array (N=1,189 NT1 cases and N=1,998 controls); for these genotyping and QC have been described previously (Han et al., 2013). Three hundred and thirty-seven additional individuals were genotyped using the Axiom Precision medicine PMRA array. In the PMRA array sample, 13 individuals were removed due to low genotyping rate (missingness per individual >1%), 59,589 variants were removed due to low genotyping rate (missingness per marker >1%) and 14,439 variants were removed due to Hardy Weinberg $p<10^{-6}$. Principal components were calculated using eigenvectors as implemented in PLINK v.190 (Chang et al., 2015). Ten individuals were removed as they did not cluster with the other samples, 6 cases from the PMRA Axiom platform, 4 cases from the CHB1 samples and 2 controls from CHB1 samples. PCs were then recomputed and visualized for matching. A final sample of 1,497 cases and 1,992 controls remained in the analysis. PMRA and CHB1 array samples were phased using SHAPEIT (Delaneau et al., 2011) and imputed using IMPUTE2 (Howie et al., 2009). Genotypes were merged post imputation. Variants with minor allele frequency 1%, Hardy-Weinberg Equilibrium $p<10^{-6}$ and imputation info/$r^2$ <0.7 were removed from the analysis. Association testing was run with SNPTEST v2.5 (Marchini et al., 2007) using a genotype thresholded regression model . To adjust for population stratification, analyses were adjusted for the 10 first principal components.

## 1.3 EAST ASIAN STANFORD NARCOLEPSY COHORT

The East Asian sample initially included 562 individuals with narcolepsy from China (N=320), Japan (N=1), Taiwan (N=11), and Korea (N=182), as well as individuals of Asian decent living outside Asia (N=48) genotyped at University of California, San Francisco (UCSF). To match these, 5,182 controls of Asian descent were obtained from the Genetic Epidemiology Research on Adult Health and Aging (GERA) Cohort also genotyped at UCSF (Banda et al., 2015). All participants provided written informed consent. Cases had a clinical diagnosis of NT1 and had cataplexy or low CSF hypocretin-1. They also had excessive daytime sleepiness and cataplexy or verified low hypocretin levels. Both NT1 cases and individuals without narcolepsy were genotyped at University of California, San Francisco. Study subjects were genotyped with the Affymetrix Axiom EAS array, CEL files called using AffyPipe and exported genotypes as plink ped/map files. After QC, 34 cases with NT1 were removed due to missing genotype data (> 1% missing genotypes per individual), 48,072 variants removed due to missing genotype data (>1% missing genotype data per variant).

7,797 variants were removed due to Hardy-Weinberg exact test deviations (P <$10^{-6}$) and 44,213 variants were removed because allele frequency was <1%. A total of 589,907 variants and 565 people passed filters and QC. Control genotypes were requested from dbGAP (phg000583.v2). Related individuals were excluded (N=345) using Plink Pi_hat score. Markers with Hardy-Weinberg P-value <$10^{-6}$(N = 18,306 markers), minor allele frequency < 1% (N = 30,831 markers), and genotyping rate < 1% (N = 82,284 markers) were removed. Individuals with missing genotypes over 1% (N = 205 controls) were removed. Final data set prior to imputation included 563,456 variants and 4,632 individuals. We calculated eigenvectors for genetic distance as implemented in PLINK v.190 (Chang et al., 2015), also examining self-reported ancestry and country of origin within Asians (Banda et al., 2015). Based on this, we identified 8 outlier individuals, which were excluded. In addition, we removed five related narcolepsy cases and 239 controls based on relatedness as calculated by PLINK 1.90 PI_HAT value (threshold Pi_Hat > 0.2). Total sample after QC was 543 cases and 4,943 controls. Markers were merged, lifted to hg19 positions and phased using SHAPEIT (Delaneau et al., 2011) and imputed using IMPUTE2 (Howie et al., 2009) and 1000 Genomes V3. For analysis, markers with info score less than 0.7, Hardy-Weinberg p-value <$10^{-6}$, and missingness over 1% were excluded. Association testing was run using SNPTEST v2.5 (Marchini et al., 2007) and a genotype thresholded regression model after adjusting for first 10 principal components.

## 1.4 STANFORD NARCOLEPSY COHORT WITH PRIMARY AFRICAN ANCESTRY

Clinical cases of NT1 (n=284 individuals) with excessive daytime sleepiness and clear cut cataplexy or verified hypocretin deficiency were included. Ethnically matched controls were obtained from the Genetic Epidemiology Research on Adult Health and Aging (GERA) Cohort (Banda et al., 2015). Cases and controls were genotyped with Axiom GW AFR genotyping chip at University of California San Francisco. We called CEL files using AffyPipe and exported genotypes as plink ped/map files. 23,3571 variants were removed due to missingness >1%. 16,210 variants were removed due to Hardy-Weinberg Equilibrium p <$10^{-6}$. 34 individuals were removed due to genotype missingness >1%. A total of 583,503 variants and 250 individuals passed filtering and QC. For controls, QC had already been performed using similarly stringent criteria. Specifically, the GERA Black cohort was downloaded from dbGAP (phg000583.v2). 187,622 out of 851,970 downloaded variants were removed due to missing genotype data (missingness per marker > 1%). 68,135 variants were removed due to Hardy-Weinberg exact test deviation (P < $10^{-6}$). 58,057 variants removed due to minor allele frequency < 1%. In addition, we removed 164 individuals due to relatedness and 132 individuals due to missing genotype data (missingness per individual > 1%). 538,156 variants and 3,521 people passed filters and QC; samples were then merged. A total of 416,323 variants and 3,771 individuals were kept in the analysis. SNPs were pruned and eigenvectors calculated using PLINK v.190 (Chang et al., 2015). Principle components reflecting population structure were then visualized and controls and cases that clustered together with Black ancestry were kept in the analysis (37 individuals with NT1 and 1,774 controls). The final numbers prior to imputation were 213 cases and 1,747 controls. Markers were merged, lifted to hg19 positions and phased using SHAPEIT (Delaneau et al., 2011) and additional markers imputed using IMPUTE2 (Howie et al., 2009) and the 1000 Genomes V3 reference. For analysis, markers with info score less than 0.7, Hardy-Weinberg p-value <$10^{-6}$ and missingness over 1% were excluded. Association testing was run with SNPTEST v2.5 (Marchini et al., 2007) using a genotype thresholded regression model, while adjusting for the first 10 principal components.

## 1.5 STANFORD AFFY 6.0/AFFY 500K LEGACY COHORT

This cohort is comprised of previously reported individuals with narcolepsy (N=625 with Affy6, and 182 individuals with Affymetrix 500k) and controls (N=915 with Affy6 and 167 with Affymetrix 500k, total N=1,889) from Hallmayer et al. (Hallmayer et al., 2009). All cases had cataplexy and, in all subjects, where hypocretin was measured (23% of sample), CSF hypocretin was low. Previously QCed genotypes from Affy 6.0 genotyping were used (Hallmayer et al., 2009). These genotypes were originally QCed for minor allele frequency (<0.05), Hardy-Weinberg equilibrium (p<0.01 in controls). Principal components were calculated as implemented in PLINK 1.90 and visualized. No outliers were detected. Altogether 549,596 markers passed quality control and were used for imputation. The variants were lifted to hg19 chromosome positions and phased using SHAPEIT (Delaneau et al., 2011) and imputed using IMPUTE2 (Howie et al., 2009) with the 1000 Genomes V1 as reference. For analysis, markers with info score less than 0.7, Hardy-Weinberg p-value $<10^{-6}$ or missingness over 1% were excluded. Association testing was run using SNPTEST v2.5 (Marchini et al., 2007), a genotype thresholded regression model and with adjustment using the first 10 principal components.

## 1.6 STANFORD AND EUROPEAN NARCOLEPSY COHORT WITH PRIMARY EUROPEAN ANCESTRY

These steps describe genotyping, QC and imputation for more recently enrolled Stanford narcolepsy patients of European ancestry, either from the European Narcolepsy Network or recruited at Stanford University. All cases had excessive daytime sleepiness and cataplexy or low CSF hypocretin levels and provided written informed consent. Altogether, 1,726 individuals with NT1 and 56,362 controls from the GERA cohort dbGAP (phg000583.v2) were used (Banda et al., 2015). These individuals were genotyped with Axiom® Genome-EUR chips. In addition, a smaller subset of patients with narcolepsy (N=218) and controls (N=358, total n=576) were recruited and genotyped with the Axiom PMRA chip. Raw CEL files were imported into the Affymetrix Genotyping console. Genotype calls were exported as plink ped/map files. Stringent QC was applied and markers and individuals with >0.01 missingness excluded, related individuals with identity by descent pihat>0.2 excluded and markers with HWE $P<10^{-6}$ excluded. Population covariates were extracted using plink. Post QC, genotypes were phased one chromosome at a time with SHAPEIT using the build37 coordinate system of the human genome. Phased haplotypes were then subject to imputation to 1000 Genomes phase 1 in 1mb chunks for each chromosome using IMPUTE2, or with haplotype reference consortium reference as implemented at the Michigan imputation server (https://imputationserver.sph.umich.edu/index.html). Association testing was run using SNPTEST v2.5 (Marchini et al., 2007) and a genotype thresholded regression model with adjustment for the first 10 principal components.

## 1.7 Data from FinnGen

FinnGen (www.finngen.fi/en) is a joint research project launched in Finland in the autumn of 2017, that aims to genotype 500,000 Finns, and that includes prospective and retrospective epidemiological and disease-based cohorts as well as hospital biobank samples. FinnGen combines genome data with longitudinal health care registries using unique personal identification codes allowing data collection and follow-up over the whole life span. FinnGen data release 8 is composed of 342,499 Finnish participants. We used ICD-10 code G47.4 and ICD-9 code 347 for narcolepsy. This code includes individuals who have narcolepsy with cataplexy. Phenotypes for

autoimmune diseases and asthma were obtained from FinnGen for all participants. These included asthma, type-1 diabetes mellitus, psoriasis, rheumatoid arthritis, hypothyroidism, primary biliary cholangitis, multiple sclerosis, alopecia areata, coeliac disease, inflammatory bowel disease, systemic lupus erythematosus, Crohn's disease, ulcerative colitis and Sjogren's syndrome. Patients and control subjects in FinnGen provided informed consent for biobank research, based on the Finnish Biobank Act. Alternatively, separate research cohorts, collected prior the Finnish Biobank Act came into effect (in September 2013) and start of FinnGen (August 2017), were collected based on study-specific consents and later transferred to the Finnish biobanks after approval by Fimea (Finnish Medicines Agency), the National Supervisory Authority for Welfare and Health. Recruitment protocols followed the biobank protocols approved by Fimea. The Coordinating Ethics Committee of the Hospital District of Helsinki and Uusimaa (HUS) statement number for the FinnGen study is Nr HUS/990/2017. The FinnGen study is approved by Finnish Institute for Health and Welfare (permit numbers: THL/2031/6.02.00/2017, THL/1101/5.05.00/2017, THL/341/6.02.00/2018, THL/2222/6.02.00/2018, THL/283/6.02.00/2019, THL/1721/5.05.00/2019 and THL/1524/5.05.00/2020), Digital and population data service agency (permit numbers: VRK43431/2017-3, VRK/6909/2018-3, VRK/4415/2019-3), the Social Insurance Institution (permit numbers: KELA 58/522/2017, KELA 131/522/2018, KELA 70/522/2019, KELA 98/522/2019, KELA 134/522/2019, KELA 138/522/2019, KELA 2/522/2020, KELA 16/522/2020), Findata permit numbers THL/2364/14.02/2020, THL/4055/14.06.00/2020,,THL/3433/14.06.00/2020, THL/4432/14.06/2020, THL/5189/14.06/2020, THL/5894/14.06.00/2020, THL/6619/14.06.00/2020, THL/209/14.06.00/2021, THL/688/14.06.00/2021, THL/1284/14.06.00/2021, THL/1965/14.06.00/2021, THL/5546/14.02.00/2020, THL/2658/14.06.00/2021, THL/4235/14.06.00/2021 and Statistics Finland (permit numbers: TK-53-1041-17 and TK/143/07.03.00/2020 (earlier TK-53-90-20) TK/1735/07.03.00/2021). The Biobank Access Decisions for FinnGen samples and data utilized in FinnGen Data Freeze 8 include: THL Biobank BB2017_55, BB2017_111, BB2018_19, BB_2018_34, BB_2018_67, BB2018_71, BB2019_7, BB2019_8, BB2019_26, BB2020_1, Finnish Red Cross Blood Service Biobank 7.12.2017, Helsinki Biobank HUS/359/2017, Auria Biobank AB17-5154 and amendment #1 (August 17 2020), AB20-5926 and amendment #1 (April 23 2020), Biobank Borealis of Northern Finland_2017_1013, Biobank of Eastern Finland 1186/2018 and amendment 22 § /2020, Finnish Clinical Biobank Tampere MH0004 and amendments (21.02.2020 & 06.10.2020), Central Finland Biobank 1-2017 and Terveystalo Biobank STB 2018001.

# 2. MAIN ANALYSES

## 2.1 Analysis of P-value distribution

To understand p-value distribution between observed associations in the meta-analysis and those from expected distribution, we computed the quantile quantile plot (Supplementary figure 1) and estimated inflation of p-values using the qqman package implemented in R. Analysis of genomic inflation showed inflation of LambdaGC =1.06.

**Supplementary Figure 1.** QQ-plot of narcolepsy meta-analysis across all cohorts. Raw P-values are reported using two-sided fixed effects meta-analysis.

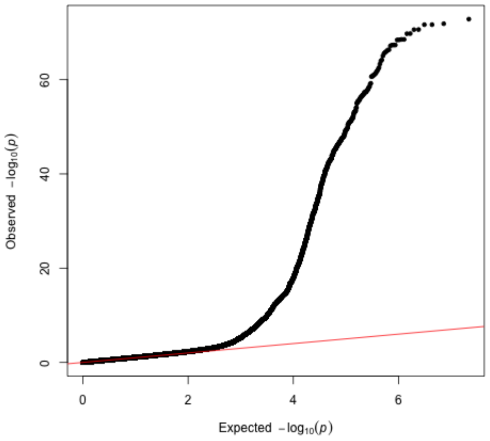


## 2.2 Regional association and Zoom plots

For each genetic locus reaching genome-wide significance, variants in LD were examined using locusZoom (Pruim et al., 2010). This was done to exclude potential outlier variants and to confirm each signal was genuine. For additional descriptions on the functions of these loci, please refer to Supplementary Table 3. Below we draw locuszoom plots for variants of interest reported in this manuscript as **Supplementary Figure 2A-M.** Raw P-values are reported using two-sided fixed effects meta-analysis.

**Supplementary Figure 2A: TNFSF4 locus**

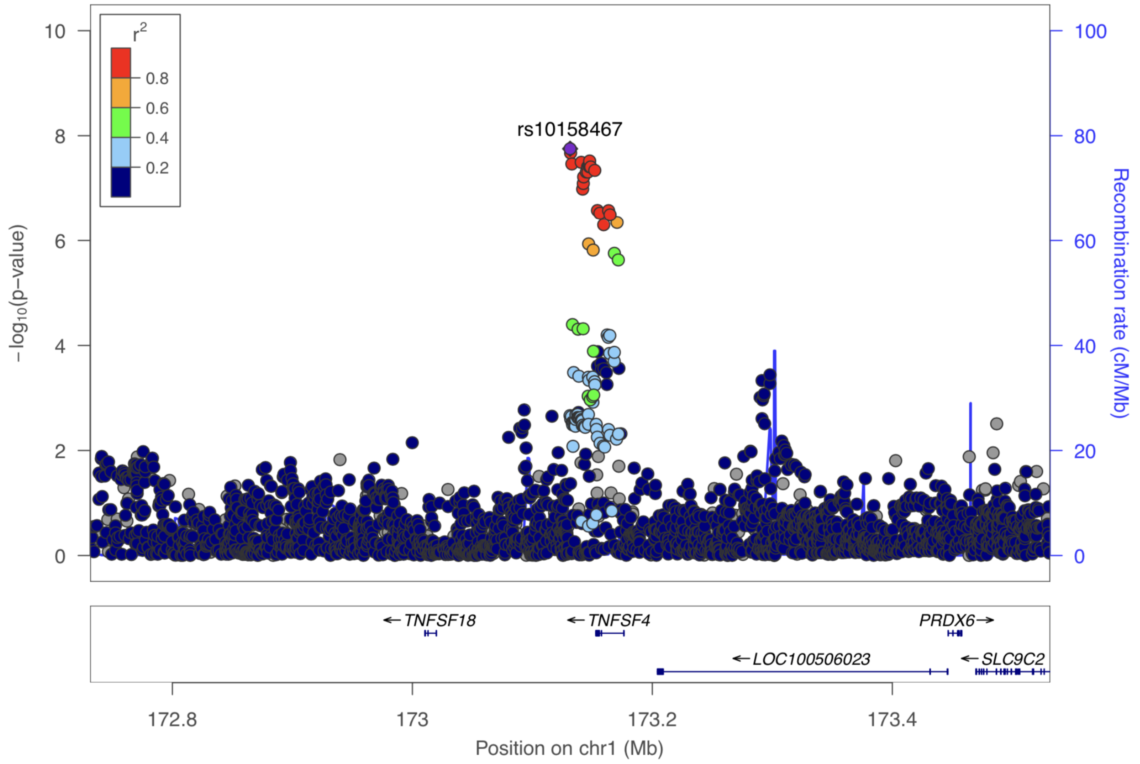

**Supplementary Figure 2B: DENND1B locus**

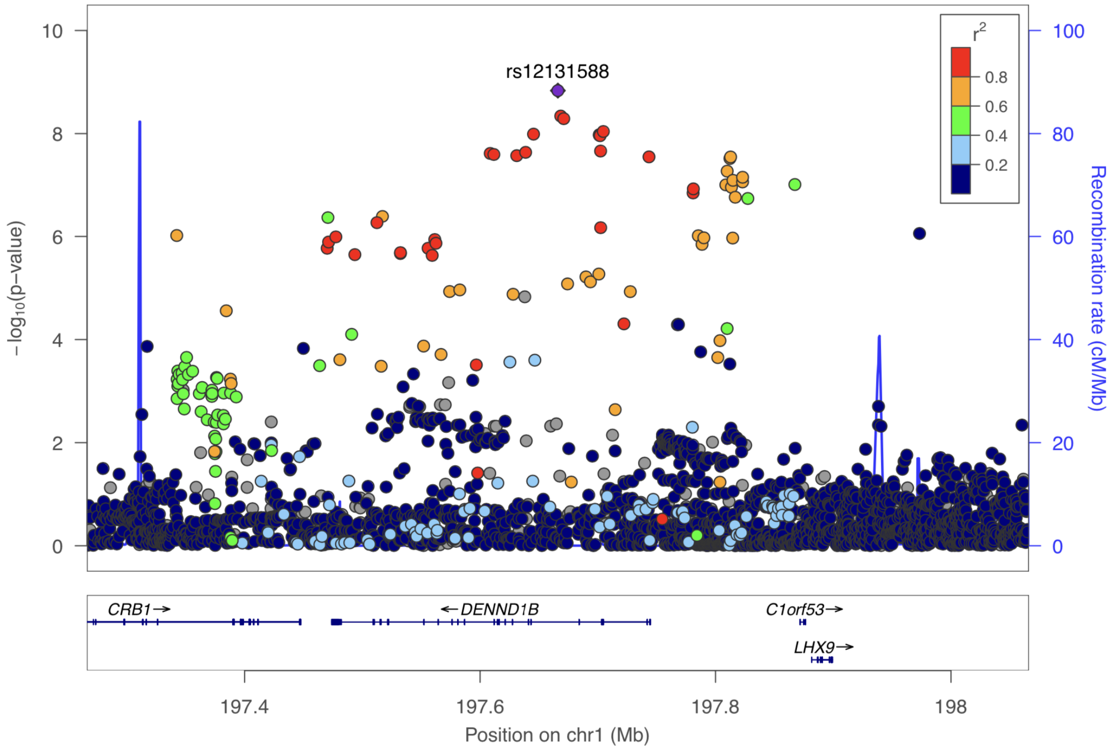


**Supplementary Figure 2C: CD207 locus (langerin)** (Binned raw two-sided P-values are reported from logistic regression.)

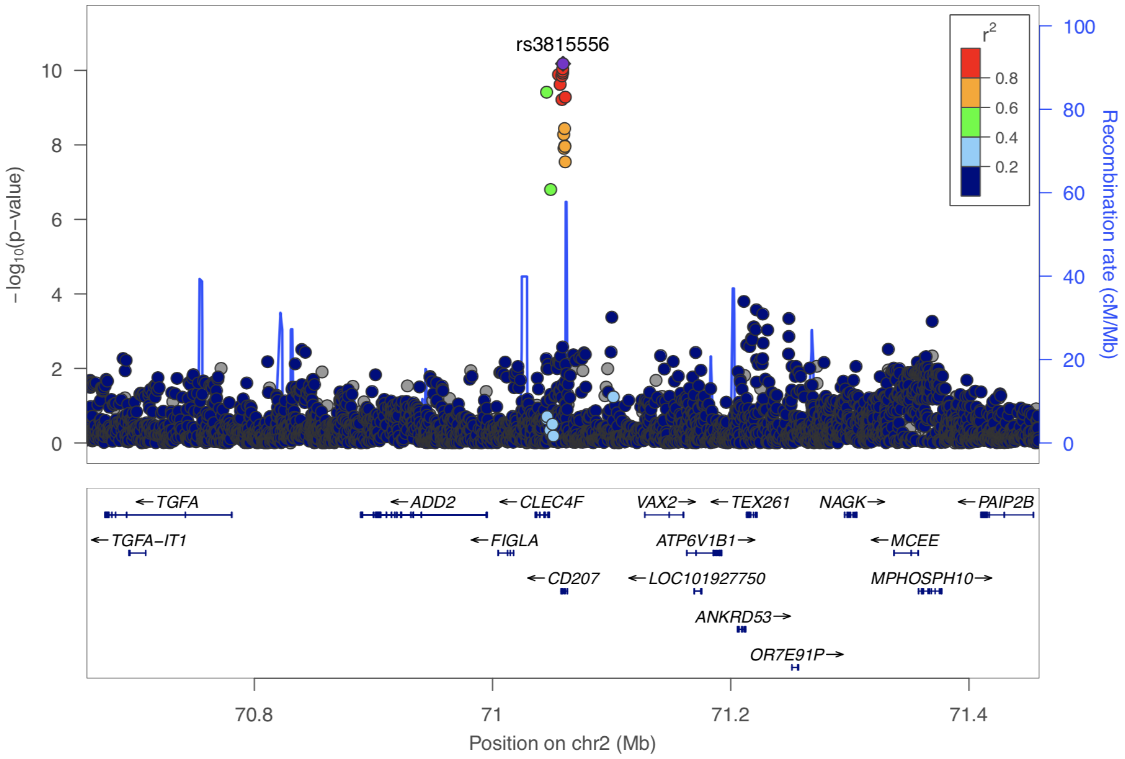

## Supplementary Figure 2D: NAB1 locus

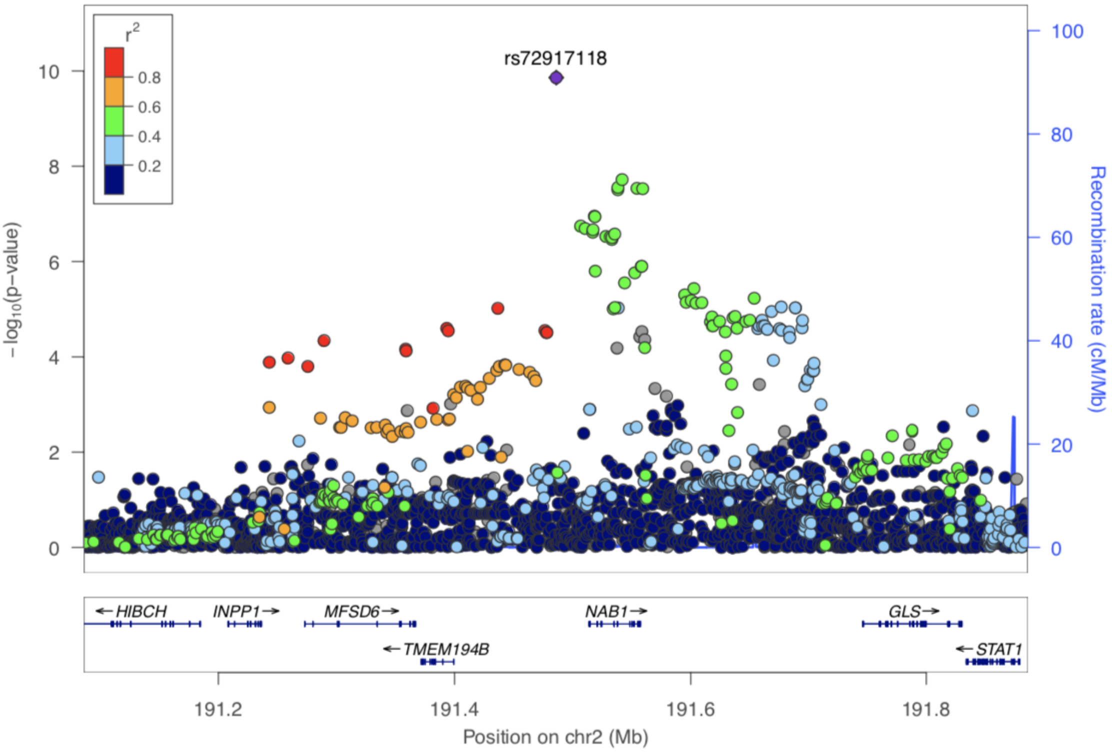


## Supplementary Figure 2E: TRB locus

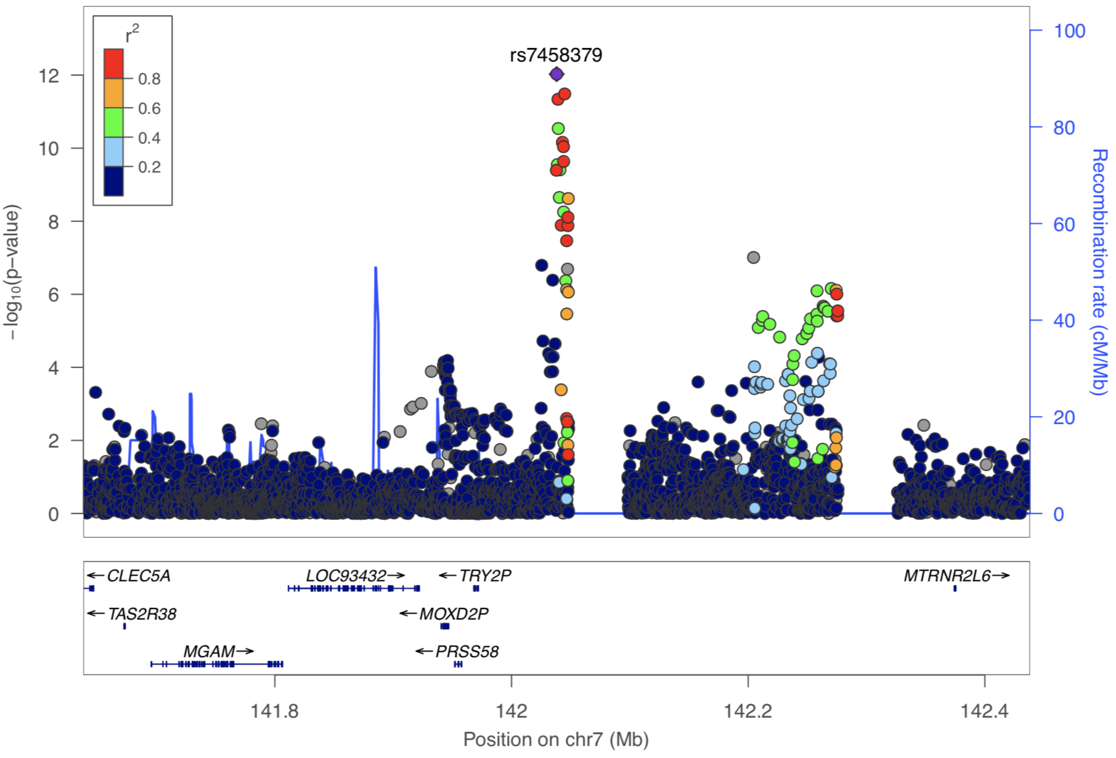

## Supplementary Figure 2F: ZNF365 locus

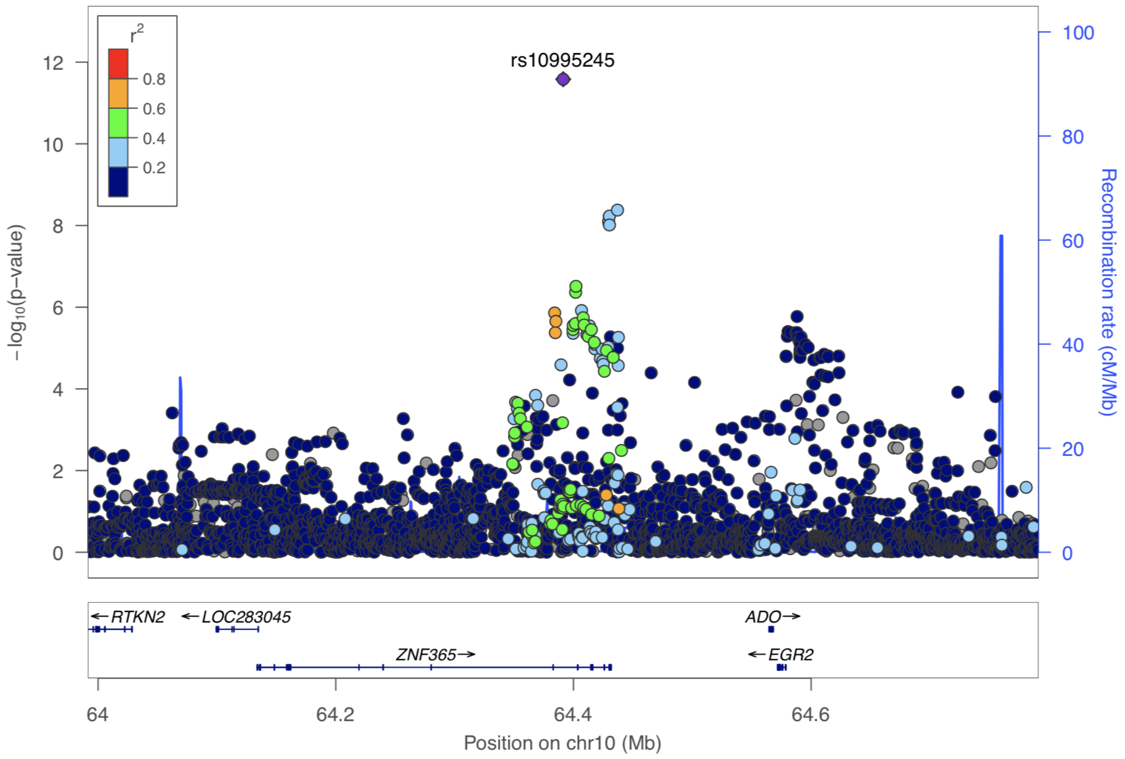


## Supplementary Figure 2G: CTSC locus

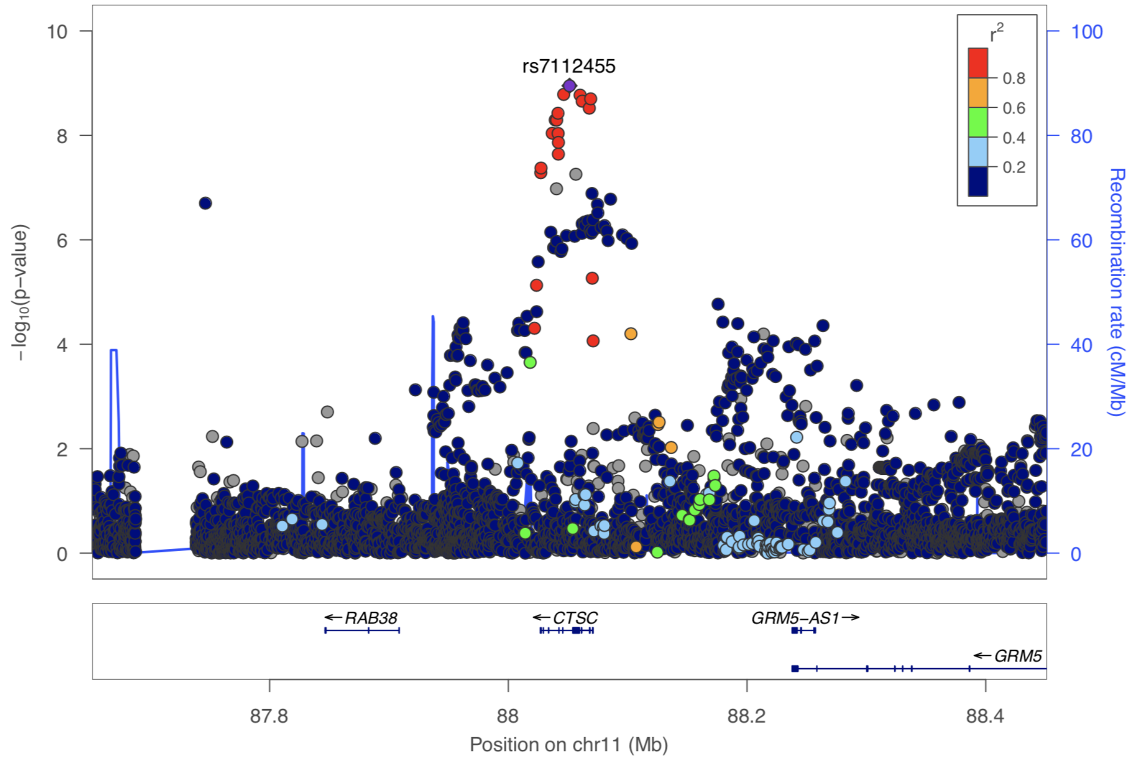


## Supplementary Figure 2H: IKZF4-ERBB3 locus

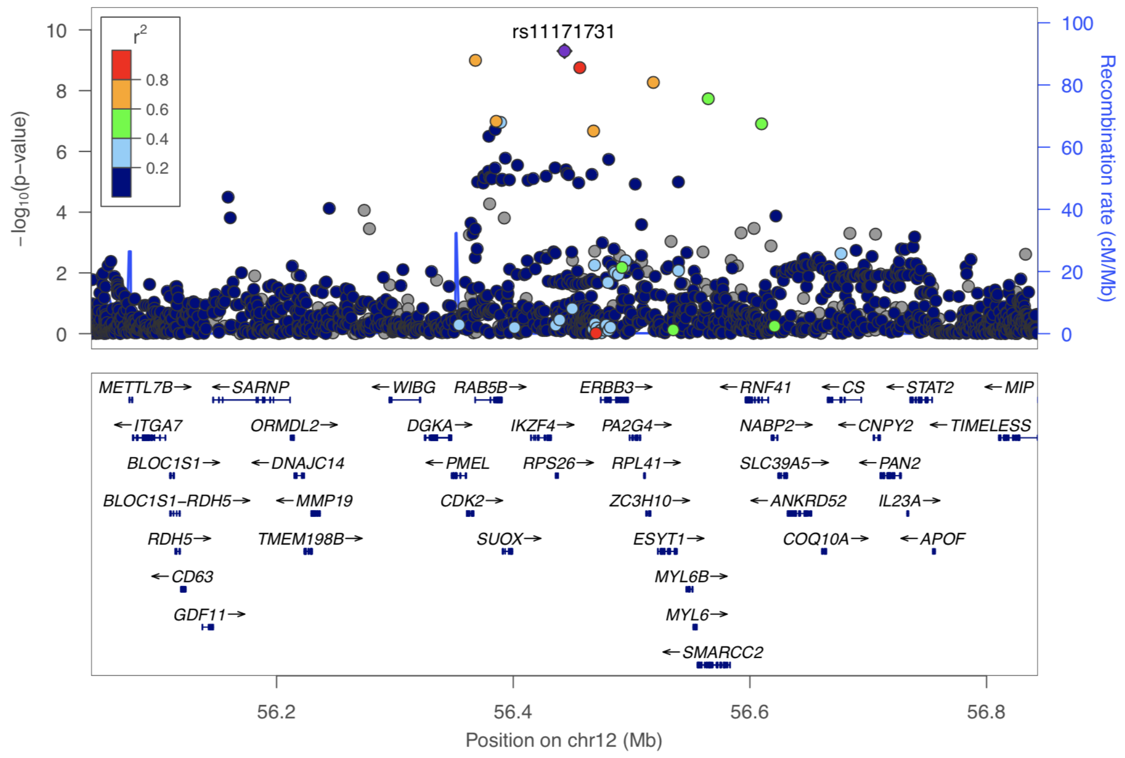


## Supplementary Figure 2I: TRA locus

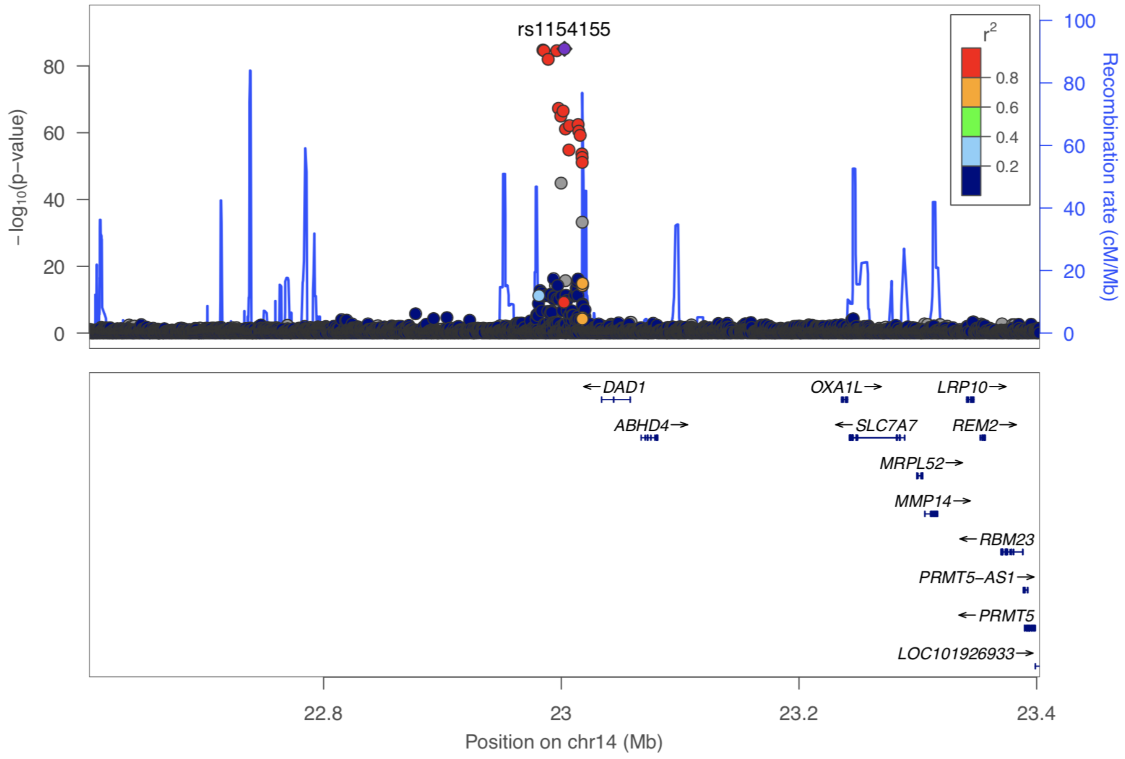

## Supplementary Figure 2J: CTSH locus

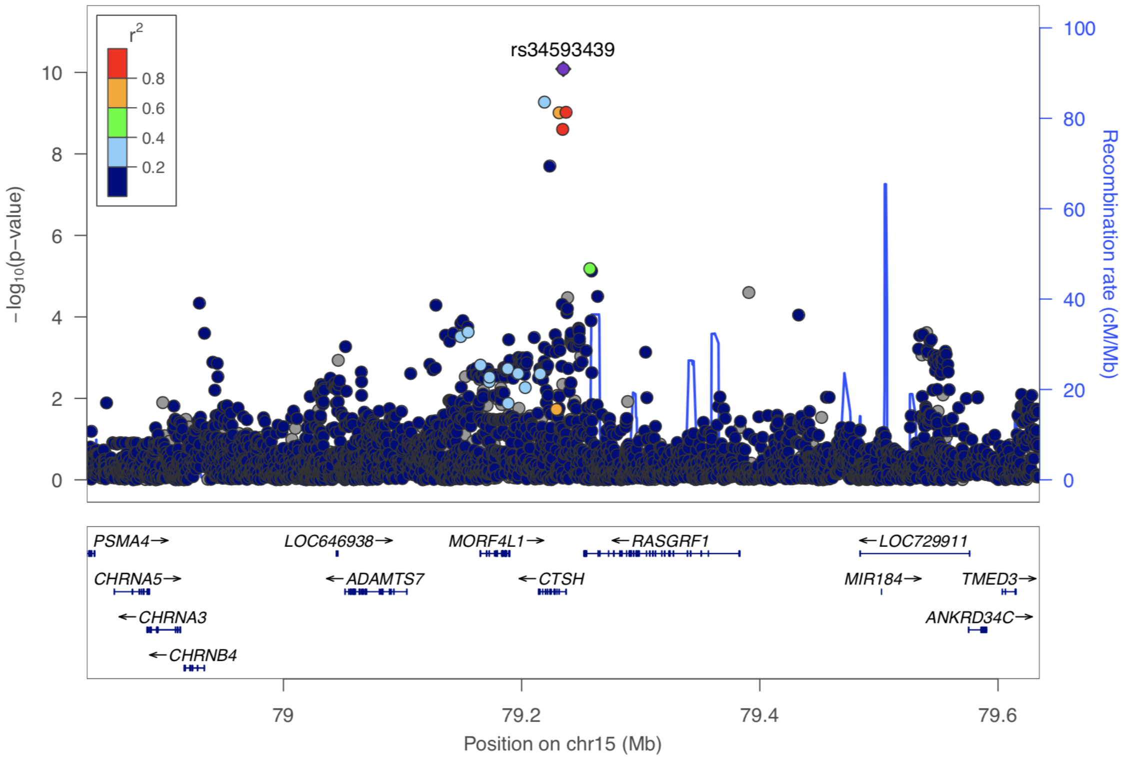


## Supplementary Figure 2K: SIRPG locus

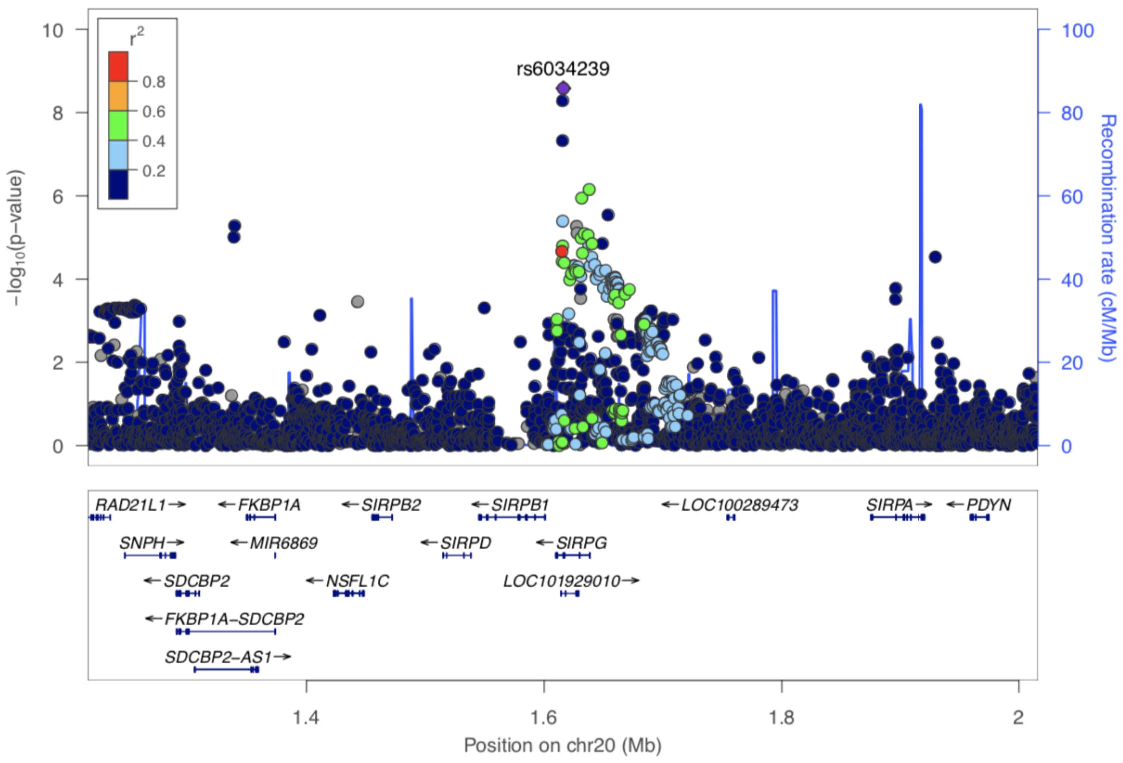

## Supplementary Figure 2L: IFNAR1 locus

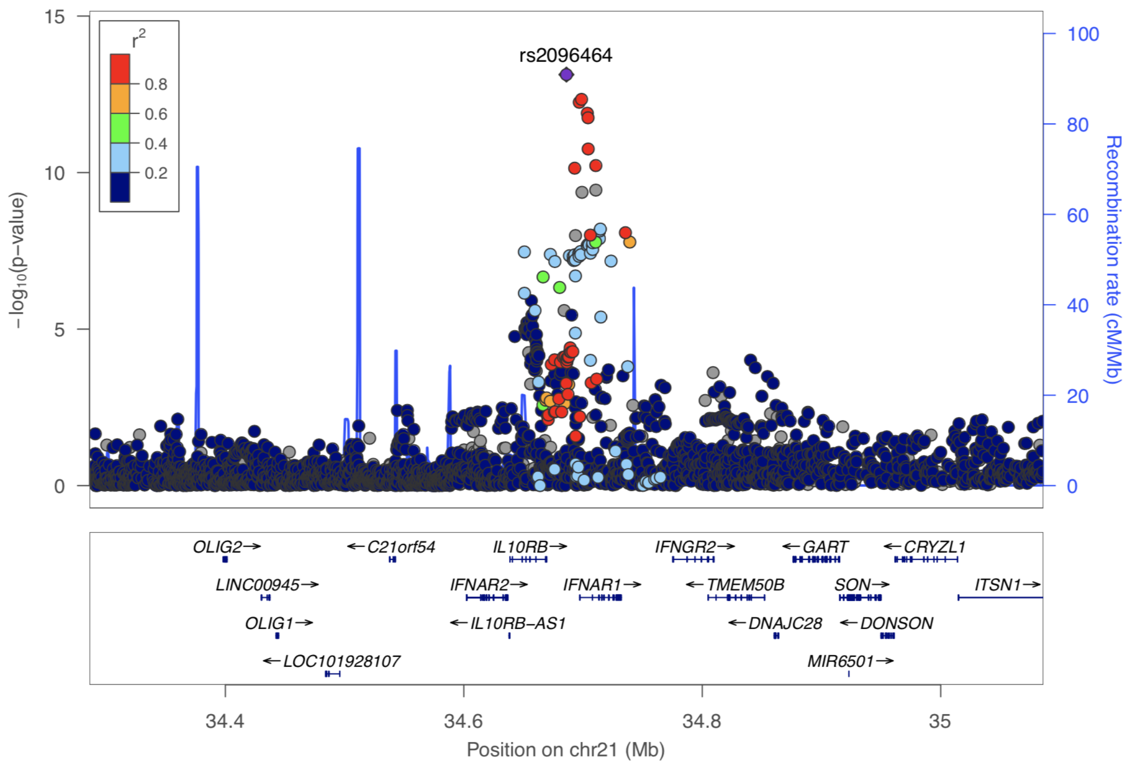


## Supplementary Figure 2M: PRF1 locus

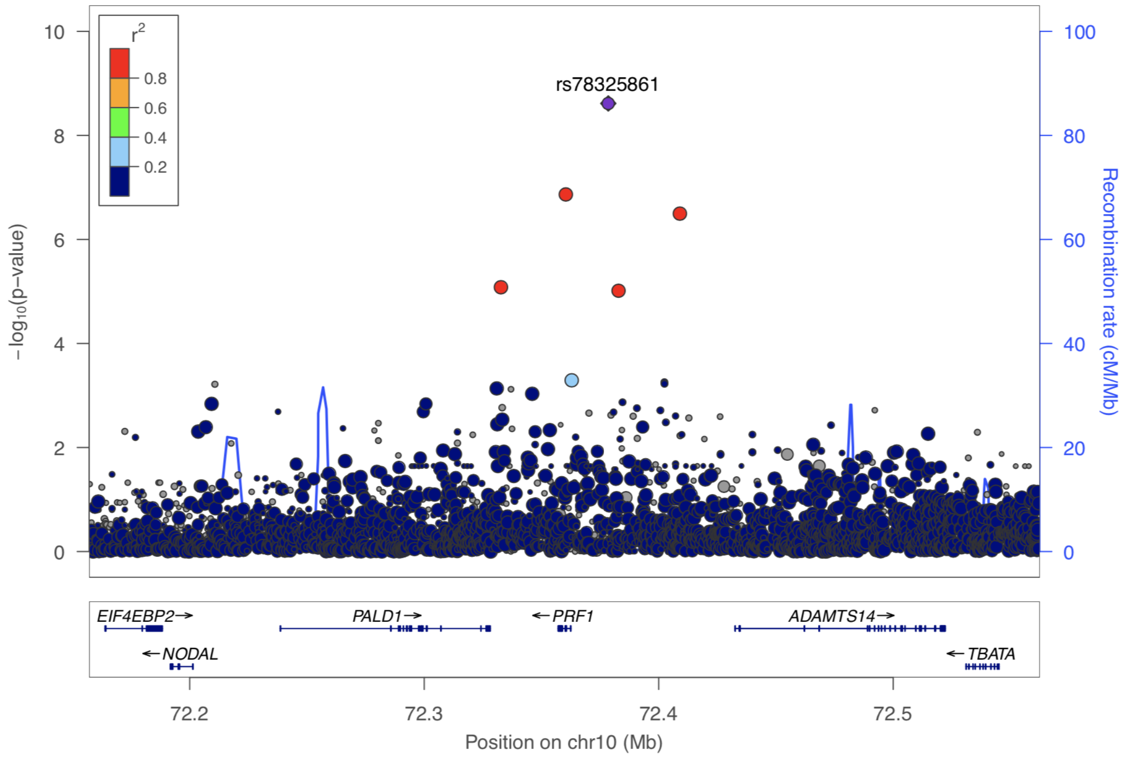

## 2.3 Vaccination-related narcolepsy

**Supplementary Figure 3.** In 2009-2010, both the H1N1 pandemic and vaccination with GSK vaccine Pandemrix® was associated with an increased incidence of narcolepsy. It was possible that individuals with vaccination-related narcolepsy had a different genetic predisposition than type-1 cases without H1N1 vaccination. To test this hypothesis, we obtained 245 individuals with vaccination-related narcolepsy. Genetic associations in this relatively small sample identified HLA and TRA as genome wide significant association, mirroring data obtained in all other cases. Many other genetic effects present in non-vaccination cases were also nominally significant (table 1).

For visualization we show **A.** the Manhattan plot of vaccination related narcolepsy, which finds association with TRA on chromosome 14 and HLA locus on chromosome 6. **B.** QQ plot of Vaccination-related narcolepsy. **C.** Variance explained by PRS (polygenic risk scores) built on the non-vaccinated narcolepsy cohort when used to predict on the vaccination cohort, the y-axis is the % variance explained by the PRS, while the x-axis represents the p-value thresholds. Note extremely high significance of overlap. **C.** Variance explained by narcolepsy/non vaccinated Polygenic risk scores profiles on post vaccination narcolepsy cohort. Vaccination-triggered polygenic risk score (PRS) correlates strongly with the PRS of other narcolepsy cases. A PRS was built using the narcolepsy (non-vaccinated) discovery cohort and tested in vaccination-related cases from Finland (pseudo R2). The Y-axis represents R2 and the X-axis P-value thresholds. The color gradient over each bar is representative of the P value of the association.

A.

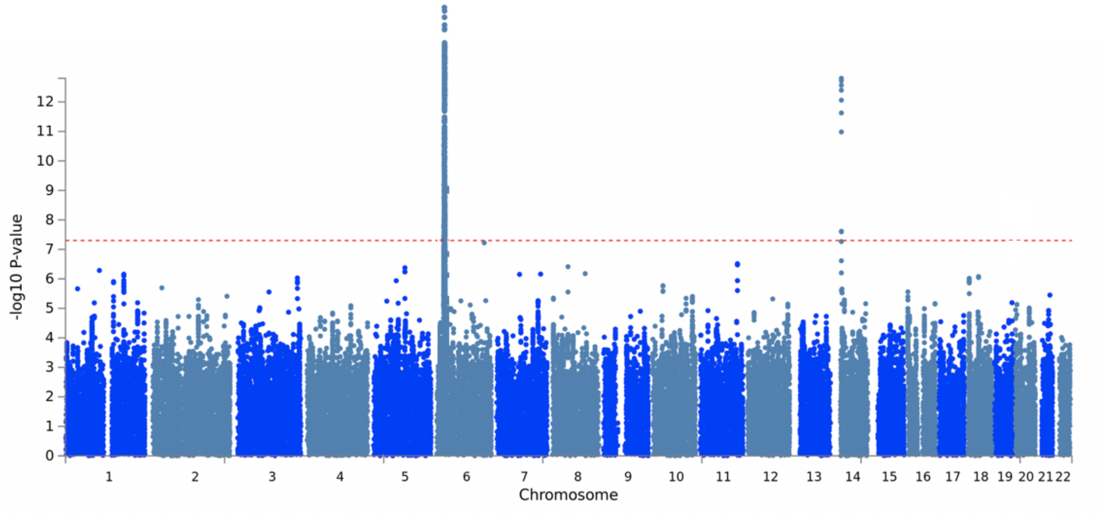

**B.**

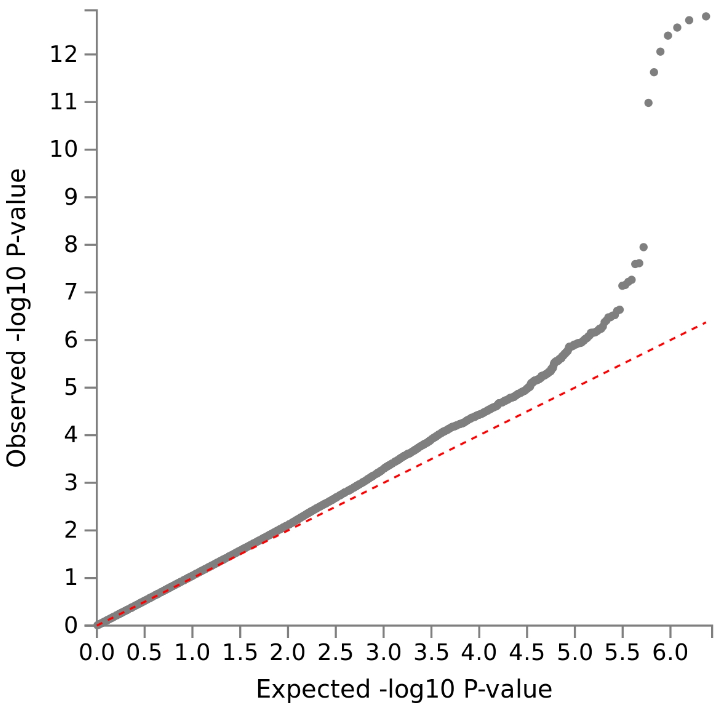
Observed -log10 P-value
Expected -log10 P-value
0 1 2 3 4 5 6 7 8 9 10 11 12
0.0 0.5 1.0 1.5 2.0 2.5 3.0 3.5 4.0 4.5 5.0 5.5 6.0

**C.**

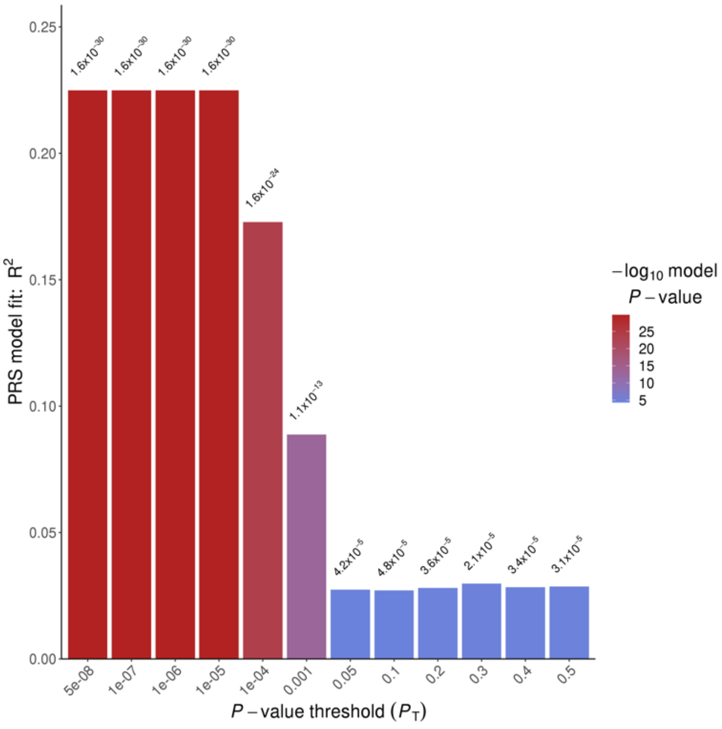
PRS model fit: $R^2$
0.00 0.05 0.10 0.15 0.20 0.25
1.6x10$^{-30}$
1.6x10$^{-30}$
1.6x10$^{-30}$
1.6x10$^{-30}$
1.6x10$^{-24}$
1.1x10$^{-13}$
4.2x10$^{-5}$
4.8x10$^{-5}$
3.6x10$^{-5}$
2.1x10$^{-5}$
3.4x10$^{-5}$
3.1x10$^{-5}$
5e-08 1e-07 1e-06 1e-05 1e-04 0.001 0.05 0.1 0.2 0.3 0.4 0.5
$P$ − value threshold ($P_T$)
$-\log_{10}$ model
$P$ − value
25
20
15
10
5

## 2.4 *IFNAR1* eQTL in dendritic cells infected with influenza

We hypothesized that the SNP associated with T1N would affect IFN type 1 response of dendritic cells when infected with influenza. LD was examined using locusZoom (Pruim et al., 2010). The locuszoom plot below shows variants of the IFNAR1 locus in narcolepsy (left) and variants of the IFNAR1 locus associated with IFNAR1 expression, following influenza-A H1N1 infection in dendritic cells (right). We thereby show that the leading narcolepsy risk variant rs2409487 is in perfect LD with the lead in Influenza-A infection eQTL (rs6517159) in dendritic cells and colocalization analysis suggests shared signal.

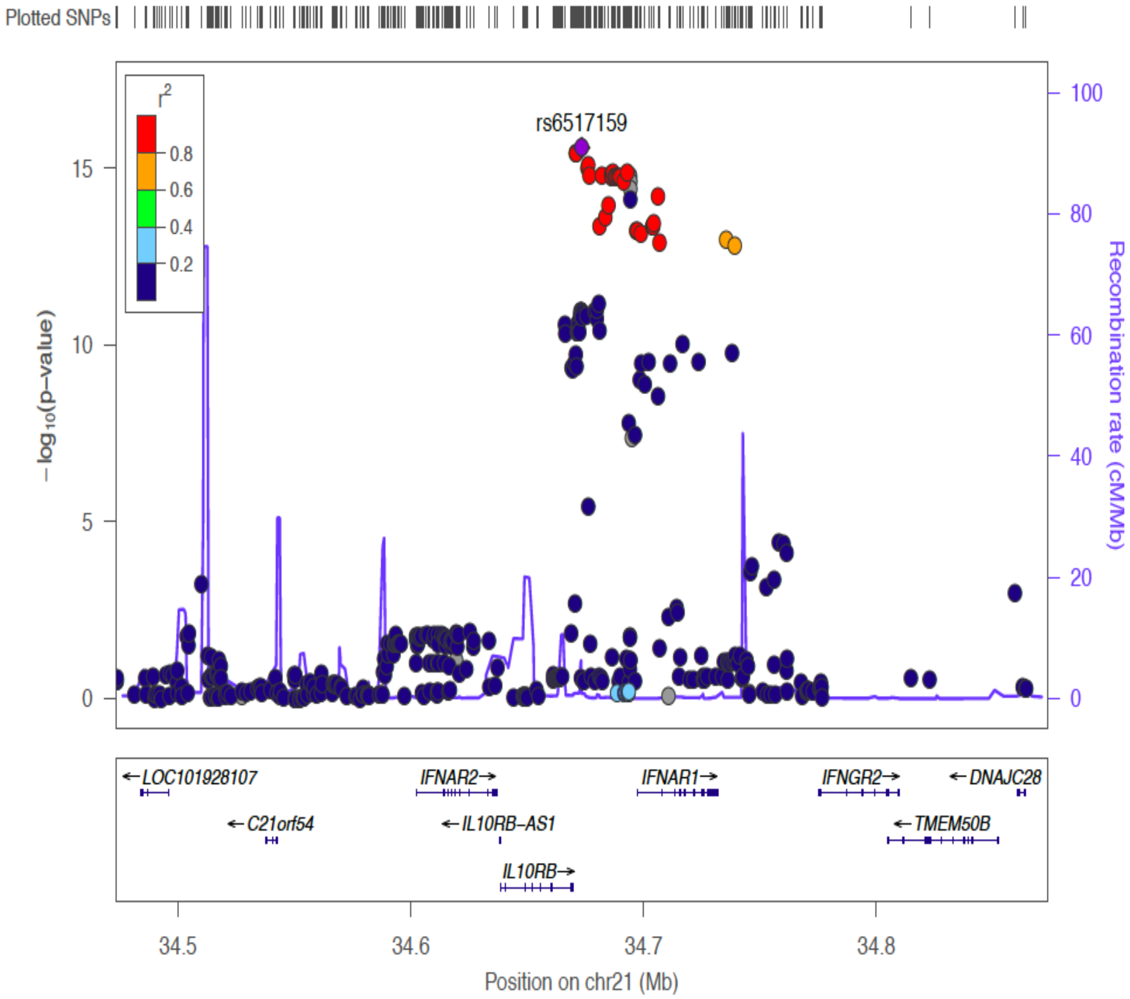


## 2.5 Functional analysis of T cell receptors

We examined functional contribution of TCR genetic variants associated with NT1 on T cell receptor chain usage using targeted RNA expression sequencing data from 60 patients and 60 DQ0602 controls, as well as from a larger RNA sequencing in a population data of 895 individuals available from Battle et al. (2015). In the smaller targeted sequencing data, we first examined the ratio of TRA J24F and L alleles in memory $CD4^+$ and $CD8^+$ Cytotoxic T cells. We did not see any differences in ratios between TRA J24 chain usage between the cell populations. This indicates that the effect of this polymorphism is present in both $CD4^+$ and $CD8^+$ cells.

**Supplementary Figure 5.** Expression level as Ratio of F vs. L allele in TRA J24 in different T cell subsets.

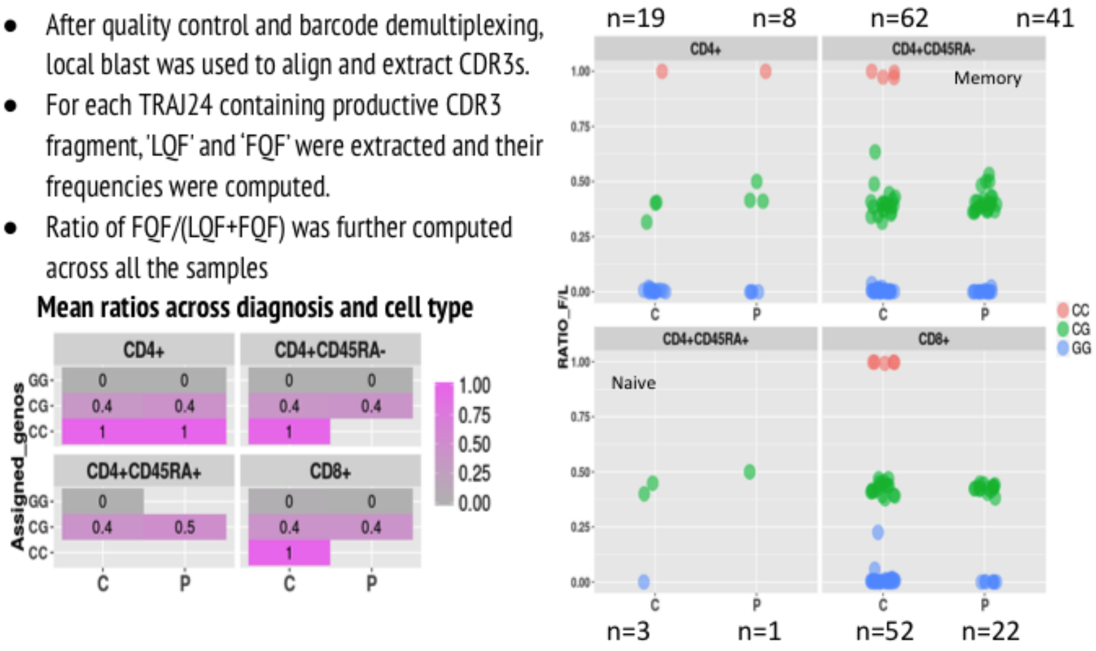


At the genetic level, we also performed conditional analysis for the main effect at TRA and TRB loci, e.g. we adjusted the main analysis for the lead variant. In TRA essentially all association were not significant after adjusting for TRA locus lead variant rs1154155.

**Supplementary Figure 6.** Analysis of TRA locus after and before adjustment with rs1154155. Raw P-values are reported using two-sided fixed effects meta-analysis.

Before adjustment

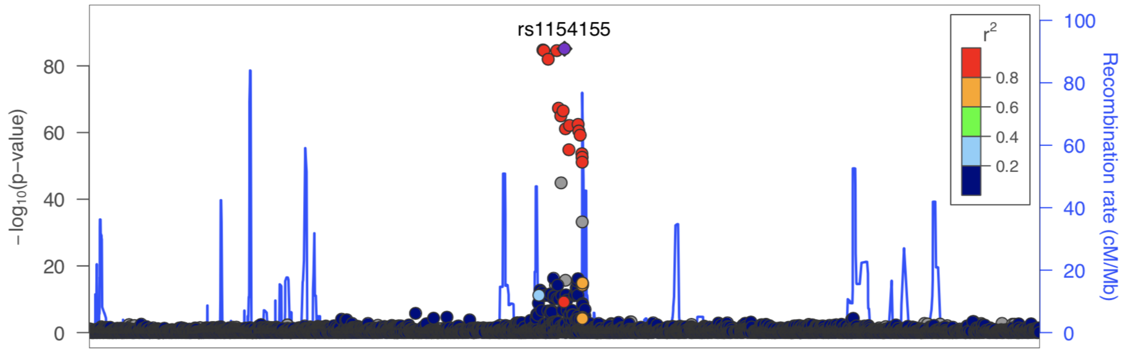

\After adjustment with rs1154155

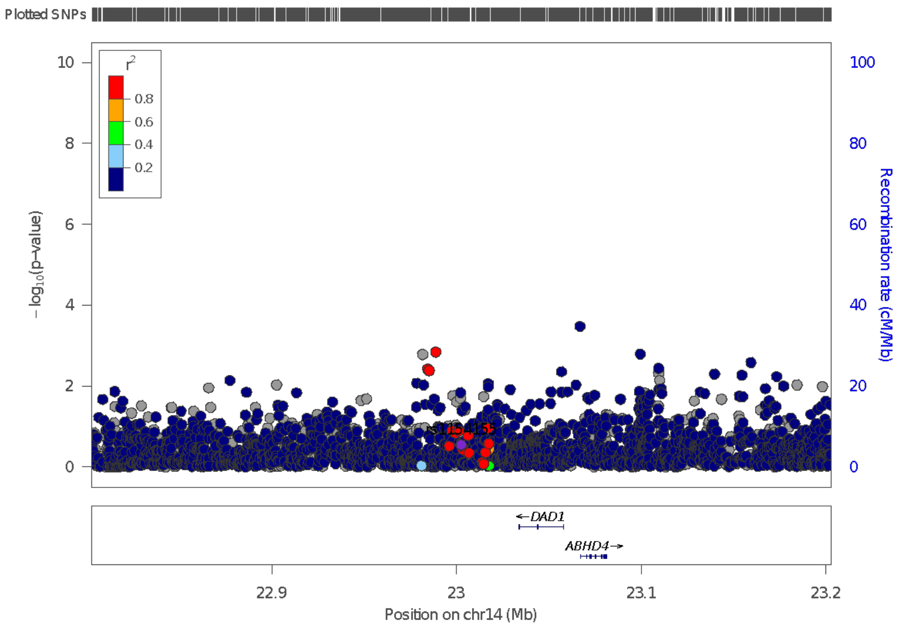


At the TRB locus level, LD is more complex and the most significant variants that associate with NT1 are part of two main haplotypes.

**Supplementary Figure 7**. Linkage disequilibrium of variants that are significantly associated with TRBV4-2 usage. The main effects of rs1008599 and rs7458379 are on TRBV4-2 usage. Linkage disequilibrium using data from the 1000 Genomes by r2 is reported.

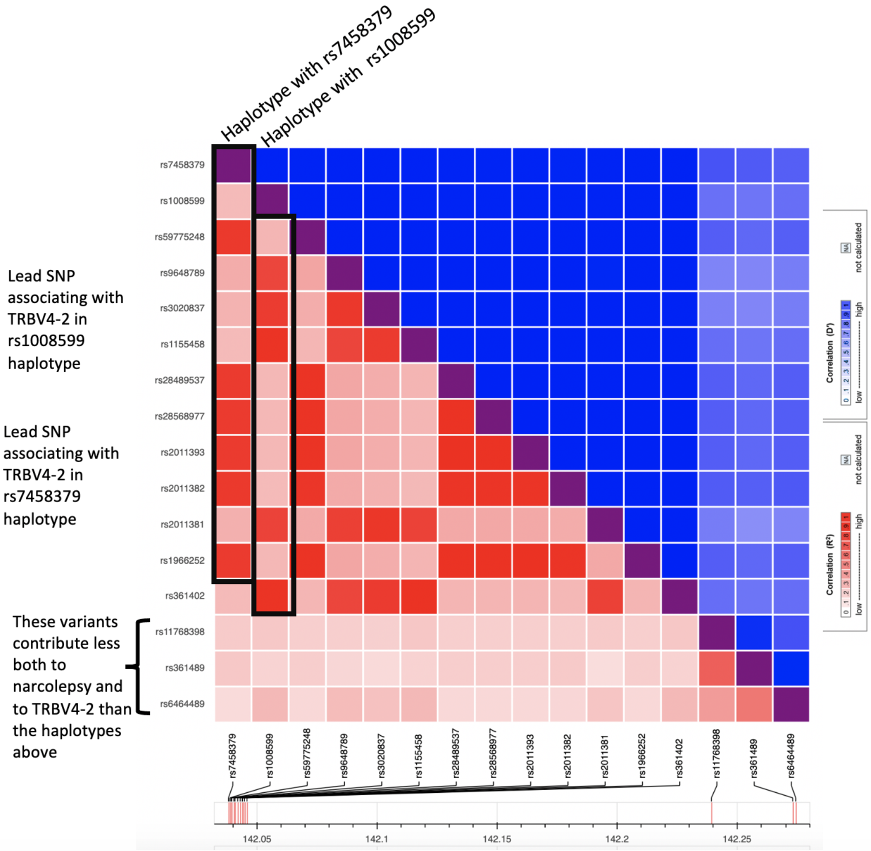

Consequently, conditioning for the main effect removes most of the association.

**Supplementary Figure 8:** TRB association no conditioning. Raw P-values are reported using two-sided fixed effects meta-analysis.

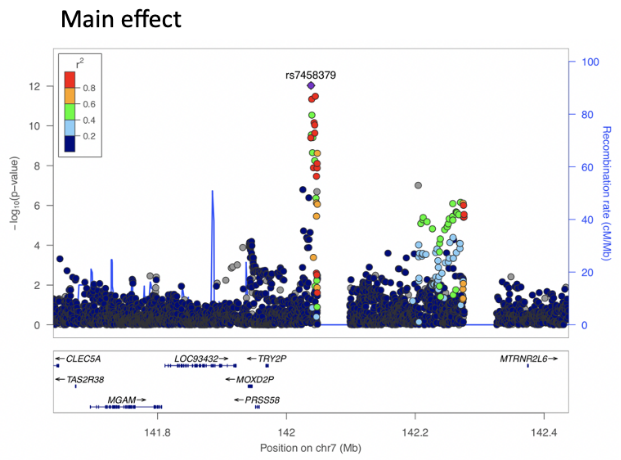


TRB association adjusted for the lead variants that are part of the main haplotypes. Adjusting for these variants, and especially for rs1008599, abolishes most of the association at the TRB locus.

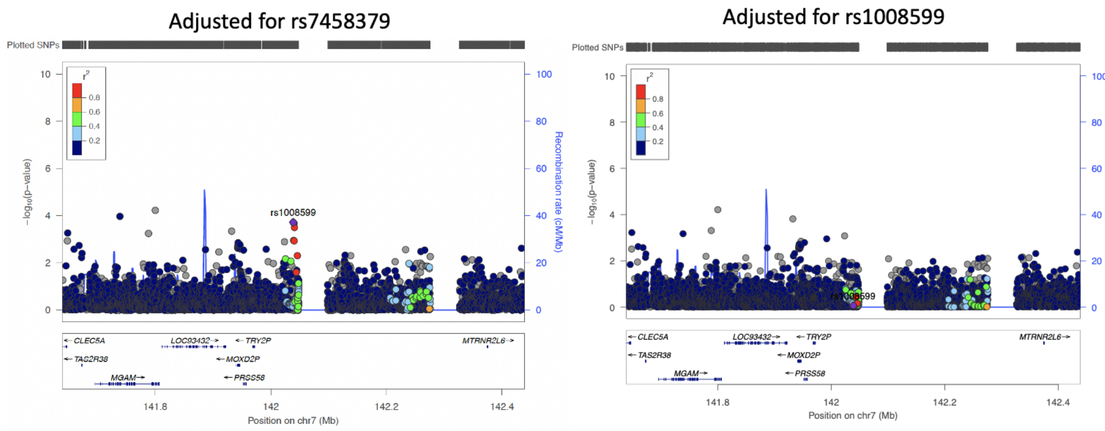


## TRA and TRB chain usage at the population level

As part of the main text, we also show how variants at the TRA and TRB loci affect receptor chain usage in the larger Battle et al. sample. We observe similar eQTLs at the population level as were observed in our own sequencing data. We present below the plots generated from these analyses and provide association statistics in supplementary tables.

**Supplementary Figure 9:** TRA locus rs1154155 and TRAJ28 expression. The center of the boxplot corresponds to the median corresponding to 50th percentile, the box indicates the upper and lower bounds of the interquartile range corresponding to 25th and 75th percentile, and the min and max values correspond to the plus/minus two interquartile ranges. The shaded area corresponds to 95% confidence interval from a linear fit, two-sided P-value derived from a linear fit. N = 130.

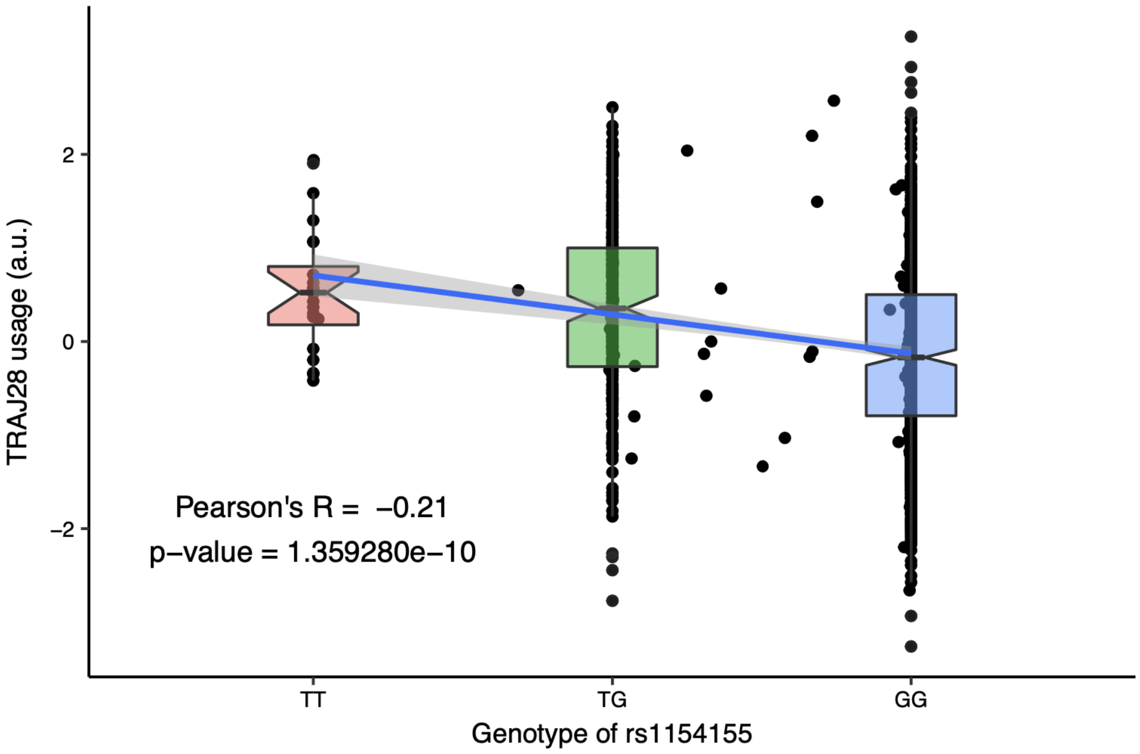

**Supplementary Figure 10:** TRA locus rs1154155 and TRAJ24 expression. The center of the boxplot corresponds to the median corresponding to 50th percentile, the box indicates the upper and lower bounds of the interquartile range corresponding to 25th and 75th percentile, and the min and max values correspond to the plus/minus two interquartile ranges. The shaded area corresponds to 95% confidence interval from a linear fit, two-sided P-value derived from a linear fit. N = 130.

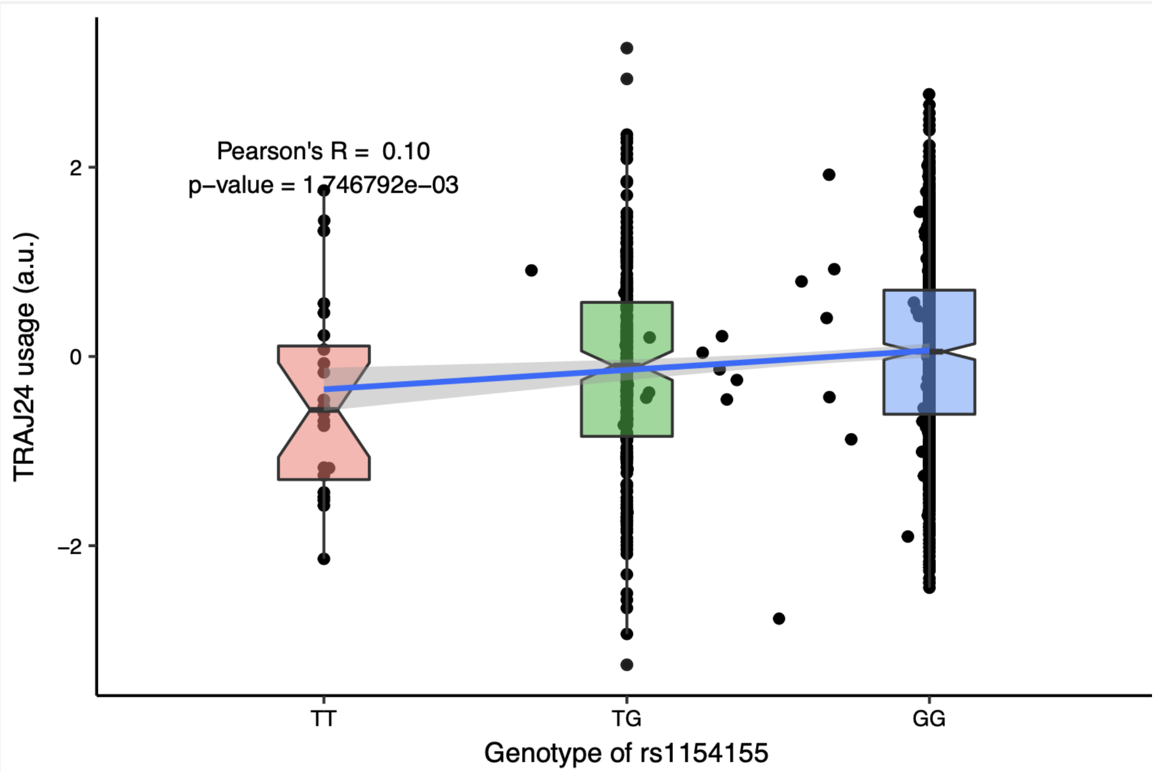

**Supplementary Figure 11:** TRB locus rs1008599 and TRBV4-2 expression. The center of the boxplot corresponds to the median corresponding to 50th percentile, the box indicates the upper and lower bounds of the interquartile range corresponding to 25th and 75th percentile, and the min and max values correspond to the plus/minus two interquartile ranges. The shaded area corresponds to 95% confidence interval from a linear fit, two-sided P-value derived from a linear fit. N = 130.

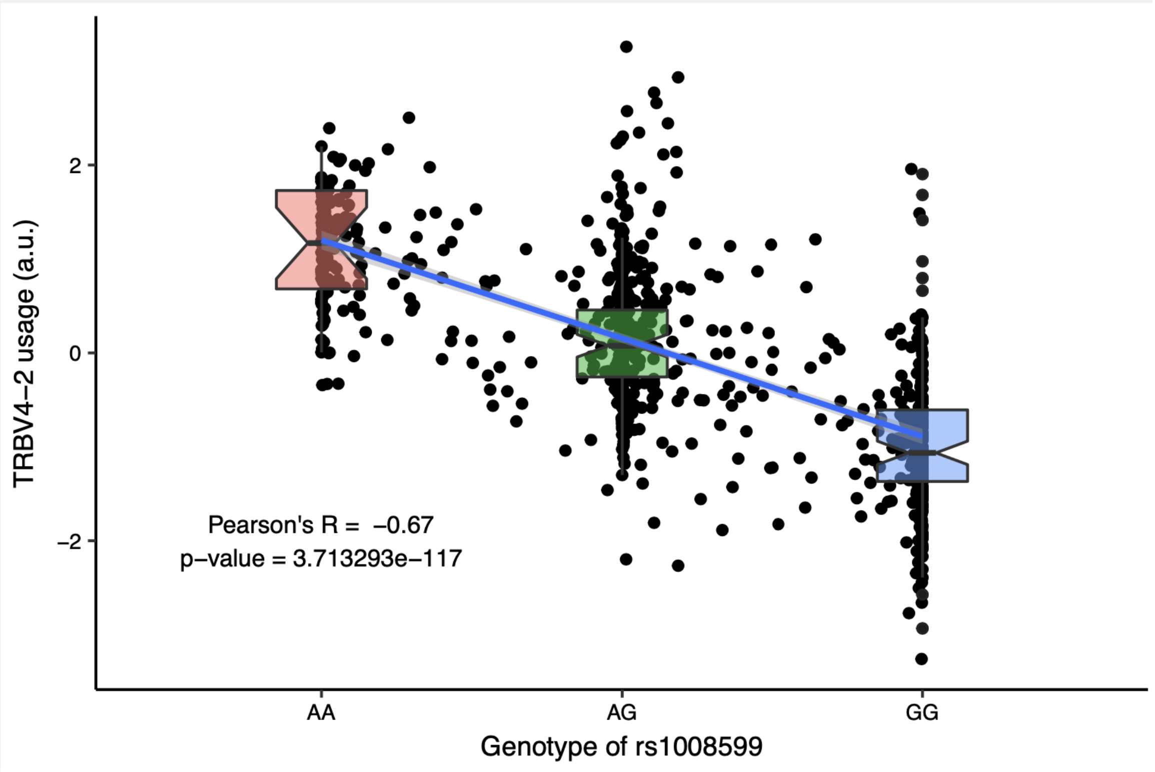

**Supplementary Table 1. Sample description for genetic analysis in type-1 narcolepsy**

| Study population | Ethnicity | N cases | N controls | Reference |
|---|---|---|---|---|
| East Asian Stanford Narcolepsy cohort | East Asian | 528 | 2434 | . |
| Han et al., 2013 narcolepsy samples | East Asian | 1497 | 1992 | Han et al., 2013 |
| Japanese narcolepsy cohort | East Asian | 387 | 1476 | Toyoda et al., 2015 |
| Stanford Narcolepsy samples with primary African Ancestry | African American | 213 | 1747 | . |
| European Narcolepsy Network | Caucasian | 472 | 702 | . |
| Hallmayer et al., 2009 narcolepsy cohort #1 | Caucasian | 182 | 167 | Hallmayer et al., 2009 |
| Hallmayer et al., 2009 narcolepsy cohort #2 | Caucasian | 625 | 915 | Hallmayer et al., 2009 |
| Stanford samples with primary European Ancestry, Second collection | Caucasian | 1726 | 56362 | . |
| Stanford samples with primary European Ancestry, Third collection | Caucasian | 218 | 358 | . |
| ***Total*** | | ***5848*** | ***66153*** | . |
| **Samples with vaccination-related narcolepsy** | | | | |
| Swedish, Pandemrix | Caucasian | 32 | 4901 | Hallberg et al., 2019 |
| Norwegian, Pandemrix | Caucasian | 75 | 2796 | Juvodden et al., 2020 |
| Irish, Pandemrix | Caucasian | 37 | 10586 | . |
| Finnish, Pandemrix | Caucasian | 81 | 420 | . |
| Total vaccination-related | | 225 | 18703 | . |
| ***Total all*** | | ***6073*** | ***84856*** | . |

**Supplementary Table 1:** Sample description for genetic analysis in type-1 narcolepsy. Information for number of individuals with type 1 narcolepsy (N cases) and disease free controls (N controls), ethnicity of the study participants and earlier publication and Pubmed ID if the cohort has been described earlier. Results from enrichment analysis from FUMA. N, total number of genes in a gene set; n, number of genes from narcolepsy analysis. Genes reflect those that were overlapping with predefined disease gene set. Raw and adjusted P-values from hypergeometric test are reported.

**Supplementary Table 2. Variant associations across ethnic groups**

| | Locus name | *TNFSF4* | *DENND1B* | *CD207* | *NAB1* | *TRB* | *ZNF365* | *PRF1* | *CTSC* | *IKZF4-ERBB3* | *TRA* | *CTSH* | *SIRPG* | *IFNAR1* |
|---|---|---|---|---|---|---|---|---|---|---|---|---|---|---|
| | CHR | 1 | 1 | 2 | 2 | 7 | 10 | 10 | 11 | 12 | 14 | 15 | 20 | 21 |
| | rsid | rs10158467 | rs12131588 | rs3815556 | rs72917118 | rs7458379 | rs10995245 | rs78325861 | rs7112455 | rs11171731 | rs1154155 | rs34593439 | rs6034239 | rs2096464 |
| | Position | 173131493 | 197666111 | 71059153 | 191486081 | 142038166 | 64391375 | 72378489 | 88051293 | 56443342 | 23002684 | 79234957 | 1616137 | 34686049 |
| | Other allele | A | G | A | C | C | G | C | T | C | T | G | G | G |
| | Effect allele | G | A | G | T | T | A | G | A | T | G | A | A | T |
| Meta-analysis all cohorts | P-value | 6.64E-08 | 3.52E-09 | 3.33E-10 | 1.03E-09 | 8.03E-12 | 4.15E-12 | 2.27E-06 | 5.58E-09 | 5.72E-09 | 2.28E-76 | 2.56E-09 | 1.21E-08 | 4.55E-14 |
| | beta | 0.159 | 0.17 | 0.246 | -0.205 | 0.155 | 0.148 | -0.338 | 0.244 | 0.147 | 0.463 | 0.215 | 0.123 | -0.179 |
| | se | 0.029 | 0.029 | 0.039 | 0.034 | 0.023 | 0.021 | 0.072 | 0.042 | 0.025 | 0.025 | 0.036 | 0.022 | 0.024 |
| | P heterogeneity across ethnic groups | 0.661 | 0.015 | 0.158 | 0.994 | 0.168 | 0.483 | 0.967 | 0.497 | 0.264 | 0.036 | 0.559 | 0.011 | 0.990 |
| Meta-analysis primary Asian ancestry | Other allele | A | G | A | C | C | G | C | T | C | T | G | G | G |
| | Effect allele | G | A | G | T | T | A | G | A | T | G | A | A | T |
| | P-value | 8.40E-02 | 0.308 | 1.18E-06 | 1.97E-05 | 0.008 | 7.82E-06 | 0.861 | 9.52E-07 | 0.005 | 3.18E-38 | 0.176 | 1.55E-07 | 4.96E-05 |
| | beta | 0.127 | 0.051 | 0.311 | -0.205 | 0.119 | 0.186 | -0.160 | 0.294 | 0.117 | 0.525 | 0.135 | 0.226 | -0.184 |
| | se | 0.073 | 0.050 | 0.064 | 0.048 | 0.045 | 0.042 | 0.917 | 0.060 | 0.041 | 0.041 | 0.100 | 0.043 | 0.045 |
| | P heterogeneity | 0.880 | 0.001 | 0.105 | 0.140 | 0.170 | 0.414 | 0.903 | 0.478 | 0.444 | 0.755 | 0.782 | 0.011 | 0.206 |
| Meta-analysis primary European ancestry | Other allele | A | G | A | C | C | G | C | T | C | T | G | G | G |
| | Effect allele | G | A | G | T | T | A | G | A | T | G | A | A | T |
| | P-value | 3.71E-06 | 2.85E-10 | 9.95E-06 | 2.52E-05 | 4.01E-08 | 2.75E-07 | 8.96E-06 | 9.34E-04 | 4.42E-08 | 2.31E-41 | 3.00E-08 | 8.33E-04 | 3.01E-10 |
| | beta | 0.157 | 0.227 | 0.234 | -0.207 | 0.149 | 0.132 | -0.323 | 0.199 | 0.187 | 0.434 | 0.220 | 0.086 | -0.177 |
| | se | 0.034 | 0.036 | 0.053 | 0.049 | 0.027 | 0.026 | 0.073 | 0.060 | 0.034 | 0.032 | 0.040 | 0.026 | 0.028 |
| | P heterogeneity | 0.518 | 0.164 | 0.899 | 0.755 | 0.476 | 0.240 | 0.003 | 0.485 | 0.520 | 2.12E-05 | 0.227 | 0.718 | 0.219 |
| Stanford Narcolepsy samples with primary African Ancestry | Other allele | A | G | A | C | C | G | C | T | C | T | G | G | G |
| | Effect allele | G | A | G | T | T | A | G | A | T | G | A | A | T |
| | P-value | 0.063 | 0.121 | 0.889 | 0.401 | 0.007 | 0.073 | 0.046 | 0.535 | 0.934 | 0.648 | 0.048 | 0.035 | 0.362 |
| | beta | 0.282 | 0.256 | 0.020 | -0.238 | 0.441 | 0.207 | -9.557 | 0.153 | -0.015 | 0.091 | 0.331 | 0.286 | -0.169 |
| | se | 0.154 | 0.162 | 0.140 | 0.292 | 0.165 | 0.116 | 49.195 | 0.243 | 0.177 | 0.197 | 0.163 | 0.139 | 0.189 |
| Stanford Narcolepsy samples with primary East Asian Ancestry | P-value | 0.288 | 0.131 | 0.014 | 0.030 | 0.497 | 0.653 | 0.763 | 0.319 | 0.758 | 6.79E-06 | 0.944 | 0.057 | 0.866 |
| | beta | 0.203 | 0.201 | 0.413 | -0.289 | -0.079 | 0.050 | -5.484 | 0.158 | 0.038 | 0.481 | -0.017 | 0.214 | 0.021 |
| | se | 0.191 | 0.133 | 0.170 | 0.133 | 0.116 | 0.111 | 43.614 | 0.158 | 0.124 | 0.108 | 0.242 | 0.113 | 0.125 |
| Han et al., Narcolepsy | P-value | 0.293 | 0.035 | 2.99E-06 | 2.71E-05 | 0.004 | 6.42E-05 | 0.863 | 8.97E-06 | 0.043 | 4.98E-24 | 0.182 | 7.41E-09 | 1.68E-04 |
| | beta | 0.100 | 0.130 | 0.387 | -0.249 | 0.164 | 0.212 | -0.158 | 0.353 | 0.101 | 0.516 | 0.175 | 0.314 | -0.208 |
| | se | 0.095 | 0.061 | 0.083 | 0.060 | 0.057 | 0.053 | 0.918 | 0.080 | 0.050 | 0.052 | 0.131 | 0.055 | 0.056 |
| Japanese Narcolepsy cohort | P-value | 0.316 | 0.004 | 0.489 | 0.787 | 0.192 | 0.018 | NA | 0.028 | 0.020 | 2.31E-12 | 0.465 | 0.990 | 0.018 |
| | beta | 0.145 | -0.333 | 0.086 | -0.028 | 0.122 | 0.197 | NA | 0.245 | 0.215 | 0.575 | 0.146 | 0.001 | -0.237 |
| | se | 0.145 | 0.114 | 0.124 | 0.102 | 0.094 | 0.083 | NA | 0.112 | 0.093 | 0.082 | 0.200 | 0.089 | 0.101 |
| European Narcolepsy Network | P-value | NA | NA | 0.015 | NA | 0.003 | 0.258 | 0.958 | 0.044 | NA | 8.82E-04 | 0.028 | 0.133 | 0.018 |
| | beta | NA | NA | 0.219 | NA | 0.136 | 0.049 | -0.006 | 0.216 | NA | 0.196 | 0.147 | 0.064 | -0.109 |
| | se | NA | NA | 0.090 | NA | 0.045 | 0.043 | 0.109 | 0.107 | NA | 0.059 | 0.067 | 0.042 | 0.046 |
| Stanford primary European ancestry, second collection; samples from the European narcolepsy network | P-value | 0.014 | 0.206 | 0.118 | 0.051 | 0.004 | 0.021 | 0.036 | 0.032 | 0.736 | 1.54E-09 | 0.010 | 0.998 | 0.007 |
| | beta | 0.214 | 0.119 | 0.272 | -0.248 | 0.244 | 0.191 | -0.617 | 0.392 | 0.033 | 0.623 | 0.368 | 0.000 | -0.247 |
| | se | 0.087 | 0.094 | 0.171 | 0.129 | 0.085 | 0.082 | 0.314 | 0.178 | 0.097 | 0.101 | 0.139 | 0.084 | 0.093 |
| Hallmayer et al., 2009 - AFFY 500k | P-value | 0.348 | 0.215 | 0.286 | 0.931 | 0.141 | 0.609 | 0.076 | 0.769 | 0.880 | 0.015 | 0.408 | 0.426 | 0.416 |
| | beta | 0.166 | 0.228 | 0.409 | 0.021 | 0.267 | 0.081 | -0.897 | 0.103 | 0.027 | 0.508 | 0.231 | 0.126 | -0.141 |
| | se | 0.177 | 0.184 | 0.383 | 0.246 | 0.181 | 0.159 | 0.506 | 0.350 | 0.178 | 0.210 | 0.279 | 0.158 | 0.173 |
| Hallmayer et al., 2009 - AFFY 6.0 | P-value | 0.515 | 3.64E-05 | 0.096 | 0.152 | 0.006 | 0.022 | 0.002 | 0.567 | 0.069 | 4.73E-13 | 2.75E-04 | 0.179 | 5.26E-05 |
| | beta | 0.052 | 0.373 | 0.275 | -0.163 | 0.228 | 0.179 | -0.652 | -0.103 | 0.145 | 0.705 | 0.524 | 0.101 | -0.340 |
| | se | 0.080 | 0.090 | 0.165 | 0.114 | 0.082 | 0.078 | 0.206 | 0.181 | 0.079 | 0.097 | 0.144 | 0.075 | 0.084 |

| | | | | | | | | | | | | | | |
|---|---|---|---|---|---|---|---|---|---|---|---|---|---|---|
| **Stanford primary European ancestry, Samples not from European narcolepsy network** | **P-value** | 5.28E-05 | 2.67E-05 | 0.020 | 2.79E-04 | 0.011 | 5.94E-06 | 1.39E-05 | 0.014 | 1.97E-06 | 1.36E-21 | 0.001 | 0.003 | 2.47E-04 |
| | **beta** | 0.172 | 0.195 | 0.193 | -0.225 | 0.109 | 0.189 | -0.534 | 0.236 | 0.192 | 0.467 | 0.198 | 0.119 | -0.171 |
| | **se** | 0.042 | 0.046 | 0.083 | 0.062 | 0.043 | 0.042 | 0.123 | 0.096 | 0.040 | 0.049 | 0.061 | 0.041 | 0.047 |
| **Stanford primary European ancestry, Third collection** | **P-value** | 0.117 | 0.003 | 0.066 | 0.393 | 6.14E-04 | 0.497 | 0.0288122 | 0.874 | 0.012 | 3.60E-04 | 0.273 | 0.784 | 0.055 |
| | **beta** | 0.220 | 0.452 | 0.523 | -0.157 | 0.490 | 0.090 | -0.853 | 0.049 | 0.328 | 0.611 | 0.220 | -0.038 | -0.274 |
| | **se** | 0.140 | 0.156 | 0.285 | 0.185 | 0.144 | 0.132 | 0.415 | 0.311 | 0.131 | 0.173 | 0.200 | 0.138 | 0.144 |

**Only one cohort is part of the primary African ancestry data set. Heterogeneity test not computed.*

**Supplementary Table 2: Variant associations across ethnic groups.** Genome-wide association statistics for independent risk loci and in different ethnic groups. We show meta-analysis statistics across all cohorts and across ethnic groups. CHR, chromosome; rsid, dbSNP variant ID; Position, hg19 coordinate for variants; beta, effect size; se, standard error; We report effect sizes for effect allele. Raw P-values are reported using two-sided fixed effects meta-analysis, or from two- sided logistic regression for each cohort.

| **Supplementary Table 3. Genetic correlation with Narcolepsy and autoimmune traits** | | | | |
|---|---|---|---|---|
| **Trait** | **Genetic correlation (rg)** | **Standard error for genetic correlation (se)** | **Z-Score** | **P-value** |
| Narcolepsy<br>Faraco et al., 2013 | 1.178 | 0.128 | 9.229 | **0.00000000** |
| Systemic lupus erythematosus<br>Bentham et al., 2015 | 0.344 | 0.130 | 2.650 | **8.04E-03** |
| Daytime sleepiness<br>Wang et al., 2019 | 0.175 | 0.054 | 3.272 | **1.07E-03** |
| Daytime sleepiness BMI adjusted<br>Wang et al., 2019 | 0.165 | 0.053 | 3.098 | **1.95E-03** |
| Daytime napping<br>Dashti et al., 2021 | 0.123 | 0.060 | 2.039 | **0.041** |
| Psoriasis<br>Tsoi et al., | 0.275 | 0.130 | 2.109 | **0.03** |
| Celiac disease<br>Dubois et al., 2010 | 0.212 | 0.162 | 1.307 | 0.191 |
| Primary biliary cholangitis<br>Cordell et al., 2015 | 0.137 | 0.122 | 1.126 | 0.260 |
| Ulcerative colitis<br>Anderson et al., 2011 | -0.063 | 0.085 | -0.731 | 0.465 |
| Rheumatoid arthritis<br>Okada et al., 2014 | -0.046 | 0.088 | -0.519 | 0.604 |
| Type-1 diabetes<br>Immunochip | 0.216 | 0.111 | 1.955 | 0.051 |
| Rheumatoid arthritis<br>Immunochip | 0.144 | 0.139 | 1.037 | 0.300 |
| Psoriasis<br>Immunochip | 0.275 | 0.130 | 2.109 | **0.035** |
| Primary biliary cholangitis<br>Immunochip | 0.093 | 0.131 | 0.705 | 0.481 |
| Multiple sclerosis<br>Immunochip | 0.189 | 0.095 | 1.984 | **0.047** |
| Juvenile idiopathic arthritis<br>Immunochip | 0.129 | 0.226 | 0.569 | 0.569 |
| Celiac disease<br>Immunochip | 0.127 | 0.122 | 1.041 | 0.298 |
| *Genetic correlation computed with Narcolepsy sample from primarily European ancestry, excluding the HLA region* | | | | |

**Supplementary Table 3: LD score regression of narcolepsy with other autoimmune traits.** SLE, systemic lupus erythematosus; PBC, primary biliary cholangitis; PBC Liu et al., primary biliary cholangitis from Liu et al.,; CEL, Celiac disease; JIA, Juvenile idiopathic arthritis; CD, Chrohn's disease; IBD, Inflammatory bowel disease; UC, Ulcerative colitis; T1D, Type-1 diabetes; T1D Ichip, type-1 diabetes Immunochip; Pso, Psoriasis; MS, Multiple sclerosis. We used marcolepsy summary stats from multiethnic or from Whites only sub-analysis. Raw P-values are reported using two-sided weighted linear regression.

**Supplementary Table 4. Enrichment of GWAS loci with genes found in previous GWAS**

| GeneSet | N | n | P-value | Adjusted P | Genes |
|---|---|---|---|---|---|
| Asthma | 337 | 7 | 4.22E-09 | 7.66E-06 | *TNFSF4, DENND1B, ZNF365, IKZF4, ERBB3, HLA-DQB1, HLA-DPB1* |
| Alopecia areata | 94 | 5 | 3.62E-08 | 3.28E-05 | *TNFSF4, ZNF365, IKZF4, ERBB3, HLA-DQB1* |
| Type 1 diabetes | 93 | 4 | 6.14E-06 | 3.71E-03 | *IKZF4, ERBB3, CTSH, HLA-DQB1* |
| Primary biliary cholangitis | 105 | 4 | 1.00E-05 | 3.90E-03 | *DENND1B, NAB1, HLA-DQB1, HLA-DPB1* |
| Allergic disease (asthma, hay fever or eczema) | 292 | 5 | 1.07E-05 | 3.90E-03 | *TNFSF4, ZNF365, IKZF4, ERBB3, HLA-DQB1* |
| Eosinophil percentage of granulocytes | 138 | 4 | 3.00E-05 | 9.09E-03 | *ZNF365, IKZF4, HLA-DQB1, HLA-DPB1* |
| Hypothyroidism | 78 | 3 | 4.70E-04 | 1.07E-01 | *ZNF365, ERBB3, HLA-DQB1* |
| Crohn's disease | 630 | 5 | 4.72E-04 | 1.07E-01 | *TNFSF4, DENND1B, ZNF365, IFNAR1, HLA-DQB1* |
| Hepatitis B | 10 | 2 | 7.64E-04 | 1.54E-01 | *HLA-DQB1, HLA-DPB1* |
| Plantar warts | 11 | 2 | 9.33E-04 | 1.65E-01 | *HLA-DQB1, HLA-DPB1* |
| Nodular sclerosis Hodgkin lymphoma | 12 | 2 | 1.12E-03 | 1.65E-01 | *HLA-DQB1, HLA-DPB1* |
| Thyrotoxic hypokalemic periodic paralysis and Graves disease | 12 | 2 | 1.12E-03 | 1.65E-01 | *HLA-DQB1, HLA-DPB1* |
| Systemic lupus erythematosus | 348 | 4 | 1.18E-03 | 1.65E-01 | *TNFSF4, ZNF365, HLA-DQB1, HLA-DPB1* |
| Narcolepsy | 15 | 2 | 1.78E-03 | 2.15E-01 | *ZNF365, CTSH* |
| Myositis | 15 | 2 | 1.78E-03 | 2.15E-01 | *HLA-DQB1, HLA-DPB1* |
| Chronic hepatitis B infection | 16 | 2 | 2.03E-03 | 2.31E-01 | *HLA-DQB1, HLA-DPB1* |
| Eosinophil percentage of white cells | 141 | 3 | 2.78E-03 | 2.97E-01 | *ZNF365, IKZF4, HLA-DPB1* |
| Response to hepatitis B vaccine | 22 | 2 | 3.91E-03 | 3.94E-01 | *HLA-DQB1, HLA-DPB1* |
| Nephropathy | 24 | 2 | 4.67E-03 | 4.40E-01 | *HLA-DQB1, HLA-DPB1* |
| Asthma or allergic disease (pleiotropy) | 170 | 3 | 4.85E-03 | 4.40E-01 | *IKZF4, ERBB3, HLA-DQB1* |
| Sarcoidosis (non-Lofgren's syndrome without extrapulmonary manifestations) | 31 | 2 | 7.85E-03 | 6.79E-01 | *HLA-DQB1, HLA-DPB1* |
| Blood protein levels | 1935 | 6 | 8.99E-03 | 7.42E-01 | *CTSC, CTSH, SIRPG, IFNAR1, HLA-DQB1, HLA-DPB1* |
| IgA nephropathy | 35 | 2 | 1.00E-02 | 7.92E-01 | *HLA-DQB1, HLA-DPB1* |
| Asthma (childhood onset) | 266 | 3 | 1.82E-02 | 1.38E+00 | *TNFSF4, ZNF365, HLA-DQB1* |
| Primary biliary cirrhosis | 48 | 2 | 1.90E-02 | 1.38E+00 | *DENND1B, HLA-DQB1* |
| Sjögren's syndrome | 50 | 2 | 2.06E-02 | 1.44E+00 | *HLA-DQB1, HLA-DPB1* |
| Anorexia nervosa | 58 | 2 | 2.77E-02 | 1.86E+00 | *IKZF4, ERBB3* |
| Systemic sclerosis | 64 | 2 | 3.38E-02 | 2.19E+00 | *HLA-DQB1, HLA-DPB1* |
| Atopic dermatitis | 67 | 2 | 3.70E-02 | 2.32E+00 | *ZNF365, CD207* |
| Cognitive function | 85 | 2 | 5.95E-02 | 3.60E+00 | *IKZF4, ERBB3* |
| Asthma (adult onset) | s | 2 | 8.38E-02 | 4.91E+00 | *ERBB3, HLA-DQB1* |

**Supplementary Table 4: Enrichment of GWAS loci with genes found in previous GWAS.** Results from enrichment analysis from FUMA. N, total number of genes in a gene set; n, number of genes from narcolepsy analysis. Genes reflect those that were overlapping with predefined disease gene set. Raw and adjusted P-values from hypergeometric test are reported.

**Supplementary Table 5. HLA association results in narcolepsy**

| Forward stepwise regression variant set | SNP | P | OR | Heterogeneity p-value | I | P primary Asian Ancestry | OR primary Asian Ancestry | P primary Caucasian Ancestry | OR primary Caucasian Ancestry |
|---|---|---|---|---|---|---|---|---|---|
| DQB1*06:02 adjusted | DQA1 protective | $2.67 \times 10^{-38}$ | 0.37 | 0.16 | 36.97 | $2.18 \times 10^{-10}$ | 0.43 | $1.86 \times 10^{-27}$ | 0.34 |
| | DQA1*01:03 | $8.37 \times 10^{-21}$ | 0.31 | 0.23 | 28.56 | $4.20 \times 10^{-08}$ | 0.37 | $4.09 \times 10^{-13}$ | 0.26 |
| | DQA1*01:01 | $3.15 \times 10^{-16}$ | 0.46 | 0.34 | 11.82 | 0.001 | 0.54 | $1.66 \times 10^{-13}$ | 0.43 |
| DQB1*06:02, DQA1*01 nonDQA1*01:02 adjusted | DQA1*01:02 | $1.88 \times 10^{-07}$ | 1.47 | 0.69 | 0 | 0.039 | 1.36 | $6.07 \times 10^{-05}$ | 1.45 |
| DQB1*06:02 DQA1*01 protective, DQA1*01:02 adjusted | DQB1*03:01 | $5.65 \times 10^{-04}$ | 1.23 | 0.36 | 7.53 | 0.001 | 1.23 | $2.81 \times 10^{-02}$ | 1.24 |
| DQB1*06:02, DQB1*03:01, DQA1*01:02 and DQA1*01 non DQA1*01:02 adjusted | DPB1*04:02 | $2.16 \times 10^{-22}$ | 0.46 | 0.07 | 53.7 | $1.22 \times 10^{-06}$ | 0.44 | $6.37 \times 10^{-19}$ | 0.44 |
| DQB1*06:02, DQB1*03:01, DQA1*01:02 and DQA1*01 non DQA1*01:02 DPB1*04:02 adjusted | DPB1*05:01 | $1.27 \times 10^{-03}$ | 1.23 | 0.33 | 13.06 | $8.36 \times 10^{-03}$ | 1.22 | 0.034 | 1.31 |
| DQB1*06:02, DQB1*03:01, DQA1*01:02 and DQA1*01 non DQA1*01:02, HLA DPB1*04:02, DPB1*05:01 adjusted | A*11:01 | $4.77 \times 10^{-06}$ | 1.35 | 0.32 | 14.37 | $6.48 \times 10^{-05}$ | 1.47 | $6.78 \times 10^{-03}$ | 1.28 |
| | B*51:01 | $8.38 \times 10^{-04}$ | 1.29 | 0.12 | 46.06 | 0.545 | 1.09 | $1.18 \times 10^{-04}$ | 1.41 |
| | B*35:01 | $5.27 \times 10^{-04}$ | 1.31 | 0.19 | 34.37 | 0.334 | 1.14 | $9.47 \times 10^{-04}$ | 1.42 |
| | B*35:03 | 0.0022 | 1.57 | 0.45 | 0 | 0.096 | 2.15 | 0.0075 | 1.51 |
| | A*03:01 | 0.0045 | 0.84 | 0.34 | 11 | 0.187 | 0.79 | 0.0025 | 0.80 |

**Supplementary Table 5: HLA association results in narcolepsy.** Analysis of HLA allele association in narcolepsy. After identifying the main effect as DQB1*06:02 the stepwise forward regression analysis of HLA allele association with narcolepsy is shown with each added allele labelled. We show P-values across ethnic groups (P) and estimate heterogeneity between ancestries with I and heterogeneity P-value. Raw P-values are reported using two-sided fixed effects meta-analysis. Heterogeneity p-values computed from Cochrane Mantel Haenszel test.

**Supplementary Table 6. Enrichment of Immune cell subsets with narcolepsy**

| Comparison | GeneSet Name | N genes in reference data | N observed in narcolepsy GWAS | P-value | Adjusted P-value | Genes |
|---|---|---|---|---|---|---|
| Genes up-regulated in activated CD4 T cells expressing: wildtype versus mutant form of FOXP3 | GSE41087_WT_VS_FOXP3_MUT_ANTI_CD3_CD28_STIM_CD4_TCELL_UP | 196 | 4 | 8.99E-07 | 0.00158 | TNFSF4, NAB1, HLA-DQB1, HLA-DPB1 |
| Genes down-regulated in comparison of unstimulated CD8 T cells at 48 h versus CD8 T cells at 48 h after stimulation with IL12. | GSE15930_STIM_VS_STIM_AND_IL12_48H_CD8_T_CELL_DN | 200 | 4 | 9.75E-07 | 0.00158 | TNFSF4, PRF1, IKZF4, NAB1 |
| Genes down-regulated in comparison of monocytes versus myeloid dendritic cells (mDC). | GSE29618_MONOCYTE_VS_MDC_DN | 200 | 4 | 9.75E-07 | 0.00158 | DENND1B, CD207, HLA-DQB1, HLA-DPB1 |
| Genes down-regulated in comparison of dendritic cells activated in the absense of AhR versus those activated in the presence of VAF347 | GSE10463_CD40L_AND_VA347_VS_CD40L_IN_DC_DN | 176 | 3 | 4.32E-05 | 0.0181 | DENND1B, ERBB3, CTSH |
| Genes up-regulated in induced T reg cultured with IL2 | GSE14415_INDUCED_TREG_VS_FOXP3_KO_INDUCED_TREG_IL2_CULTURE_UP | 190 | 3 | 5.42E-05 | 0.0181 | TNFSF4, PRF1, NAB1 |
| Genes down-regulated in dendritic cells: untreated versus anti-FcgRIIB. | GSE7509_UNSTIM_VS_FCGRIIB_STIM_DC_DN | 191 | 3 | 5.51E-05 | 0.0181 | PRF1, CTSH, HLA-DQB1 |
| Genes down-regulated in comparison of naive B cells versus memory B cells. | GSE12366_NAIVE_VS_MEMORY_BCELL_DN | 193 | 3 | 5.68E-05 | 0.0181 | DENND1B, CTSH, NAB1 |
| Genes down-regulated in comparison of systemic lupus erythematosus CD4 T cells versus systemic lupus erythematosus B cells | GSE10325_LUPUS_CD4_TCELL_VS_LUPUS_BCELL_DN | 194 | 3 | 5.77E-05 | 0.0181 | CTSH, HLA-DQB1, HLA-DPB1 |
| Genes up-regulated in comparison of central memory CD4 T cells versus Th1 cells. | GSE3982_CENT_MEMORY_CD4_TCELL_VS_TH1_UP | 196 | 3 | 5.94E-05 | 0.0181 | DENND1B, SIRPG, HLA-DPB1 |
| Genes up-regulated in comparison of control polymorphonuclear leukocytes (PMN) at 12 h versus PMN treated with F. tularensis vaccine at 24 h. | GSE37416_12H_VS_24H_F_TULARENSIS_LVS_NEUTROPHIL_UP | 198 | 3 | 6.13E-05 | 0.0181 | CTSH, NAB1, IFNAR1 |
| Genes down-regulated in bone marrow-derived macrophages: wildtype versus PPARG knockout | GSE25123_WT_VS_PPARG_KO_MACROPHAGE_DN | 198 | 3 | 6.13E-05 | 0.0181 | IKZF4, HLA-DQB1, HLA-DPB1 |
| Genes down-regulated in skin with IL1R1 knockout: uninfected versus S. aureus infection. | GSE36826_NORMAL_VS_STAPH_AUREUS_INF_IL1R_KO_SKIN_DN | 198 | 3 | 6.13E-05 | 0.0181 | NAB1, HLA-DQB1, HLA-DPB1 |
| Genes up-regulated in comparison of macrophages versus Th1 cells. | GSE3982_MAC_VS_TH1_UP | 198 | 3 | 6.13E-05 | 0.0181 | DENND1B, CTSH, IFNAR1 |
| Genes up-regulated in comparison of directly activated CD4 T cells versus bystander activated CD4 T cells. | GSE13738_TCR_VS_BYSTANDER_ACTIVATED_CD4_TCELL_UP | 198 | 3 | 6.13E-05 | 0.0181 | PRF1, NAB1, SIRPG |
| Genes down-regulated in comparison of microglia cells 1 h after stimulation with IFNG versus microglia cells 24 h after the stimulation. | GSE1432_1H_VS_24H_IFNG_MICROGLIA_DN | 200 | 3 | 6.31E-05 | 0.0181 | DENND1B, HLA-DQB1, HLA-DPB1 |
| Genes down-regulated in monocyte-derived dendritic cells: AM580 versus rosiglitazone and AM580. | GSE5679_RARA_AGONIST_AM580_VS_AM580_AND_ROSIGLITAZONE_TREATED_DC_DN | 200 | 3 | 6.31E-05 | 0.0181 | TNFSF4, IKZF4, IFNAR1 |
| Genes down-regulated in comparison of healthy CD4 T cells versus healthy myeloid cells. | GSE10325_CD4_TCELL_VS_MYELOID_DN | 200 | 3 | 6.31E-05 | 0.0181 | CTSH, HLA-DQB1, HLA-DPB1 |

**Supplementary Table 6: Enrichment of immune cell subsets with narcolepsy.** We provide enrichment of immune cell subsets for the significant loci with number of genes in reference (N genes in reference), number of genes observed in the narcolepsy data (N observed in narcolepsy GWAS) and unadjusted and adjusted P-values. Raw and adjusted two-sided P-values from a hypergeometric test are reported.

**Supplementary Table 7. Association of rs10085999 with T cell repertoire and chain usage**

| SNP | Gene | Gene ID | CNV chromosome | Gene chromosome | SNP location | Gene location | Distance | R-value | P-value | P-value $-\log_{10}$ | Adjusted $r^2$ | Gradient | Gene type | Chain | SNP type | Empiric p-value | SNP rank min | SNP rank max | SNPs per gene |
|---|---|---|---|---|---|---|---|---|---|---|---|---|---|---|---|---|---|---|---|
| rs1008599 | TRBV4-2 | TRBV4-2 | 7 | 7 | 142038782 | 142045251 | 6469 | -0.669 | 0 | 116.4302 | 0.4468 | -1.0457 | TR | TRBV | RS | 0 | 4 | 4 | 5586 |
| rs1008599 | TRBV7-3 | TRBV7-3 | 7 | 7 | 142038782 | 142247108 | 208326 | -0.505 | 0 | 58.4052 | 0.2543 | -0.7897 | TR | TRBV | RS | 0 | 176 | 176 | 5696 |
| rs1008599 | TRBV10-1 | TRBV10-1 | 7 | 7 | 142038782 | 142231572 | 192790 | 0.465 | 0 | 48.4526 | 0.2152 | 0.7267 | TR | TRBV | RS | 0 | 57 | 57 | 5728 |
| rs1008599 | TRBV6-8 | TRBV6-8 | 7 | 7 | 142038782 | 142124136 | 85354 | 0.428 | 0 | 40.3963 | 0.1821 | 0.6687 | TR | TRBV | RS | 0 | 94 | 94 | 5585 |
| rs1008599 | TRBV7-1 | TRBV7-1 | 7 | 7 | 142038782 | 142032005 | 6777 | -0.412 | 0 | 37.2544 | 0.1688 | -0.644 | TR | TRBV | RS | 0 | 51 | 51 | 5552 |
| rs1008599 | TRBV10-2 | TRBV10-2 | 7 | 7 | 142038782 | 142206510 | 167728 | 0.409 | 0 | 36.6119 | 0.166 | 0.6388 | TR | TRBV | RS | 0 | 87 | 87 | 5716 |
| rs1008599 | TRBV5-1 | TRBV5-1 | 7 | 7 | 142038782 | 142020495 | 18287 | 0.383 | 0 | 31.8761 | 0.1455 | 0.5983 | TR | TRBV | RS | 0 | 22 | 22 | 5581 |
| rs1008599 | TRBV3-1 | TRBV3-1 | 7 | 7 | 142038782 | 142008364 | 30418 | 0.365 | 0 | 28.9252 | 0.1325 | 0.5712 | TR | TRBV | RS | 0 | 5 | 5 | 5608 |
| rs1008599 | TRBV6-1 | TRBV6-1 | 7 | 7 | 142038782 | 142028120 | 10662 | 0.347 | 0 | 26.0423 | 0.1196 | 0.5429 | TR | TRBV | RS | 0 | 24 | 24 | 5566 |
| rs1008599 | TRBV2 | TRBV2 | 7 | 7 | 142038782 | 142000746 | 38036 | 0.324 | 0 | 22.5511 | 0.1038 | 0.506 | TR | TRBV | RS | 0 | 7 | 7 | 5625 |
| rs1008599 | TRBV7-6 | TRBV7-6 | 7 | 7 | 142038782 | 142139277 | 100495 | -0.321 | 0 | 22.1503 | 0.1019 | -0.5016 | TR | TRBV | RS | 0 | 90 | 90 | 5588 |
| rs1008599 | TRBV7-7 | TRBV7-7 | 7 | 7 | 142038782 | 142119819 | 81037 | -0.314 | 0 | 21.25 | 0.0978 | -0.4914 | TR | TRBV | RS | 0 | 93 | 93 | 5576 |
| rs1008599 | TRBV7-8 | TRBV7-8 | 7 | 7 | 142038782 | 142099454 | 60672 | -0.29 | 0 | 18.1149 | 0.0833 | -0.4538 | TR | TRBV | RS | 0 | 93 | 93 | 5581 |
| rs1008599 | TRBV11-1 | TRBV11-1 | 7 | 7 | 142038782 | 142223819 | 185037 | -0.213 | 1.10E-10 | 9.9571 | 0.0445 | -0.3337 | TR | TRBV | RS | 0 | 275 | 275 | 5739 |
| rs1008599 | TRBV4-1 | TRBV4-1 | 7 | 7 | 142038782 | 142013006 | 25776 | 0.184 | 3.17E-08 | 7.4985 | 0.0326 | 0.287 | TR | TRBV | RS | 1.00E-04 | 509 | 509 | 5596 |
| rs1008599 | TRBV9 | TRBV9 | 7 | 7 | 142038782 | 142239536 | 200754 | -0.161 | 1.22E-06 | 5.9135 | 0.0249 | -0.2522 | TR | TRBV | RS | 0.0023 | 287 | 287 | 5714 |
| rs1008599 | TRBV1 | TRBV1 | 7 | 7 | 142038782 | 141999016 | 39766 | 0.142 | 2.02E-05 | 4.6941 | 0.0191 | 0.2219 | TR | TRBV | RS | 0.0323 | 141 | 141 | 5625 |
| rs1008599 | TRBV6-6 | TRBV6-6 | 7 | 7 | 142038782 | 142161930 | 123148 | 0.133 | 6.93E-05 | 4.1592 | 0.0165 | 0.2073 | TR | TRBV | RS | 0.1014 | 316 | 316 | 5558 |
| rs1008599 | TRBV5-5 | TRBV5-5 | 7 | 7 | 142038782 | 142148927 | 110145 | 0.114 | 6.6531E-04 | 3.177 | 0.0118 | 0.1775 | TR | TRBV | RS | 0.5809 | 450 | 450 | 5572 |
| rs1008599 | TRBV27 | TRBV27 | 7 | 7 | 142038782 | 142423172 | 384390 | 0.113 | 7.0964E-04 | 3.149 | 0.0117 | 0.1766 | TR | TRBV | RS | 0.5826 | 456 | 456 | 5394 |
| rs1008599 | TRBV5-6 | TRBV5-6 | 7 | 7 | 142038782 | 142131411 | 92629 | 0.086 | 1.0027E-02 | 1.9988 | 0.0063 | 0.1345 | TR | TRBV | RS | 1 | 710 | 710 | 5596 |
| rs1008599 | TRBV7-4 | TRBV7-4 | 7 | 7 | 142038782 | 142176328 | 137546 | 0.079 | 1.7988E-02 | 1.745 | 0.0051 | 0.1236 | TR | TRBV | RS | 1 | 825 | 825 | 5587 |
| rs1008599 | TRBV12-2 | TRBV12-2 | 7 | 7 | 142038782 | 142190637 | 151855 | 0.077 | 2.0727E-02 | 1.6835 | 0.0049 | 0.1208 | TR | TRBV | RS | 1 | 206 | 205 | 5617 |
| rs1008599 | TRBV6-4 | TRBV6-4 | 7 | 7 | 142038782 | 142250702 | 211920 | 0.073 | 2.9324E-02 | 1.5328 | 0.0042 | 0.1139 | TR | TRBV | RS | 1 | 356 | 356 | 5681 |
| rs1008599 | TRBV12-1 | TRBV12-1 | 7 | 7 | 142038782 | 142216289 | 177507 | 0.072 | 3.2445E-02 | 1.4889 | 0.004 | 0.1118 | TR | TRBV | RS | 1 | 1311 | 1311 | 5739 |
| rs1008599 | TRBJ2-7 | TRBJ2-7 | 7 | 7 | 142038782 | 142495139 | 456357 | -0.067 | 4.6243E-02 | 1.335 | 0.0033 | -0.1042 | TR | TRBnonV | RS | 1 | 1269 | 1269 | 5284 |
| rs1008599 | TRBV7-5 | TRBV7-5 | 7 | 7 | 142038782 | 142157377 | 118595 | 0.058 | 8.1240E-02 | 1.0902 | 0.0023 | 0.0912 | TR | TRBV | RS | 1 | 885 | 885 | 5556 |
| rs1008599 | TRBV24-1 | TRBV24-1 | 7 | 7 | 142038782 | 142364197 | 325415 | -0.058 | 8.4783E-02 | 1.0717 | 0.0022 | -0.0901 | TR | TRBV | RS | 1 | 1442 | 1442 | 5516 |
| rs1008599 | TRBV5-4 | TRBV5-4 | 7 | 7 | 142038782 | 142168379 | 129597 | 0.057 | 8.7132E-02 | 1.0598 | 0.0022 | 0.0894 | TR | TRBV | RS | 1 | 1206 | 1206 | 5575 |
| rs1008599 | TRBV20-1 | TRBV20-1 | 7 | 7 | 142038782 | 142334162 | 295380 | 0.053 | 1.1390E-01 | 0.9435 | 0.0017 | 0.0827 | TR | TRBV | RS | 1 | 969 | 968 | 5567 |
| rs1008599 | TRBV28 | TRBV28 | 7 | 7 | 142038782 | 142428483 | 389701 | -0.05 | 1.3376E-01 | 0.8737 | 0.0014 | -0.0784 | TR | TRBV | RS | 1 | 2036 | 2036 | 5402 |
| rs1008599 | TRBJ2-1 | TRBJ2-1 | 7 | 7 | 142038782 | 142494048 | 455266 | -0.05 | 1.3715E-01 | 0.8628 | 0.0014 | -0.0777 | TR | TRBnonV | RS | 1 | 1615 | 1615 | 5284 |
| rs1008599 | TRBC2 | TRBC2 | 7 | 7 | 142038782 | 142498724 | 459942 | -0.044 | 1.8778E-01 | 0.7263 | 8.00E-04 | -0.0689 | TR | TRBnonV | RS | 1 | 2378 | 2378 | 5283 |
| rs1008599 | TRBJ2-3 | TRBJ2-3 | 7 | 7 | 142038782 | 142494530 | 455748 | 0.038 | 2.5844E-01 | 0.5876 | 3.00E-04 | 0.0591 | TR | TRBnonV | RS | 1 | 2645 | 2644 | 5283 |
| rs1008599 | TRBV5-3 | TRBV5-3 | 7 | 7 | 142038782 | 142242280 | 203498 | 0.037 | 2.6819E-01 | 0.5716 | 3.00E-04 | 0.0579 | TR | TRBV | RS | 1 | 2057 | 2057 | 5705 |
| rs1008599 | TRBJ2-2P | TRBJ2-2P | 7 | 7 | 142038782 | 142494380 | 455598 | 0.034 | 3.0557E-01 | 0.5149 | 1.00E-04 | 0.0536 | TR | TRBnonV | RS | 1 | 2858 | 2857 | 5284 |
| rs1008599 | TRBJ2-6 | TRBJ2-6 | 7 | 7 | 142038782 | 142494922 | 456140 | -0.032 | 3.3535E-01 | 0.4745 | -1.00E-04 | -0.0504 | TR | TRBnonV | RS | 1 | 1832 | 1832 | 5284 |
| rs1008599 | TRBV5-7 | TRBV5-7 | 7 | 7 | 142038782 | 142111392 | 72610 | -0.029 | 3.7994E-01 | 0.4203 | -3.00E-04 | -0.0459 | TR | TRBV | RS | 1 | 3119 | 3117 | 5578 |
| rs1008599 | TRBJ2-4 | TRBJ2-4 | 7 | 7 | 142038782 | 142494681 | 455899 | -0.027 | 4.1908E-01 | 0.3777 | -4.00E-04 | -0.0423 | TR | TRBnonV | RS | 1 | 2951 | 2951 | 5283 |
| rs1008599 | TRBV25-1 | TRBV25-1 | 7 | 7 | 142038782 | 142378571 | 339789 | 0.027 | 4.2270E-01 | 0.374 | -4.00E-04 | 0.0419 | TR | TRBV | RS | 1 | 2649 | 2649 | 5460 |
| rs1008599 | TRBV6-5 | TRBV6-5 | 7 | 7 | 142038782 | 142180514 | 141732 | -0.024 | 4.6837E-01 | 0.3294 | -5.00E-04 | -0.0379 | TR | TRBV | RS | 1 | 3379 | 3379 | 5597 |
| rs1008599 | TRBJ2-2 | TRBJ2-2 | 7 | 7 | 142038782 | 142494243 | 455461 | 0.023 | 4.8965E-01 | 0.3101 | -6.00E-04 | 0.0361 | TR | TRBnonV | RS | 1 | 2998 | 2995 | 5284 |
| rs1008599 | TRBV19 | TRBV19 | 7 | 7 | 142038782 | 142326362 | 287580 | 0.021 | 5.3897E-01 | 0.2684 | -7.00E-04 | 0.0321 | TR | TRBV | RS | 1 | 3094 | 3094 | 5578 |
| rs1008599 | TRBV23-1 | TRBV23-1 | 7 | 7 | 142038782 | 142353467 | 314685 | -0.02 | 5.4538E-01 | 0.2633 | -7.00E-04 | -0.0316 | TR | TRBV | RS | 1 | 3044 | 3043 | 5537 |
| rs1008599 | TRBV21-1 | TRBV21-1 | 7 | 7 | 142038782 | 142344426 | 305644 | 0.019 | 5.6058E-01 | 0.2514 | -7.00E-04 | 0.0305 | TR | TRBV | RS | 1 | 3142 | 3142 | 5553 |
| rs1008599 | TRBV29-1 | TRBV29-1 | 7 | 7 | 142038782 | 142448052 | 409270 | 0.019 | 5.7881E-01 | 0.2375 | -8.00E-04 | 0.029 | TR | TRBV | RS | 1 | 3748 | 3747 | 5383 |
| rs1008599 | TRBV30 | TRBV30 | 7 | 7 | 142038782 | 142510270 | 471488 | -0.005 | 8.7840E-01 | 0.0563 | -0.0011 | -0.008 | TR | TRBV | RS | 1 | 4654 | 4654 | 5262 |
| rs1008599 | TRBJ2-5 | TRBJ2-5 | 7 | 7 | 142038782 | 142494802 | 456020 | -0.005 | 8.8470E-01 | 0.0532 | -0.0011 | -0.0076 | TR | TRBnonV | RS | 1 | 4839 | 4839 | 5283 |
| rs1008599 | TRBV6-7 | TRBV6-7 | 7 | 7 | 142038782 | 142143651 | 104869 | -0.001 | 9.7690E-01 | 0.0101 | -0.0011 | -0.0015 | TR | TRBV | RS | 1 | 5497 | 5497 | 5585 |

**Supplementary Table 7: Association of rs10085999 with T cell repertoire and chain usage.** We provide association statistics between rs10085999 and gene chain usage for the following parameters: Distance between variant and gene, R-value, P-value, P-value - log10, Adjusted r2, Gradient, Chain, Empiric p-value, SNP rank, and SNPs per gene. Raw and adjusted two sided P-values from linear regression are reported.

**Supplementary Table 8. Phenotypic analysis of co-occurrence of autoimmune traits and narcolepsy with cataplexy**

| | | Age, Sex, PCs adjusted | | | | | | |
|---|---|---|---|---|---|---|---|---|
| **Autoimmune trait** | **N autoimmune trait** | **Estimate (beta)** | **SE** | **OR** | **P-value** | **Significant** | **CI lower** | **CI upper** |
| DM1 | 8671 | 0.437 | 0.421 | 1.548 | 0.3 | ns | 0.678 | 3.533 |
| Psoriasis | 6995 | 0.828 | 0.388 | 2.289 | 0.0327 | * | 1.070 | 4.896 |
| Rheuma | 9855 | 0.788 | 0.4 | 2.199 | 0.0493 | * | 1.004 | 4.816 |
| J10_Asthma | 32351 | 1.521 | 0.209 | 4.577 | $3.45 \times 10^{-13}$ | * | 3.038 | 6.894 |
| Hypothyroidism | 38624 | 1.531 | 0.336 | 4.623 | $5.19 \times 10^{-06}$ | * | 2.393 | 8.931 |
| Any autoimmune | | 0.725929 | 0.16287 | 2.067 | $8.31 \times 10^{-06}$ | * | 1.502 | 2.844 |
| Chiril Prim | | *Case overlap too low for analysis* | | | | | | |
| MS | | *Case overlap too low for analysis* | | | | | | |
| Alopecia areata | | *Case overlap too low for analysis* | | | | | | |
| Coeliac disease | | *Case overlap too low for analysis* | | | | | | |
| IBD | | *Case overlap too low for analysis* | | | | | | |
| SLE | | *Case overlap too low for analysis* | | | | | | |
| Chrohn's disease | | *Case overlap too low for analysis* | | | | | | |
| Ulcerative collitis | | *Case overlap too low for analysis* | | | | | | |
| Sjogren | | *Case overlap too low for analysis* | | | | | | |

**n narcolepsy = 154 individuals**

**Supplementary Table 8: Phenotypic analysis of co-occurrence of autoimmune traits and narcolepsy with cataplexy.** We analysed the co-occurence of different autoimmune traits and narcolepsy in 154 individuals with age, sex and PCs adjusted. Raw two sided P-values from logistic regression are reported.

**Supplementary Table 9. FinnGen**

| Full Name | Affiliation | E-mail | Role 1 | Role 2 |
|---|---|---|---|---|
| Aarno Palotie | Institute for Molecular Medicine Finland (FIMM), HiLIFE, University of Helsinki, Helsinki, Finland; Broad Institute of MIT and Harvard; Massachusetts General Hospital | aarno.palotie@helsinki.fi | Steering Committee | Steering Committee |
| Mark Daly | Institute for Molecular Medicine Finland (FIMM), HiLIFE, University of Helsinki, Helsinki, Finland; Broad Institute of MIT and Harvard; Massachusetts General Hospital | mark.daly@helsinki.fi | Steering Committee | Steering Committee |
| Bridget Riley-Gills | Abbvie, Chicago, IL, United States | bridget.rileygillis@abbvie.co | Steering Committee | Pharmaceutical companies |
| Howard Jacob | Abbvie, Chicago, IL, United States | howard.jacob@abbvie.com | Steering Committee | Pharmaceutical companies |
| Dirk Paul | Astra Zeneca, Cambridge, United Kingdom | dirk.paul@astrazeneca.com | Steering Committee | Pharmaceutical companies |
| Athena Matakidou | Astra Zeneca, Cambridge, United Kingdom | athena.x.matakidou@gsk.com | Steering Committee | Pharmaceutical companies |
| Adam Platt | Astra Zeneca, Cambridge, United Kingdom | adam.platt@astrazeneca.co | Steering Committee | Pharmaceutical companies |
| Heiko Runz | Biogen, Cambridge, MA, United States | heiko.runz@biogen.com | Steering Committee | Pharmaceutical companies |
| Sally John | Biogen, Cambridge, MA, United States | sally.john@biogen.com | Steering Committee | Pharmaceutical companies |
| George Okafo | Boehringer Ingelheim, Ingelheim am Rhein, Germany | george.okafo@boehringer-ingelheim.com | Steering Committee | Pharmaceutical companies |
| Nathan Lawless | Boehringer Ingelheim, Ingelheim am Rhein, Germany | nathan.lawless@boehringer-ingelheim.com | Steering Committee | Pharmaceutical companies |
| Heli Salminen-Mankonen | Boehringer Ingelheim, Ingelheim am Rhein, Germany | heli.salminen-mankonen@boehringer-ingelheim.com | Steering Committee | Pharmaceutical companies |
| Robert Plenge | Bristol Myers Squibb, New York, NY, United States | robert.plenge@bms.com | Steering Committee | Pharmaceutical companies |
| Joseph Maranville | Bristol Myers Squibb, New York, NY, United States | joseph.maranville@bms.com | Steering Committee | Pharmaceutical companies |
| Mark McCarthy | Genentech, San Francisco, CA, United States | mccarthy.mark@gene.com | Steering Committee | Pharmaceutical companies |
| Julie Hunkapiller | Genentech, San Francisco, CA, United States | hunkapiller.julie@gene.com | Steering Committee | Pharmaceutical companies |
| Margaret G. Ehm | GlaxoSmithKline, Collegeville, PA, United States | meg.g.ehm@gsk.com | Steering Committee | Pharmaceutical companies |
| Kirsi Auro | GlaxoSmithKline, Espoo, Finland | kirsi.m.auro@gsk.com | Steering Committee | Pharmaceutical companies |
| Simonne Longerich | Merck, Kenilworth, NJ, United States | simonne.longerich@merck.com | Steering Committee | Pharmaceutical companies |
| Caroline Fox | Merck, Kenilworth, NJ, United States | caroline.fox@merck.com | Steering Committee | Pharmaceutical companies |
| Anders Mälarstig | Pfizer, New York, NY, United States | anders.malarstig@pfizer.com | Steering Committee | Pharmaceutical companies |
| Katherine Klinger | Translational Sciences, Sanofi R&D, Framingham, MA, USA | katherine.klinger@sanofi.com | Steering Committee | Pharmaceutical companies |
| Deepak Raipal | Translational Sciences, Sanofi R&D, Framingham, MA, USA | deepak.rajpal@sanofi.com | Steering Committee | Pharmaceutical companies |
| Eric Green | Maze Therapeutics, San Francisco, CA, United States | egreen@mazetx.com | Steering Committee | Pharmaceutical companies |
| Robert Graham | Maze Therapeutics, San Francisco, CA, United States | rgraham@mazetx.com | Steering Committee | Pharmaceutical companies |
| Robert Yang | Janssen Biotech, Beerse, Belgium | ryang31@its.jnj.com | Steering Committee | Pharmaceutical companies |
| Chris O´Donnell | Novartis Institutes for BioMedical Research, Cambridge, MA, United States | chris.odonnell@novartis.com | Steering Committee | Pharmaceutical companies |
| Tomi P. Mäkelä | HiLIFE, University of Helsinki, Finland, Finland | tomi.makela@helsinki.fi | Steering Committee | University of Helsinki & Biobanks |
| Jaakko Kaprio | Institute for Molecular Medicine Finland (FIMM), HiLIFE, University of Helsinki, Finland | jaakko.kaprio@helsinki.fi | Steering Committee | University of Helsinki & Biobanks |
| Petri Virolainen | Auria Biobank / University of Turku / Hospital District of Southwest Finland, Turku, Finland | petri.virolainen@tyks.fi | Steering Committee | University of Helsinki & Biobanks |
| Antti Hakanen | Auria Biobank / University of Turku / Hospital District of Southwest Finland, Turku, Finland | antti.hakanen@tyks.fi | Steering Committee | University of Helsinki & Biobanks |
| Terhi Kilpi | THL Biobank / Finnish Institute for Health and Welfare (THL), Helsinki, Finland | terhi.kilpi@thl.fi | Steering Committee | University of Helsinki & Biobanks |
| Markus Perola | THL Biobank / Finnish Institute for Health and Welfare (THL), Helsinki, Finland | markus.perola@thl.fi | Steering Committee | University of Helsinki & Biobanks |
| Jukka Partanen | Finnish Red Cross Blood Service / Finnish Hematology Registry and Clinical Biobank, Helsinki, Finland | jukka.partanen@veripalvelu.fi | Steering Committee | University of Helsinki & Biobanks |
| Anne Pitkäranta | Helsinki Biobank / Helsinki University and Hospital District of Helsinki and Uusimaa, Helsinki | anne.pitkaranta@hus.fi | Steering Committee | University of Helsinki & Biobanks |
| Taneli Raivio | Helsinki Biobank / Helsinki University and Hospital District of Helsinki and Uusimaa, Helsinki | taneli.raivio@hus.fi | Steering Committee | University of Helsinki & Biobanks |
| Raisa Serpi | Northern Finland Biobank Borealis / University of Oulu / Northern Ostrobothnia Hospital District, Oulu, Finland | raisa.serpi@ppshp.fi | Steering Committee | University of Helsinki & Biobanks |
| Tarja Laitinen | Finnish Clinical Biobank Tampere / University of Tampere / Pirkanmaa Hospital District, Tampere, Finland | tarja.laitinen@pshp.fi | Steering Committee | University of Helsinki & Biobanks |
| Veli-Matti Kosma | Biobank of Eastern Finland / University of Eastern Finland / Northern Savo Hospital District, Kuopio, Finland | veli-matti.kosma@uef.fi | Steering Committee | University of Helsinki & Biobanks |
| Jari Laukkanen | Central Finland Biobank / University of Jyväskylä / Central Finland Health Care District, Jyväskylä, Finland | jari.laukkanen@ksshp.fi | Steering Committee | University of Helsinki & Biobanks |
| Marco Hautalahti | FINBB - Finnish biobank cooperative | marco.hautalahti@finbb.fi | Steering Committee | University of Helsinki & Biobanks |
| Outi Tuovila | Business Finland, Helsinki, Finland | outi.tuovila@businessfinland.fi | Steering Committee | Other Experts/ Non-Voting Members |
| Raimo Pakkanen | Business Finland, Helsinki, Finland | raimo.pakkanen@businessfinland.fi | Steering Committee | Other Experts/ Non-Voting Members |
| Jeffrey Waring | Abbvie, Chicago, IL, United States | jeff.waring@abbvie.com | Scientific | Pharmaceutical companies |
| Bridget Riley-Gillis | Abbvie, Chicago, IL, United States | bridget.rileygillis@abbvie.co | Scientific | Pharmaceutical companies |
| Fedik Rahimov | Abbvie, Chicago, IL, United States | fedik.rahimov@abbvie.com | Scientific | Pharmaceutical companies |
| Ioanna Tachmazidou | Astra Zeneca, Cambridge, United Kingdom | ioanna.tachmazidou@astrazeneca.com | Scientific Committee | Pharmaceutical companies |
| Chia-Yen Chen | Biogen, Cambridge, MA, United States | chiayen.chen@biogen.com | Scientific | Pharmaceutical companies |
| Heiko Runz | Biogen, Cambridge, MA, United States | heiko.runz@biogen.com | Scientific | Pharmaceutical companies |
| Zhihao Ding | Boehringer Ingelheim, Ingelheim am Rhein, Germany | zhihao.ding@boehringer-ingelheim.com | Scientific Committee | Pharmaceutical companies |
| Marc Jung | Boehringer Ingelheim, Ingelheim am Rhein, Germany | marc_oliver.jung@boehringer-ingelheim.com | Scientific Committee | Pharmaceutical companies |
| Shameek Biswas | Bristol Myers Squibb, New York, NY, United States | Shameek.Biswas@bms.com | Scientific | Pharmaceutical companies |
| Rion Pendergrass | Genentech, San Francisco, CA, United States | penders2@gene.com | Scientific | Pharmaceutical companies |
| Julie Hunkapiller | Genentech, San Francisco, CA, United States | hunkapiller.julie@gene.com | Scientific | Pharmaceutical companies |
| Margaret G. Ehm | GlaxoSmithKline, Collegeville, PA, United States | meg.g.ehm@gsk.com | Scientific | Pharmaceutical companies |
| David Pulford | GlaxoSmithKline, Stevenage, United Kingdom | david.x.pulford@gsk.com | Scientific | Pharmaceutical companies |
| Neha Raghavan | Merck, Kenilworth, NJ, United States | neha.raghavan@merck.com | Scientific | Pharmaceutical companies |
| Adriana Huertas-Vazquez | Merck, Kenilworth, NJ, United States | adriana.huertas.vazquez@merck.com | Scientific Committee | Pharmaceutical companies |

| | | | | |
|---|---|---|---|---|
| Jae-Hoon Sul | Merck, Kenilworth, NJ, United States | jae.hoon.sul@merck.com | Scientific | Pharmaceutical companies |
| Anders Mälarstig | Pfizer, New York, NY, United States | anders.malarstig@pfizer.com | Scientific | Pharmaceutical companies |
| Xinli Hu | Pfizer, New York, NY, United States | xinli.hu@pfizer.com | Scientific | Pharmaceutical companies |
| Katherine Klinger | Translational Sciences, Sanofi R&D, Framingham, MA, USA | katherine.klinger@sanofi.com | Scientific | Pharmaceutical companies |
| Robert Graham | Maze Therapeutics, San Francisco, CA, United States | rgraham@mazetx.com | Scientific | Pharmaceutical companies |
| Eric Green | Maze Therapeutics, San Francisco, CA, United States | egreen@mazetx.com | Scientific | Pharmaceutical companies |
| Sahar Mozaffari | Maze Therapeutics, San Francisco, CA, United States | smozaffari@mazetx.com | Scientific | Pharmaceutical companies |
| Dawn Waterworth | Janssen Research & Development, LLC, Spring House, PA, United States | dwaterwo@its.jnj.com | Scientific Committee | Pharmaceutical companies |
| Nicole Renaud | Novartis Institutes for BioMedical Research, Cambridge, MA, United States | nicole.renaud@novartis.com | Scientific Committee | Pharmaceutical companies |
| Ma´en Obeidat | Novartis Institutes for BioMedical Research, Cambridge, MA, United States | maen.obeidat@novartis.com | Scientific Committee | Pharmaceutical companies |
| Samuli Ripatti | Institute for Molecular Medicine Finland (FIMM), HiLIFE, University of Helsinki, Helsinki, Finland | samuli.ripatti@helsinki.fi | Scientific Committee | University of Helsinki & Biobanks |
| Johanna Schleutker | Auria Biobank / Univ. of Turku / Hospital District of Southwest Finland, Turku, Finland | johanna.schleutker@utu.fi | Scientific Committee | University of Helsinki & Biobanks |
| Markus Perola | THL Biobank / Finnish Institute for Health and Welfare (THL), Helsinki, Finland | markus.perola@thl.fi | Scientific Committee | University of Helsinki & Biobanks |
| Mikko Arvas | Finnish Red Cross Blood Service / Finnish Hematology Registry and Clinical Biobank, Helsinki, Finland | mikko.arvas@veripalvelu.fi | Scientific Committee | University of Helsinki & Biobanks |
| Olli Carpén | Helsinki Biobank / Helsinki University and Hospital District of Helsinki and Uusimaa, Helsinki | olli.carpen@helsinki.fi | Scientific Committee | University of Helsinki & Biobanks |
| Reetta Hinttala | Northern Finland Biobank Borealis / University of Oulu / Northern Ostrobothnia Hospital District, Oulu, Finland | reetta.hinttala@oulu.fi | Scientific Committee | University of Helsinki & Biobanks |
| Johannes Kettunen | Northern Finland Biobank Borealis / University of Oulu / Northern Ostrobothnia Hospital District, Oulu, Finland | johannes.kettunen@oulu.fi | Scientific Committee | University of Helsinki & Biobanks |
| Arto Mannermaa | Biobank of Eastern Finland / University of Eastern Finland / Northern Savo Hospital District, Kuopio, Finland | arto.mannermaa@uef.fi | Scientific Committee | University of Helsinki & Biobanks |
| Katriina Aalto-Setälä | Faculty of Medicine and Health Technology, Tampere University, Tampere, Finland | katriina.aalto-setala@tuni.fi | Scientific Committee | University of Helsinki & Biobanks |
| Mika Kähönen | Finnish Clinical Biobank Tampere / University of Tampere / Pirkanmaa Hospital District, Tampere, Finland | mika.kahonen@uta.fi | Scientific Committee | University of Helsinki & Biobanks |
| Jari Laukkanen | Central Finland Biobank / University of Jyväskylä / Central Finland Health Care District, Jyväskylä, Finland | jari.laukkanen@ksshp.fi | Scientific Committee | University of Helsinki & Biobanks |
| Johanna Mäkelä | FINBB - Finnish biobank cooperative | johanna.makela@finbb.fi | Scientific Committee | University of Helsinki & Biobanks |
| Reetta Kälviäinen | Northern Savo Hospital District, Kuopio, Finland | reetta.kalviainen@kuh.fi | Clinical Groups | Neurology Group |
| Valtteri Julkunen | Northern Savo Hospital District, Kuopio, Finland | valtteri.julkunen@kuh.fi | Clinical Groups | Neurology Group |
| Hilkka Soininen | Northern Savo Hospital District, Kuopio, Finland | hilkka.soininen@uef.fi | Clinical Groups | Neurology Group |
| Anne Remes | Northern Ostrobothnia Hospital District, Oulu, Finland | anne.remes@oulu.fi | Clinical Groups | Neurology Group |
| Mikko Hiltunen | University of Eastern Finland, Kuopio, Finland | mikko.hiltunen@uef.fi | Clinical Groups | Neurology Group |
| Jukka Peltola | Pirkanmaa Hospital District, Tampere, Finland | jukka.peltola@pshp.fi | Clinical Groups | Neurology Group |
| Minna Raivio | Hospital District of Helsinki and Uusimaa, Helsinki, Finland | minna.raivio@geri.fi | Clinical Groups | Neurology Group |
| Pentti Tienari | Hospital District of Helsinki and Uusimaa, Helsinki, Finland | pentti.tienari@hus.fi | Clinical Groups | Neurology Group |
| Juha Rinne | Hospital District of Southwest Finland, Turku, Finland | juha.rinne@tyks.fi | Clinical Groups | Neurology Group |
| Roosa Kallionpää | Hospital District of Southwest Finland, Turku, Finland | roosa.kallionpaa@tyks.fi | Clinical Groups | Neurology Group |
| Juulia Partanen | Institute for Molecular Medicine Finland, HiLIFE, University of Helsinki, Finland | juulia.partanen@helsinki.fi | Clinical Groups | Neurology Group |
| Ali Abbasi | Abbvie, Chicago, IL, United States | ali.abbasi@abbvie.com | Clinical Groups | Neurology Group |
| Adam Ziemann | Abbvie, Chicago, IL, United States | adam.ziemann@abbvie.com | Clinical Groups | Neurology Group |
| Nizar Smaoui | Abbvie, Chicago, IL, United States | nizar.smaoui@abbvie.com | Clinical Groups | Neurology Group |
| Anne Lehtonen | Abbvie, Chicago, IL, United States | anne.lehtonen@abbvie.com | Clinical Groups | Neurology Group |
| Susan Eaton | Biogen, Cambridge, MA, United States | susan.eaton@biogen.com | Clinical Groups | Neurology Group |
| Heiko Runz | Biogen, Cambridge, MA, United States | heiko.runz@biogen.com | Clinical Groups | Neurology Group |
| Sanni Lahdenperä | Biogen, Cambridge, MA, United States | sanni.lahdenpera@biogen.co m | Clinical Groups | Neurology Group |
| Shameek Biswas | Bristol Myers Squibb, New York, NY, United States | shameek.biswas@bms.com | Clinical Groups | Neurology Group |
| Julie Hunkapiller | Genentech, San Francisco, CA, United States | hunkapiller.julie@gene.com | Clinical Groups | Neurology Group |
| Natalie Bowers | Genentech, San Francisco, CA, United States | bowersn1@gene.com | Clinical Groups | Neurology Group |
| Edmond Teng | Genentech, San Francisco, CA, United States | teng.edmond@gene.com | Clinical Groups | Neurology Group |
| Rion Pendergrass | Genentech, San Francisco, CA, United States | penders2@gene.com | Clinical Groups | Neurology Group |
| Fanli Xu | GlaxoSmithKline, Brentford, United Kingdom | chun-fang.2.xu@gsk.com | Clinical Groups | Neurology Group |
| David Pulford | GlaxoSmithKline, Stevenage, United Kingdom | david.x.pulford@gsk.com | Clinical Groups | Neurology Group |
| Kirsi Auro | GlaxoSmithKline, Espoo, Finland | kirsi.m.auro@gsk.com | Clinical Groups | Neurology Group |
| Laura Addis | GlaxoSmithKline, Brentford, United Kingdom | laura.x.addis@gsk.com | Clinical Groups | Neurology Group |
| John Eicher | GlaxoSmithKline, Brentford, United Kingdom | john.d.eicher@gsk.com | Clinical Groups | Neurology Group |
| Qingqin S Li | Janssen Research & Development, LLC, Titusville, NJ 08560, United States | QLi2@its.jnj.com | Clinical Groups | Neurology Group |
| Karen He | Janssen Research & Development, LLC, Spring House, PA, United States | khe2@its.jnj.com | Clinical Groups | Neurology Group |
| Ekaterina Khramtsova | Janssen Research & Development, LLC, Spring House, PA, United States | ekhramts@its.jnj.com | Clinical Groups | Neurology Group |
| Neha Raghavan | Merck, Kenilworth, NJ, United States | neha.raghavan@merck.com | Clinical Groups | Neurology Group |
| Martti Färkkilä | Hospital District of Helsinki and Uusimaa, Helsinki, Finland | martti.farkkila@hus.fi | Clinical Groups | Gastroenterology Group |
| Jukka Koskela | Hospital District of Helsinki and Uusimaa, Helsinki, Finland | jukka.koskela@helsinki.fi | Clinical Groups | Gastroenterology Group |
| Sampsa Pikkarainen | Hospital District of Helsinki and Uusimaa, Helsinki, Finland | sampsa.pikkarainen@hus.fi | Clinical Groups | Gastroenterology Group |
| Airi Jussila | Pirkanmaa Hospital District, Tampere, Finland | airi.jussila@pshp.fi | Clinical Groups | Gastroenterology Group |
| Katri Kaukinen | Pirkanmaa Hospital District, Tampere, Finland | katri.kaukinen@tuni.fi | Clinical Groups | Gastroenterology Group |
| Timo Blomster | Northern Ostrobothnia Hospital District, Oulu, Finland | timo.blomster@ppshp.fi | Clinical Groups | Gastroenterology Group |
| Mikko Kiviniemi | Northern Savo Hospital District, Kuopio, Finland | mikko.kiviniemi@kuh.fi | Clinical Groups | Gastroenterology Group |
| Markku Voutilainen | Hospital District of Southwest Finland, Turku, Finland | markku.voutilainen@tyks.fi | Clinical Groups | Gastroenterology Group |
| Mark Daly | Institute for Molecular Medicine, Finland (FIMM), HiLIFE, University of Helsinki, Helsinki, Finland; Broad Institute of MIT and Harvard; Massachusetts General Hospital | mark.daly@helsinki.fi | Clinical Groups | Gastroenterology Group |
| Ali Abbasi | Abbvie, Chicago, IL, United States | ali.abbasi@abbvie.com | Clinical Groups | Gastroenterology Group |
| Jeffrey Waring | Abbvie, Chicago, IL, United States | jeff.waring@abbvie.com | Clinical Groups | Gastroenterology Group |
| Nizar Smaoui | Abbvie, Chicago, IL, United States | nizar.smaoui@abbvie.com | Clinical Groups | Gastroenterology Group |
| Fedik Rahimov | Abbvie, Chicago, IL, United States | fedik.rahimov@abbvie.com | Clinical Groups | Gastroenterology Group |
| Anne Lehtonen | Abbvie, Chicago, IL, United States | anne.lehtonen@abbvie.com | Clinical Groups | Gastroenterology Group |
| Tim Lu | Genentech, San Francisco, CA, United States | lut8@gene.com | Clinical Groups | Gastroenterology Group |

| | | | | |
|---|---|---|---|---|
| Natalie Bowers | Genentech, San Francisco, CA, United States | bowersn1@gene.com | Clinical Groups | Gastroenterology Group |
| Rion Pendergrass | Genentech, San Francisco, CA, United States | penders2@gene.com | Clinical Groups | Gastroenterology Group |
| Linda McCarthy | GlaxoSmithKline, Brentford, United Kingdom | linda.c.mccarthy@gsk.com | Clinical Groups | Gastroenterology Group |
| Amy Hart | Janssen Research & Development, LLC, Spring House, PA, United States | ahart13@its.jnj.com | Clinical Groups | Gastroenterology Group |
| Meijian Guan | Janssen Research & Development, LLC, Spring House, PA, United States | mguan4@its.jnj.com | Clinical Groups | Gastroenterology Group |
| Jason Miller | Merck, Kenilworth, NJ, United States | jason.miller4@merck.com | Clinical Groups | Gastroenterology Group |
| Kirsi Kalpala | Pfizer, New York, NY, United States | kirsi.kalpala@pfizer.com | Clinical Groups | Gastroenterology Group |
| Melissa Miller | Pfizer, New York, NY, United States | melissa.r.miller@pfizer.com | Clinical Groups | Gastroenterology Group |
| Xinli Hu | Pfizer, New York, NY, United States | xinli.hu@pfizer.com | Clinical Groups | Gastroenterology Group |
| Kari Eklund | Hospital District of Helsinki and Uusimaa, Helsinki, Finland | kari.eklund@hus.fi | Clinical Groups | Rheumatology Group |
| Antti Palomäki | Hospital District of Southwest Finland, Turku, Finland | ajpalo@utu.fi | Clinical Groups | Rheumatology Group |
| Pia Isomäki | Pirkanmaa Hospital District, Tampere, Finland | pia.isomaki@pshp.fi | Clinical Groups | Rheumatology Group |
| Laura Pirilä | Hospital District of Southwest Finland, Turku, Finland | laura.pirila@fimnet.fi,laura.pirila@tyks.fi | Clinical Groups | Rheumatology Group |
| Oili Kaipiainen-Seppänen | Northern Savo Hospital District, Kuopio, Finland | oili.kaipiainen-seppanen@kuh.fi | Clinical Groups | Rheumatology Group |
| Johanna Huhtakangas | Northern Ostrobothnia Hospital District, Oulu, Finland | johanna.huhtakangas@kuh.fi | Clinical Groups | Rheumatology Group |
| Nina Mars | Institute for Molecular Medicine Finland (FIMM), HiLIFE, University of Helsinki, Helsinki, Finland | nina.mars@helsinki.fi | Clinical Groups | Rheumatology Group |
| Ali Abbasi | Abbvie, Chicago, IL, United States | ali.abbasi@abbvie.com | Clinical Groups | Rheumatology Group |
| Jeffrey Waring | Abbvie, Chicago, IL, United States | jeff.waring@abbvie.com | Clinical Groups | Rheumatology Group |
| Fedik Rahimov | Abbvie, Chicago, IL, United States | fedik.rahimov@abbvie.com | Clinical Groups | Rheumatology Group |
| Apinya Lertratanakul | Abbvie, Chicago, IL, United States | apinya.lertratanakul@abbvie.com | Clinical Groups | Rheumatology Group |
| Nizar Smaoui | Abbvie, Chicago, IL, United States | nizar.smaoui@abbvie.com | Clinical Groups | Rheumatology Group |
| Anne Lehtonen | Abbvie, Chicago, IL, United States | anne.lehtonen@abbvie.com | Clinical Groups | Rheumatology Group |
| Marla Hochfeld | Bristol Myers Squibb, New York, NY, United States | mhochfeld@celgene.com | Clinical Groups | Rheumatology Group |
| Natalie Bowers | Genentech, San Francisco, CA, United States | bowersn1@gene.com | Clinical Groups | Rheumatology Group |
| Rion Pendergrass | Genentech, San Francisco, CA, United States | penders2@gene.com | Clinical Groups | Rheumatology Group |
| Jorge Esparza Gordillo | GlaxoSmithKline, Brentford, United Kingdom | jorge.x.esparza-gordillo@gsk.com | Clinical Groups | Rheumatology Group |
| Kirsi Auro | GlaxoSmithKline, Espoo, Finland | kirsi.m.auro@gsk.com | Clinical Groups | Rheumatology Group |
| Dawn Waterworth | Janssen Research & Development, LLC, Spring House, PA, United States | dwaterwo@its.jnj.com | Clinical Groups | Rheumatology Group |
| Fabiana Farias | Merck, Kenilworth, NJ, United States | fabiana.farias@merck.com | Clinical Groups | Rheumatology Group |
| Kirsi Kalpala | Pfizer, New York, NY, United States | kirsi.kalpala@pfizer.com | Clinical Groups | Rheumatology Group |
| Nan Bing | Pfizer, New York, NY, United States | nan.bing@pfizer.com | Clinical Groups | Rheumatology Group |
| Xinli Hu | Pfizer, New York, NY, United States | xinli.hu@pfizer.com | Clinical Groups | Rheumatology Group |
| Tarja Laitinen | Pirkanmaa Hospital District, Tampere, Finland | tarja.laitinen@pshp.fi | Clinical Groups | Pulmonology Group |
| Margit Pelkonen | Northern Savo Hospital District, Kuopio, Finland | margit.pelkonen@kuh.fi | Clinical Groups | Pulmonology Group |
| Paula Kauppi | Hospital District of Helsinki and Uusimaa, Helsinki, Finland | paula.kauppi@hus.fi | Clinical Groups | Pulmonology Group |
| Hannu Kankaanranta | University of Gothenburg, Gothenburg, Sweden/ Seinäjoki Central Hospital, Seinäjoki, Finland/ Tampere University, Tampere, Finland | hannu.kankaanranta@tuni.fi | Clinical Groups | Pulmonology Group |
| Terttu Harju | Northern Ostrobothnia Hospital District, Oulu, Finland | terttu.harju@oulu.fi | Clinical Groups | Pulmonology Group |
| Riitta Lahesmaa | Hospital District of Southwest Finland, Turku, Finland | rilahes@utu.fi | Clinical Groups | Pulmonology Group |
| Nizar Smaoui | Abbvie, Chicago, IL, United States | nizar.smaoui@abbvie.com | Clinical Groups | Pulmonology Group |
| Glenda Lassi | Astra Zeneca, Cambridge, United Kingdom | glenda.lassi@astrazeneca.com | Clinical Groups | Pulmonology Group |
| Susan Eaton | Biogen, Cambridge, MA, United States | susan.eaton@biogen.com | Clinical Groups | Pulmonology Group |
| Hubert Chen | Genentech, San Francisco, CA, United States | chenh37@gene.com | Clinical Groups | Pulmonology Group |
| Rion Pendergrass | Genentech, San Francisco, CA, United States | penders2@gene.com | Clinical Groups | Pulmonology Group |
| Natalie Bowers | Genentech, San Francisco, CA, United States | bowersn1@gene.com | Clinical Groups | Pulmonology Group |
| Joanna Betts | GlaxoSmithKline, Brentford, United Kingdom | joanna.c.betts@gsk.com | Clinical Groups | Pulmonology Group |
| Kirsi Auro | GlaxoSmithKline, Espoo, Finland | kirsi.m.auro@gsk.com | Clinical Groups | Pulmonology Group |
| Rajashree Mishra | GlaxoSmithKline, Brentford, United Kingdom | rajashree.x.mishra@gsk.com | Clinical Groups | Pulmonology Group |
| Majd Mouded | Novartis, Basel, Switzerland | majd.mouded@novartis.com | Clinical Groups | Pulmonology Group |
| Debby Ngo | Novartis, Basel, Switzerland | debby.ngo@novartis.com | Clinical Groups | Pulmonology Group |
| Teemu Niiranen | Finnish Institute for Health and Welfare (THL), Helsinki, Finland | teemu.niiranen@thl.fi | Clinical Groups | Cardiometabolic Diseases Group |
| Felix Vaura | Finnish Institute for Health and Welfare (THL), Helsinki, Finland | fechva@utu.fi | Clinical Groups | Cardiometabolic Diseases Group |
| Veikko Salomaa | Finnish Institute for Health and Welfare (THL), Helsinki, Finland | veikko.salomaa@thl.fi | Clinical Groups | Cardiometabolic Diseases Group |
| Kaj Metsärinne | Hospital District of Southwest Finland, Turku, Finland | kaj.metsarinne@tyks.fi | Clinical Groups | Cardiometabolic Diseases Group |
| Jenni Aittokallio | Hospital District of Southwest Finland, Turku, Finland | jemato@utu.fi | Clinical Groups | Cardiometabolic Diseases Group |
| Mika Kähönen | Pirkanmaa Hospital District, Tampere, Finland | mika.kahonen@uta.fi | Clinical Groups | Cardiometabolic Diseases Group |
| Jussi Hernesniemi | Pirkanmaa Hospital District, Tampere, Finland | jussi.hernesniemi@tuni.fi | Clinical Groups | Cardiometabolic Diseases Group |
| Daniel Gordin | Hospital District of Helsinki and Uusimaa, Helsinki, Finland | daniel.gordin@hus.fi | Clinical Groups | Cardiometabolic Diseases Group |
| Juha Sinisalo | Hospital District of Helsinki and Uusimaa, Helsinki, Finland | juha.sinisalo@hus.fi | Clinical Groups | Cardiometabolic Diseases Group |
| Marja-Riitta Taskinen | Hospital District of Helsinki and Uusimaa, Helsinki, Finland | marja-riitta.taskinen@helsinki.fi | Clinical Groups | Cardiometabolic Diseases Group |
| Tiinamaija Tuomi | Hospital District of Helsinki and Uusimaa, Helsinki, Finland | tiinamaija.tuomi@hus.fi | Clinical Groups | Cardiometabolic Diseases Group |
| Timo Hiltunen | Hospital District of Helsinki and Uusimaa, Helsinki, Finland | timo.hiltunen@hus.fi | Clinical Groups | Cardiometabolic Diseases Group |
| Jari Laukkanen | Central Finland Health Care District, Jyväskylä, Finland | jari.laukkanen@ksshp.fi | Clinical Groups | Cardiometabolic Diseases Group |
| Amanda Elliott | Institute for Molecular Medicine Finland (FIMM), HiLIFE, University of Helsinki, Helsinki, Finland; Broad Institute, Cambridge, MA, USA and Massachusetts General Hospital, Boston, MA, USA | aelliott@broadinstitute.org | Clinical Groups | Cardiometabolic Diseases Group |
| Mary Pat Reeve | Institute for Molecular Medicine Finland (FIMM), HiLIFE, University of Helsinki, Helsinki, Finland | mary.reeve@helsinki.fi | Clinical Groups | Cardiometabolic Diseases Group |

| | | | | |
|---|---|---|---|---|
| Sanni Ruotsalainen | Institute for Molecular Medicine Finland (FIMM), HiLIFE, University of Helsinki, Helsinki, Finland | sanni.ruotsalainen@helsinki.fi | Clinical Groups | Cardiometabolic Diseases Group |
| Benjamin Challis | Astra Zeneca, Cambridge, United Kingdom | benjamin.challis@astrazeneca.com | Clinical Groups | Cardiometabolic Diseases Group |
| Dirk Paul | Astra Zeneca, Cambridge, United Kingdom | dirk.paul@astrazeneca.com | Clinical Groups | Cardiometabolic Diseases Group |
| Julie Hunkapiller | Genentech, San Francisco, CA, United States | hunkapiller.julie@gene.com | Clinical Groups | Cardiometabolic Diseases Group |
| Natalie Bowers | Genentech, San Francisco, CA, United States | bowersn1@gene.com | Clinical Groups | Cardiometabolic Diseases Group |
| Rion Pendergrass | Genentech, San Francisco, CA, United States | penders2@gene.com | Clinical Groups | Cardiometabolic Diseases Group |
| Audrey Chu | GlaxoSmithKline, Brentford, United Kingdom | audrey.y.chu@gsk.com | Clinical Groups | Cardiometabolic Diseases Group |
| Kirsi Auro | GlaxoSmithKline, Espoo, Finland | kirsi.m.auro@gsk.com | Clinical Groups | Cardiometabolic Diseases Group |
| Dermot Reilly | Janssen Research & Development, LLC, Boston, MA, United States | dreill11@its.jnj.com | Clinical Groups | Cardiometabolic Diseases Group |
| Mike Mendelson | Novartis, Boston, MA, United States | mike.mendelson@novartis.com | Clinical Groups | Cardiometabolic Diseases Group |
| Jaakko Parkkinen | Pfizer, New York, NY, United States | jaakko.parkkinen@pfizer.com | Clinical Groups | Cardiometabolic Diseases Group |
| Melissa Miller | Pfizer, New York, NY, United States | melissa.r.miller@pfizer.com | Clinical Groups | Cardiometabolic Diseases Group |
| Tuomo Meretoja | Hospital District of Helsinki and Uusimaa, Helsinki, Finland | tuomo.meretoja@hus.fi | Clinical Groups | Oncology Group |
| Heikki Joensuu | Hospital District of Helsinki and Uusimaa, Helsinki, Finland | heikki.joensuu@hus.fi | Clinical Groups | Oncology Group |
| Olli Carpén | Hospital District of Helsinki and Uusimaa, Helsinki, Finland | olli.carpen@helsinki.fi | Clinical Groups | Oncology Group |
| Johanna Mattson | Hospital District of Helsinki and Uusimaa, Helsinki, Finland | johanna.mattson@hus.fi | Clinical Groups | Oncology Group |
| Eveliina Salminen | Hospital District of Helsinki and Uusimaa, Helsinki, Finland | eveliina.e.salminen@hus.fi | Clinical Groups | Oncology Group |
| Annika Auranen | Pirkanmaa Hospital District , Tampere, Finland | anaura@utu.fi | Clinical Groups | Oncology Group |
| Peeter Karihtala | Northern Ostrobothnia Hospital District, Oulu, Finland | peeter.karihtala@oulu.fi | Clinical Groups | Oncology Group |
| Päivi Auvinen | Northern Savo Hospital District, Kuopio, Finland | paivi.auvinen@kuh.fi | Clinical Groups | Oncology Group |
| Klaus Elenius | Hospital District of Southwest Finland, Turku, Finland | klaus.elenius@utu.fi | Clinical Groups | Oncology Group |
| Johanna Schleutker | Hospital District of Southwest Finland, Turku, Finland | johanna.schleutker@utu.fi | Clinical Groups | Oncology Group |
| Esa Pitkänen | Institute for Molecular Medicine Finland (FIMM), HiLIFE, University of Helsinki, Helsinki, Finland | esa.pitkanen@helsinki.fi | Clinical Groups | Oncology Group |
| Nina Mars | Institute for Molecular Medicine Finland (FIMM), HiLIFE, University of Helsinki, Helsinki, Finland | nina.mars@helsinki.fi | Clinical Groups | Oncology Group |
| Mark Daly | Institute for Molecular Medicine Finland (FIMM), HiLIFE, University of Helsinki, Helsinki, Finland; Broad Institute of MIT and Harvard; Massachusetts General Hospital | mark.daly@helsinki.fi | Clinical Groups | Oncology Group |
| Relja Popovic | Abbvie, Chicago, IL, United States | relja.popovic@abbvie.com | Clinical Groups | Oncology Group |
| Jeffrey Waring | Abbvie, Chicago, IL, United States | jeff.waring@abbvie.com | Clinical Groups | Oncology Group |
| Bridget Riley-Gillis | Abbvie, Chicago, IL, United States | bridget.rileygillis@abbvie.co | Clinical Groups | Oncology Group |
| Anne Lehtonen | Abbvie, Chicago, IL, United States | anne.lehtonen@abbvie.com | Clinical Groups | Oncology Group |
| Jennifer Schutzman | Genentech, San Francisco, CA, United States | schutzman.jennifer@gene.com | Clinical Groups | Oncology Group |
| Julie Hunkapiller | Genentech, San Francisco, CA, United States | hunkapiller.julie@gene.com | Clinical Groups | Oncology Group |
| Natalie Bowers | Genentech, San Francisco, CA, United States | bowersn1@gene.com | Clinical Groups | Oncology Group |
| Rion Pendergrass | Genentech, San Francisco, CA, United States | penders2@gene.com | Clinical Groups | Oncology Group |
| Diptee Kulkarni | GlaxoSmithKline, Brentford, United Kingdom | diptee.a.kulkarni@gsk.com | Clinical Groups | Oncology Group |
| Kirsi Auro | GlaxoSmithKline, Espoo, Finland | kirsi.m.auro@gsk.com | Clinical Groups | Oncology Group |
| Alessandro Porello | Janssen Research & Development, LLC, Spring House, PA, United States | APorrell@ITS.JNJ.com | Clinical Groups | Oncology Group |
| Andrey Loboda | Merck, Kenilworth, NJ, United States | andrey_loboda@merck.com | Clinical Groups | Oncology Group |
| Heli Lehtonen | Pfizer, New York, NY, United States | heli.lehtonen@pfizer.com | Clinical Groups | Oncology Group |
| Stefan McDonough | Pfizer, New York, NY, United States | stefan.McDonough@pfizer.com | Clinical Groups | Oncology Group |
| Sauli Vuoti | Janssen-Cilag Oy, Espoo, Finland | svuoti@its.jnj.com | Clinical Groups | Oncology Group |
| Kai Kaarniranta | Northern Savo Hospital District, Kuopio, Finland | kai.kaarniranta@uef.fi | Clinical Groups | Opthalmology Group |
| Joni A Turunen | Helsinki University Hospital and University of Helsinki, Helsinki, Finland; Eye Genetics Group, Folkhälsan Research Center, Helsinki, Finland | joni.turunen@helsinki.fi | Clinical Groups | Opthalmology Group |
| Terhi Ollila | Hospital District of Helsinki and Uusimaa, Helsinki, Finland | terhi.ollila@hus.fi | Clinical Groups | Opthalmology Group |
| Hannu Uusitalo | Pirkanmaa Hospital District, Tampere, Finland | hannu.uusitalo@tuni.fi | Clinical Groups | Opthalmology Group |
| Juha Karjalainen | Institute for Molecular Medicine Finland (FIMM), HiLIFE, University of Helsinki, Helsinki, Finland | juha.karjalainen@helsinki.fi | Clinical Groups | Opthalmology Group |
| Esa Pitkänen | Institute for Molecular Medicine Finland (FIMM), HiLIFE, University of Helsinki, Helsinki, Finland | esa.pitkanen@helsinki.fi | Clinical Groups | Opthalmology Group |
| Mengzhen Liu | Abbvie, Chicago, IL, United States | mengzhen.liu@abbvie.com | Clinical Groups | Opthalmology Group |
| Heiko Runz | Biogen, Cambridge, MA, United States | heiko.runz@biogen.com | Clinical Groups | Opthalmology Group |
| Stephanie Loomis | Biogen, Cambridge, MA, United States | stephanie.loomis@biogen.com | Clinical Groups | Opthalmology Group |
| Erich Strauss | Genentech, San Francisco, CA, United States | strauss.erich@gene.com | Clinical Groups | Opthalmology Group |
| Natalie Bowers | Genentech, San Francisco, CA, United States | bowersn1@gene.com | Clinical Groups | Opthalmology Group |
| Hao Chen | Genentech, San Francisco, CA, United States | haoc@gene.com | Clinical Groups | Opthalmology Group |
| Rion Pendergrass | Genentech, San Francisco, CA, United States | penders2@gene.com | Clinical Groups | Opthalmology Group |
| Kaisa Tasanen | Northern Ostrobothnia Hospital District, Oulu, Finland | kaisa.tasanen- | Clinical Groups | Dermatology Group |
| Laura Huilaja | Northern Ostrobothnia Hospital District, Oulu, Finland | laura.huilaja@oulu.fi | Clinical Groups | Dermatology Group |
| Katariina Hannula-Jouppi | Hospital District of Helsinki and Uusimaa, Helsinki, Finland | katariina.hannula-jouppi@hus.fi | Clinical Groups | Dermatology Group |
| Teea Salmi | Pirkanmaa Hospital District, Tampere, Finland | teea.salmi@pshp.fi | Clinical Groups | Dermatology Group |
| Sirkku Peltonen | Hospital District of Southwest Finland, Turku, Finland | sipelto@utu.fi | Clinical Groups | Dermatology Group |
| Leena Koulu | Hospital District of Southwest Finland, Turku, Finland | leena.koulu@tyks.fi | Clinical Groups | Dermatology Group |
| Nizar Smaoui | Abbvie, Chicago, IL, United States | nizar.smaoui@abbvie.com | Clinical Groups | Dermatology Group |
| Fedik Rahimov | Abbvie, Chicago, IL, United States | fedik.rahimov@abbvie.com | Clinical Groups | Dermatology Group |
| Anne Lehtonen | Abbvie, Chicago, IL, United States | anne.lehtonen@abbvie.com | Clinical Groups | Dermatology Group |
| David Choy | Genentech, San Francisco, CA, United States | choy.david@gene.com | Clinical Groups | Dermatology Group |
| Rion Pendergrass | Genentech, San Francisco, CA, United States | penders2@gene.com | Clinical Groups | Dermatology Group |
| Dawn Waterworth | Janssen Research & Development, LLC, Spring House, PA, United States | dwaterwo@its.jnj.com | Clinical Groups | Dermatology Group |
| Kirsi Kalpala | Pfizer, New York, NY, United States | kirsi.kalpala@pfizer.com | Clinical Groups | Dermatology Group |

| | | | | |
|---|---|---|---|---|
| Ying Wu | Pfizer, New York, NY, United States | ying.wu3@pfizer.com | Clinical Groups | Dermatology Group |
| Pirkko Pussinen | Hospital District of Helsinki and Uusimaa, Helsinki, Finland | pirkko.pussinen@helsinki.fi | Clinical Groups | Odontology Group |
| Aino Salminen | Hospital District of Helsinki and Uusimaa, Helsinki, Finland | aino.m.salminen@helsinki.fi | Clinical Groups | Odontology Group |
| Tuula Salo | Hospital District of Helsinki and Uusimaa, Helsinki, Finland | tuula.salo@helsinki.fi | Clinical Groups | Odontology Group |
| David Rice | Hospital District of Helsinki and Uusimaa, Helsinki, Finland | david.rice@helsinki.fi | Clinical Groups | Odontology Group |
| Pekka Nieminen | Hospital District of Helsinki and Uusimaa, Helsinki, Finland | pekka.nieminen@helsinki.fi | Clinical Groups | Odontology Group |
| Ulla Palotie | Hospital District of Helsinki and Uusimaa, Helsinki, Finland | ulla.palotie@helsinki.fi | Clinical Groups | Odontology Group |
| Maria Siponen | Northern Savo Hospital District, Kuopio, Finland | maria.siponen@uef.fi | Clinical Groups | Odontology Group |
| Liisa Suominen | Northern Savo Hospital District, Kuopio, Finland | liisa.suominen@uef.fi | Clinical Groups | Odontology Group |
| Päivi Mäntylä | Northern Savo Hospital District, Kuopio, Finland | paivi.mantyla@uef.fi | Clinical Groups | Odontology Group |
| Ulvi Gursoy | Hospital District of Southwest Finland, Turku, Finland | ulvi.gursoy@utu.fi | Clinical Groups | Odontology Group |
| Vuokko Anttonen | Northern Ostrobothnia Hospital District, Oulu, Finland | vuokko.anttonen@oulu.fi | Clinical Groups | Odontology Group |
| Kirsi Sipilä | Research Unit of Oral Health Sciences Faculty of Medicine, University of Oulu, Oulu, Finland; Medical Research Center, Oulu, Oulu University Hospital and University of Oulu, Oulu, Finland | kirsi.sipila@oulu.fi | Clinical Groups | Odontology Group |
| Rion Pendergrass | Genentech, San Francisco, CA, United States | pendergrass.sarah@gene.com | Clinical Groups | Odontology Group |
| Hannele Laivuori | Institute for Molecular Medicine Finland (FIMM), HiLIFE, University of Helsinki, Helsinki, Finland | hannele.laivuori@helsinki.fi | Clinical Groups | Women's Health and Reproduction Group |
| Venla Kurra | Pirkanmaa Hospital District, Tampere, Finland | venla.kurra@tuni.fi | Clinical Groups | Women's Health and Reproduction Group |
| Laura Kotaniemi-Talonen | Pirkanmaa Hospital District, Tampere, Finland | laura.kotaniemi-talonen@tuni.fi | Clinical Groups | Women's Health and Reproduction Group |
| Oskari Heikinheimo | Hospital District of Helsinki and Uusimaa, Helsinki, Finland | oskari.heikinheimo@helsinki.fi | Clinical Groups | Women's Health and Reproduction Group |
| Ilkka Kalliala | Hospital District of Helsinki and Uusimaa, Helsinki, Finland | ilkka.kalliala@hus.fi | Clinical Groups | Women's Health and Reproduction Group |
| Lauri Aaltonen | Hospital District of Helsinki and Uusimaa, Helsinki, Finland | lauri.aaltonen@helsinki.fi | Clinical Groups | Women's Health and Reproduction Group |
| Varpu Jokimaa | Hospital District of Southwest Finland, Turku, Finland | varpu.jokimaa@utu.fi | Clinical Groups | Women's Health and Reproduction Group |
| Johannes Kettunen | Northern Ostrobothnia Hospital District, Oulu, Finland | Johannes.Kettunen@oulu.fi | Clinical Groups | Women's Health and Reproduction Group |
| Marja Vääräsmäki | Northern Ostrobothnia Hospital District, Oulu, Finland | marja.vaarasmaki@oulu.fi | Clinical Groups | Women's Health and Reproduction Group |
| Outi Uimari | Northern Ostrobothnia Hospital District, Oulu, Finland | outi.uimari@oulu.fi | Clinical Groups | Women's Health and Reproduction Group |
| Laure Morin-Papunen | Northern Ostrobothnia Hospital District, Oulu, Finland | lmp@cc.oulu.fi | Clinical Groups | Women's Health and Reproduction Group |
| Maarit Niinimäki | Northern Ostrobothnia Hospital District, Oulu, Finland | maarit.niinimaki@oulu.fi | Clinical Groups | Women's Health and Reproduction Group |
| Terhi Piltonen | Northern Ostrobothnia Hospital District, Oulu, Finland | terhi.piltonen@oulu.fi | Clinical Groups | Women's Health and Reproduction Group |
| Katja Kivinen | Institute for Molecular Medicine Finland (FIMM), HiLIFE, University of Helsinki, Helsinki, Finland | katja.kivinen@helsinki.fi | Clinical Groups | Women's Health and Reproduction Group |
| Elisabeth Widen | Institute for Molecular Medicine Finland (FIMM), HiLIFE, University of Helsinki, Helsinki, Finland | elisabeth.widen@helsinki.fi | Clinical Groups | Women's Health and Reproduction Group |
| Taru Tukiainen | Institute for Molecular Medicine Finland (FIMM), HiLIFE, University of Helsinki, Helsinki, Finland | taru.tukiainen@helsinki.fi | Clinical Groups | Women's Health and Reproduction Group |
| Mary Pat Reeve | Institute for Molecular Medicine Finland (FIMM), HiLIFE, University of Helsinki, Helsinki, Finland | mary.reeve@helsinki.fi | Clinical Groups | Women's Health and Reproduction Group |
| Mark Daly | Institute for Molecular Medicine Finland (FIMM), HiLIFE, University of Helsinki, Helsinki, Finland; Broad Institute of MIT and Harvard; Massachusetts General Hospital | mark.daly@helsinki.fi | Clinical Groups | Women's Health and Reproduction Group |
| Niko Välimäki | University of Helsinki, Helsinki, Finland | niko.valimaki@helsinki.fi | Clinical Groups | Women's Health and Reproduction Group |
| Eija Laakkonen | University of Jyväskylä, Jyväskylä, Finland | eija.k.laakkonen@jyu.fi | Clinical Groups | Women's Health and Reproduction Group |
| Jaakko Tyrmi | University of Oulu, Oulu, Finland / University of Tampere, Tampere, Finland | jaakko.tyrmi@oulu.fi | Clinical Groups | Women's Health and Reproduction Group |
| Heidi Silven | University of Oulu, Oulu, Finland | heidi.silven@student.oulu.fi | Clinical Groups | Women's Health and Reproduction Group |
| Eeva Sliz | University of Oulu, Oulu, Finland | eeva.sliz@oulu.fi | Clinical Groups | Women's Health and Reproduction Group |
| Riikka Arffman | University of Oulu, Oulu, Finland | riikka.arffman@oulu.fi | Clinical Groups | Women's Health and Reproduction Group |
| Susanna Savukoski | University of Oulu, Oulu, Finland | susanna.savukoski@oulu.fi | Clinical Groups | Women's Health and Reproduction Group |
| Triin Laisk | Estonian biobank, Tartu, Estonia | triin.laisk@ut.ee | Clinical Groups | Women's Health and Reproduction Group |
| Natalia Pujol | Estonian biobank, Tartu, Estonia | natalia.pujolgualdo@oulu.fi | Clinical Groups | Women's Health and Reproduction Group |
| Mengzhen Liu | Abbvie, Chicago, IL, United States | mengzhen.liu@abbvie.com | Clinical Groups | Women's Health and Reproduction Group |
| Bridget Riley-Gillis | Abbvie, Chicago, IL, United States | bridget.rileygillis@abbvie.com | Clinical Groups | Women's Health and Reproduction Group |
| Rion Pendergrass | Genentech, San Francisco, CA, United States | penders2@gene.com | Clinical Groups | Women's Health and Reproduction Group |
| Janet Kumar | GlaxoSmithKline, Collegeville, PA, United States | janet.x.kumar@gsk.com | Clinical Groups | Women's Health and Reproduction Group |
| Kirsi Auro | GlaxoSmithKline, Espoo, Finland | kirsi.m.auro@gsk.com | Clinical Groups | Women's Health and Reproduction Group |
| Iiris Hovatta | University of Helsinki, Helsinki, Finland | iiris.hovatta@helsinki.fi | Clinical Groups | Depression group |
| Chia-Yen Chen | Biogen, Cambridge, MA, United States | chiayen.chen@biogen.com | Clinical Groups | Depression group |
| Erkki Isometsä | Hospital District of Helsinki and Uusimaa, Helsinki, Finland | erkki.isometsa@hus.fi | Clinical Groups | Depression group |
| Hanna Ollila | Institute for Molecular Medicine Finland (FIMM), HiLIFE, University of Helsinki, Helsinki, Finland | hanna.m.ollila@helsinki.fi | Clinical Groups | Depression group |
| Jaana Suvisaari | Finnish Institute for Health and Welfare (THL), Helsinki, Finland | jaana.suvisaari@thl.fi | Clinical Groups | Depression group |
| Thomas Damm Als | Aarhus University, Denmark | tda@biomed.au.dk | Clinical Groups | Depression group |

| | | | | |
|---|---|---|---|---|
| Antti Mäkitie | Department of Otorhinolaryngology - Head and Neck Surgery, University of Helsinki and Helsinki University Hospital, Helsinki, Finland | antti.makitie@helsinki.fi | Clinical Groups | ENT (ear, nose and throath) Group |
| Argyro Bizaki-Vallaskangas | Pirkanmaa Hospital District, Tampere, Finland | argyro.bizaki-vallaskangas@tuni.fi | Clinical Groups | ENT (ear, nose and throath) Group |
| Sanna Toppila-Salmi | University of Helsinki, Finland | sanna.salmi@helsinki.fi | Clinical Groups | ENT (ear, nose and throath) Group |
| Tytti Willberg | Hospital District of Southwest Finland, Turku, Finland | tytti.willberg@tyks.fi | Clinical Groups | ENT (ear, nose and throath) Group |
| Elmo Saarentaus | Institute for Molecular Medicine Finland (FIMM), HiLIFE, University of Helsinki, Helsinki, Finland | elmo.saarentaus@helsinki.fi | Clinical Groups | ENT (ear, nose and throath) Group |
| Antti Aarnisalo | Hospital District of Helsinki and Uusimaa, Helsinki, Finland | antti.aarnisalo@hus.fi | Clinical Groups | ENT (ear, nose and throath) Group |
| Eveliina Salminen | Hospital District of Helsinki and Uusimaa, Helsinki, Finland | eveliina.e.salminen@hus.fi | Clinical Groups | ENT (ear, nose and throath) Group |
| Elisa Rahikkala | Northern Ostrobothnia Hospital District, Oulu, Finland | elisa.rahikkala@ppshp.fi | Clinical Groups | ENT (ear, nose and throath) Group |
| Johannes Kettunen | Northern Ostrobothnia Hospital District, Oulu, Finland | johannes.kettunen@oulu.fi | Clinical Groups | ENT (ear, nose and throath) Group |
| Kristiina Aittomäki | Department of Medical Genetics, Helsinki University Central Hospital, Helsinki, Finland | kristiina.aittomaki@helsinki.fi | Clinical Groups | POI (premature ovarian failure) Group |
| Fredrik Åberg | Transplantation and Liver Surgery Clinic, Helsinki University Hospital, Helsinki University, Helsinki, Finland | fredrik.aberg@helsinki.fi | Clinical Groups | LiverScore Group |
| Mitja Kurki | Institute for Molecular Medicine Finland (FIMM), HiLIFE, University of Helsinki, Helsinki, Finland; Broad Institute, Cambridge, MA, United States | mkurki@broadinstitute.org | FinnGen Analysis working group | FinnGen Analysis working group |
| Samuli Ripatti | Institute for Molecular Medicine Finland (FIMM), HiLIFE, University of Helsinki, Helsinki, Finland | samuli.ripatti@helsinki.fi | FinnGen Analysis working group | FinnGen Analysis working group |
| Mark Daly | Institute for Molecular Medicine, Finland (FIMM), HiLIFE, University of Helsinki, Helsinki, Finland; Broad Institute of MIT and Harvard; Massachusetts General Hospital | mark.daly@helsinki.fi | FinnGen Analysis working group | FinnGen Analysis working group |
| Juha Karjalainen | Institute for Molecular Medicine Finland (FIMM), HiLIFE, University of Helsinki, Helsinki, Finland | juha.karjalainen@helsinki.fi | FinnGen Analysis working group | FinnGen Analysis working group |
| Aki Havulinna | Institute for Molecular Medicine Finland (FIMM), HiLIFE, University of Helsinki, Helsinki, Finland; Finnish Institute for Health and Welfare (THL), Helsinki, Finland | aki.havulinna@helsinki.fi | FinnGen Analysis working group | FinnGen Analysis working group |
| Juha Mehtonen | Institute for Molecular Medicine Finland (FIMM), HiLIFE, University of Helsinki, Helsinki, Finland | juha.mehtonen@helsinki.fi | FinnGen Analysis working group | FinnGen Analysis working group |
| Priit Palta | Institute for Molecular Medicine Finland (FIMM), HiLIFE, University of Helsinki, Helsinki, Finland | priit.palta@helsinki.fi | FinnGen Analysis working group | FinnGen Analysis working group |
| Shabbeer Hassan | Institute for Molecular Medicine Finland (FIMM), HiLIFE, University of Helsinki, Helsinki, Finland | shabbeer.hassan@helsinki.fi | FinnGen Analysis working group | FinnGen Analysis working group |
| Pietro Della Briotta Parolo | Institute for Molecular Medicine Finland (FIMM), HiLIFE, University of Helsinki, Helsinki, Finland | pietro.dellabriottaparolo@helsinki.fi | FinnGen Analysis working group | FinnGen Analysis working group |
| Wei Zhou | Broad Institute, Cambridge, MA, United States | wzhou@broadinstitute.org | FinnGen Analysis working group | FinnGen Analysis working group |
| Mutaamba Maasha | Broad Institute, Cambridge, MA, United States | mmaasha@broadinstitute.org | FinnGen Analysis working group | FinnGen Analysis working group |
| Shabbeer Hassan | Institute for Molecular Medicine Finland (FIMM), HiLIFE, University of Helsinki, Helsinki, Finland | shabbeer.hassan@helsinki.fi | FinnGen Analysis working group | FinnGen Analysis working group |
| Susanna Lemmelä | Institute for Molecular Medicine Finland (FIMM), HiLIFE, University of Helsinki, Helsinki, Finland | susanna.lemmela@helsinki.fi | FinnGen Analysis working group | FinnGen Analysis working group |
| Manuel Rivas | University of Stanford, Stanford, CA, United States | mrivas@stanford.edu | FinnGen Analysis working group | FinnGen Analysis working group |
| Mari E. Niemi | Institute for Molecular Medicine Finland (FIMM), HiLIFE, University of Helsinki, Helsinki, Finland | mari.e.niemi@helsinki.fi | FinnGen Analysis working group | FinnGen Analysis working group |
| Aarno Palotie | Institute for Molecular Medicine Finland (FIMM), HiLIFE, University of Helsinki, Helsinki, Finland | aarno.palotie@helsinki.fi | FinnGen Analysis working group | FinnGen Analysis working group |
| Aoxing Liu | Institute for Molecular Medicine Finland (FIMM), HiLIFE, University of Helsinki, Helsinki, Finland | aoxing.liu@helsinki.fi | FinnGen Analysis working group | FinnGen Analysis working group |
| Arto Lehisto | Institute for Molecular Medicine Finland (FIMM), HiLIFE, University of Helsinki, Helsinki, Finland | arto.lehisto@helsinki.fi | FinnGen Analysis working group | FinnGen Analysis working group |
| Andrea Ganna | Institute for Molecular Medicine Finland (FIMM), HiLIFE, University of Helsinki, Helsinki, Finland | aganna@broadinstitute.org | FinnGen Analysis working group | FinnGen Analysis working group |
| Vincent Llorens | Institute for Molecular Medicine Finland (FIMM), HiLIFE, University of Helsinki, Helsinki, Finland | vincent.llorens@helsinki.fi | FinnGen Analysis working group | FinnGen Analysis working group |
| Hannele Laivuori | Institute for Molecular Medicine Finland (FIMM), HiLIFE, University of Helsinki, Helsinki, Finland | hannele.laivuori@helsinki.fi | FinnGen Analysis working group | FinnGen Analysis working group |
| Taru Tukiainen | Institute for Molecular Medicine Finland (FIMM), HiLIFE, University of Helsinki, Helsinki, Finland | taru.tukiainen@helsinki.fi | FinnGen Analysis working group | FinnGen Analysis working group |
| Mary Pat Reeve | Institute for Molecular Medicine Finland (FIMM), HiLIFE, University of Helsinki, Helsinki, Finland | mary.reeve@helsinki.fi | FinnGen Analysis working group | FinnGen Analysis working group |
| Henrike Heyne | Institute for Molecular Medicine Finland (FIMM), HiLIFE, University of Helsinki, Helsinki, Finland | hheyne@broadinstitute.org | FinnGen Analysis working group | FinnGen Analysis working group |
| Nina Mars | Institute for Molecular Medicine Finland (FIMM), HiLIFE, University of Helsinki, Helsinki, Finland | nina.mars@helsinki.fi | FinnGen Analysis working group | FinnGen Analysis working group |
| Joel Rämö | Institute for Molecular Medicine Finland (FIMM), HiLIFE, University of Helsinki, Helsinki, Finland | joel.ramo@helsinki.fi | FinnGen Analysis working group | FinnGen Analysis working group |
| Elmo Saarentaus | Institute for Molecular Medicine Finland (FIMM), HiLIFE, University of Helsinki, Helsinki, Finland | elmo.saarentaus@helsinki.fi | FinnGen Analysis working group | FinnGen Analysis working group |
| Hanna Ollila | Institute for Molecular Medicine Finland (FIMM), HiLIFE, University of Helsinki, Helsinki, Finland | hanna.m.ollila@helsinki.fi | FinnGen Analysis working group | FinnGen Analysis working group |
| Rodos Rodosthenous | Institute for Molecular Medicine Finland (FIMM), HiLIFE, University of Helsinki, Helsinki, Finland | rodos.rodosthenous@helsinki.fi | FinnGen Analysis working group | FinnGen Analysis working group |
| Satu Strausz | Institute for Molecular Medicine Finland (FIMM), HiLIFE, University of Helsinki, Helsinki, Finland | satu.strausz@helsinki.fi | FinnGen Analysis working group | FinnGen Analysis working group |
| Tuula Palotie | University of Helsinki and Hospital District of Helsinki and Uusimaa, Helsinki, Finland | tuula.palotie@helsinki.fi | FinnGen Analysis working group | FinnGen Analysis working group |
| Kimmo Palin | University of Helsinki, Helsinki, Finland | kimmo.palin@helsinki.fi | FinnGen Analysis working group | FinnGen Analysis working group |
| Javier Garcia-Tabuenca | University of Tampere, Tampere, Finland | javier.graciatabuenca@tuni.fi | FinnGen Analysis working group | FinnGen Analysis working group |
| Harri Siirtola | University of Tampere, Tampere, Finland | harri.siirtola@tuni.fi | FinnGen Analysis working group | FinnGen Analysis working group |

| | | | | |
|---|---|---|---|---|
| Tuomo Kiiskinen | Institute for Molecular Medicine Finland (FIMM), HiLIFE, University of Helsinki, Helsinki, Finland | tuomo.kiiskinen@helsinki.fi | FinnGen Analysis working group | FinnGen Analysis working group |
| Jiwoo Lee | Institute for Molecular Medicine Finland (FIMM), HiLIFE, University of Helsinki, Helsinki, Finland; Broad Institute, Cambridge, MA, United States | jiwoo.lee@helsinki.fi | FinnGen Analysis working group | FinnGen Analysis working group |
| Kristin Tsuo | Institute for Molecular Medicine Finland (FIMM), HiLIFE, University of Helsinki, Helsinki, Finland; Broad Institute, Cambridge, MA, United States | kristintsuo@fas.harvard.edu | FinnGen Analysis working group | FinnGen Analysis working group |
| Amanda Elliott | Institute for Molecular Medicine Finland (FIMM), HiLIFE, University of Helsinki, Helsinki, Finland; Broad Institute, Cambridge, MA, USA and Massachusetts General Hospital, Boston, MA, USA | aelliott@broadinstitute.org | FinnGen Analysis working group | FinnGen Analysis working group |
| Kati Kristiansson | THL Biobank / Finnish Institute for Health and Welfare (THL), Helsinki, Finland | kati.kristiansson@thl.fi | FinnGen Analysis working group | FinnGen Analysis working group |
| Mikko Arvas | Finnish Red Cross Blood Service / Finnish Hematology Registry and Clinical Biobank, Helsinki, Finland | mikko.arvas@veripalvelu.fi | FinnGen Analysis working group | FinnGen Analysis working group |
| Kati Hyvärinen | Finnish Red Cross Blood Service, Helsinki, Finland | kati.hyvarinen@veripalvelu.fi | FinnGen Analysis working group | FinnGen Analysis working group |
| Jarmo Ritari | Finnish Red Cross Blood Service, Helsinki, Finland | jarmo.ritari@veripalvelu.fi | FinnGen Analysis working group | FinnGen Analysis working group |
| Olli Carpén | Helsinki Biobank / Helsinki University and Hospital District of Helsinki and Uusimaa, Helsinki | olli.carpen@helsinki.fi | FinnGen Analysis working group | FinnGen Analysis working group |
| Johannes Kettunen | Northern Finland Biobank Borealis / University of Oulu / Northern Ostrobothnia Hospital District, Oulu, Finland | johannes.kettunen@oulu.fi | FinnGen Analysis working group | FinnGen Analysis working group |
| Katri Pylkäs | University of Oulu, Oulu, Finland | katri.pylkas@oulu.fi | FinnGen Analysis working group | FinnGen Analysis working group |
| Eeva Sliz | University of Oulu, Oulu, Finland | eeva.sliz@oulu.fi | FinnGen Analysis working group | FinnGen Analysis working group |
| Minna Karjalainen | University of Oulu, Oulu, Finland | minna.k.karjalainen@oulu.fi | FinnGen Analysis working group | FinnGen Analysis working group |
| Tuomo Mantere | Northern Finland Biobank Borealis / University of Oulu / Northern Ostrobothnia Hospital District, Oulu, Finland | tuomo.mantere@oulu.fi | FinnGen Analysis working group | FinnGen Analysis working group |
| Eeva Kangasniemi | Finnish Clinical Biobank Tampere / University of Tampere / Pirkanmaa Hospital District, Tampere, Finland | eeva.kangasniemi@pshp.fi | FinnGen Analysis working group | FinnGen Analysis working group |
| Sami Heikkinen | University of Eastern Finland, Kuopio, Finland | sami.heikkinen@uef.fi | FinnGen Analysis working group | FinnGen Analysis working group |
| Arto Mannermaa | Biobank of Eastern Finland / University of Eastern Finland / Northern Savo Hospital District, Kuopio, Finland | arto.mannermaa@uef.fi | FinnGen Analysis working group | FinnGen Analysis working group |
| Eija Laakkonen | University of Jyväskylä, Jyväskylä, Finland | eija.k.laakkonen@jyu.fi | FinnGen Analysis working group | FinnGen Analysis working group |
| Nina Pitkänen | Auria Biobank / University of Turku / Hospital District of Southwest Finland, Turku, Finland | Niina.Pitkanen@tyks.fi | FinnGen Analysis working group | FinnGen Analysis working group |
| Samuel Lessard | Translational Sciences, Sanofi R&D, Framingham, MA, USA | samuel.lessard@sanofi.com | FinnGen Analysis working group | FinnGen Analysis working group |
| Clément Chatelain | Translational Sciences, Sanofi R&D, Framingham, MA, USA | clement.chatelain@sanofi.co m | FinnGen Analysis working group | FinnGen Analysis working group |
| Perttu Terho | Auria Biobank / University of Turku / Hospital District of Southwest Finland, Turku, Finland | perttu.terho@tyks.fi | Biobank directors | Biobank directors |
| Sirpa Soini | THL Biobank / Finnish Institute for Health and Welfare (THL), Helsinki, Finland | sirpa.soini@thl.fi | Biobank directors | Biobank directors |
| Jukka Partanen | Finnish Red Cross Blood Service / Finnish Hematology Registry and Clinical Biobank, Helsinki, Finland | jukka.partanen@veripalvelu.fi | Biobank directors | Biobank directors |
| Eero Punkka | Helsinki Biobank / Helsinki University and Hospital District of Helsinki and Uusimaa, Helsinki | eero.punkka@hus.fi | Biobank directors | Biobank directors |
| Raisa Serpi | Northern Finland Biobank Borealis / University of Oulu / Northern Ostrobothnia Hospital District, Oulu, Finland | raisa.serpi@ppshp.fi | Biobank directors | Biobank directors |
| Sanna Siltanen | Finnish Clinical Biobank Tampere / University of Tampere / Pirkanmaa Hospital District, Tampere, Finland | sanna.siltanen@pshp.fi | Biobank directors | Biobank directors |
| Veli-Matti Kosma | Biobank of Eastern Finland / University of Eastern Finland / Northern Savo Hospital District, Kuopio, Finland | veli-matti.kosma@uef.fi | Biobank directors | Biobank directors |
| Teijo Kuopio | Central Finland Biobank / University of Jyväskylä / Central Finland Health Care District, Jyväskylä, Finland | teijo.kuopio@ksshp.fi | Biobank directors | Biobank directors |
| Anu Jalanko | Institute for Molecular Medicine Finland (FIMM), HiLIFE, University of Helsinki, Helsinki, Finland | anu.jalanko@helsinki.fi | FinnGen Teams | Administration |
| Huei-Yi Shen | Institute for Molecular Medicine Finland (FIMM), HiLIFE, University of Helsinki, Helsinki, Finland | huei-yi.shen@helsinki.fi | FinnGen Teams | Administration |
| Risto Kajanne | Institute for Molecular Medicine Finland (FIMM), HiLIFE, University of Helsinki, Helsinki, Finland | risto.kajanne@helsinki.fi | FinnGen Teams | Administration |
| Mervi Aavikko | Institute for Molecular Medicine Finland (FIMM), HiLIFE, University of Helsinki, Helsinki, Finland | mervi.aavikko@helsinki.fi | FinnGen Teams | Administration |
| Henna Palin | Finnish Clinical Biobank Tampere / University of Tampere / Pirkanmaa Hospital District, Tampere, Finland | henna.palin@pshp.fi | FinnGen Teams | Administration |
| Malla-Maria Linna | Helsinki Biobank / Helsinki University and Hospital District of Helsinki and Uusimaa, Helsinki | malla-maria.linna@hus.fi | FinnGen Teams | Administration |
| Mitja Kurki | Institute for Molecular Medicine Finland (FIMM), HiLIFE, University of Helsinki, Helsinki, Finland; Broad Institute, Cambridge, MA, United States | mkurki@broadinstitute.org | FinnGen Teams | Analysis |
| Juha Karjalainen | Institute for Molecular Medicine Finland (FIMM), HiLIFE, University of Helsinki, Helsinki, Finland | juha.karjalainen@helsinki.fi | FinnGen Teams | Analysis |
| Pietro Della Briotta Parolo | Institute for Molecular Medicine Finland (FIMM), HiLIFE, University of Helsinki, Helsinki, Finland | pietro.dellabriottaparolo@hel sinki.fi | FinnGen Teams | Analysis |
| Arto Lehisto | Institute for Molecular Medicine Finland (FIMM), HiLIFE, University of Helsinki, Helsinki, Finland | arto.lehisto@helsinki.fi | FinnGen Teams | Analysis |
| Juha Mehtonen | Institute for Molecular Medicine Finland (FIMM), HiLIFE, University of Helsinki, Helsinki, Finland | juha.mehtonen@helsinki.fi | FinnGen Teams | Analysis |
| Wei Zhou | Broad Institute, Cambridge, MA, United States | wzhou@broadinstitute.org | FinnGen Teams | Analysis |
| Masahiro Kanai | Broad Institute, Cambridge, MA, United States | mkanai@broadinstitute.org | FinnGen Teams | Analysis |
| Mutaamba Maasha | Broad Institute, Cambridge, MA, United States | mmaasha@broadinstitute.org | FinnGen Teams | Analysis |
| Hannele Laivuori | Institute for Molecular Medicine Finland (FIMM), HiLIFE, University of Helsinki, Helsinki, Finland | hannele.laivuori@helsinki.fi | FinnGen Teams | Clinical Endpoint Development |
| Aki Havulinna | Institute for Molecular Medicine Finland (FIMM), HiLIFE, University of Helsinki, Helsinki, Finland; Finnish Institute for Health and Welfare (THL), Helsinki, Finland | aki.havulinna@helsinki.fi | FinnGen Teams | Clinical Endpoint Development |
| Susanna Lemmelä | Institute for Molecular Medicine Finland (FIMM), HiLIFE, University of Helsinki, Helsinki, Finland | susanna.lemmela@helsinki.fi | FinnGen Teams | Clinical Endpoint Development |

| | | | | |
|---|---|---|---|---|
| Tuomo Kiiskinen | Institute for Molecular Medicine Finland (FIMM), HiLIFE, University of Helsinki, Helsinki, Finland | tuomo.kiiskinen@helsinki.fi | FinnGen Teams | Clinical Endpoint Development |
| L. Elisa Lahtela | Institute for Molecular Medicine Finland (FIMM), HiLIFE, University of Helsinki, Helsinki, Finland | laura.lahtela@helsinki.fi | FinnGen Teams | Clinical Endpoint Development |
| Mari Kaunisto | Institute for Molecular Medicine Finland (FIMM), HiLIFE, University of Helsinki, Helsinki, Finland | mari.kaunisto@helsinki.fi | FinnGen Teams | Communication |
| Elina Kilpeläinen | Institute for Molecular Medicine Finland (FIMM), HiLIFE, University of Helsinki, Helsinki, Finland | elina.kilpelainen@helsinki.fi | FinnGen Teams | E-Science |
| Timo P. Sipilä | Institute for Molecular Medicine Finland (FIMM), HiLIFE, University of Helsinki, Helsinki, Finland | timo.p.sipila@helsinki.fi | FinnGen Teams | E-Science |
| Oluwaseun Alexander Dada | Institute for Molecular Medicine Finland (FIMM), HiLIFE, University of Helsinki, Helsinki, Finland | alexander.dada@helsinki.fi | FinnGen Teams | E-Science |
| Awaisa Ghazal | Institute for Molecular Medicine Finland (FIMM), HiLIFE, University of Helsinki, Helsinki, Finland | awaisa.ghazal@helsinki.fi | FinnGen Teams | E-Science |
| Anastasia Kytölä | Institute for Molecular Medicine Finland (FIMM), HiLIFE, University of Helsinki, Helsinki, Finland | anastasia.shcherban@helsinki.fi | FinnGen Teams | E-Science |
| Rigbe Weldatsadik | Institute for Molecular Medicine Finland (FIMM), HiLIFE, University of Helsinki, Helsinki, Finland | rigbe.weldatsadik@helsinki.fi | FinnGen Teams | E-Science |
| Sanni Ruotsalainen | Institute for Molecular Medicine Finland (FIMM), HiLIFE, University of Helsinki, Helsinki, Finland | sanni.ruotsalainen@helsinki.fi | FinnGen Teams | E-Science |
| Kati Donner | Institute for Molecular Medicine Finland (FIMM), HiLIFE, University of Helsinki, Helsinki, Finland | kati.donner@helsinki.fi | FinnGen Teams | Genotyping |
| Timo P. Sipilä | Institute for Molecular Medicine Finland (FIMM), HiLIFE, University of Helsinki, Helsinki, Finland | timo.p.sipila@helsinki.fi | FinnGen Teams | Genotyping |
| Anu Loukola | Helsinki Biobank / Helsinki University and Hospital District of Helsinki and Uusimaa, Helsinki | anu.loukola@hus.fi | FinnGen Teams | Sample Collection Coordination |
| Päivi Laiho | THL Biobank / Finnish Institute for Health and Welfare (THL), Helsinki, Finland | paivi.laiho@thl.fi | FinnGen Teams | Sample Logistics |
| Tuuli Sistonen | THL Biobank / Finnish Institute for Health and Welfare (THL), Helsinki, Finland | tuuli.sistonen@thl.fi | FinnGen Teams | Sample Logistics |
| Essi Kaiharju | THL Biobank / Finnish Institute for Health and Welfare (THL), Helsinki, Finland | essi.kaiharju@thl.fi | FinnGen Teams | Sample Logistics |
| Markku Laukkanen | THL Biobank / Finnish Institute for Health and Welfare (THL), Helsinki, Finland | markku.laukkanen@thl.fi | FinnGen Teams | Sample Logistics |
| Elina Järvensivu | THL Biobank / Finnish Institute for Health and Welfare (THL), Helsinki, Finland | elina.jarvensivu@thl.fi | FinnGen Teams | Sample Logistics |
| Sini Lähteenmäki | THL Biobank / Finnish Institute for Health and Welfare (THL), Helsinki, Finland | sini.lahteenmaki@thl.fi | FinnGen Teams | Sample Logistics |
| Lotta Männikkö | THL Biobank / Finnish Institute for Health and Welfare (THL), Helsinki, Finland | lotta.mannikko@thl.fi | FinnGen Teams | Sample Logistics |
| Regis Wong | THL Biobank / Finnish Institute for Health and Welfare (THL), Helsinki, Finland | regis.wong@thl.fi | FinnGen Teams | Sample Logistics |
| Auli Toivola | THL Biobank / Finnish Institute for Health and Welfare (THL), Helsinki, Finland | auli.toivola@thl.fi | FinnGen Teams | Sample Logistics |
| Minna Brunfeldt | THL Biobank / Finnish Institute for Health and Welfare (THL), Helsinki, Finland | minna.brunfeldt@thl.fi | FinnGen Teams | Registry Data Operations |
| Hannele Mattsson | THL Biobank / Finnish Institute for Health and Welfare (THL), Helsinki, Finland | hannele.mattsson@thl.fi | FinnGen Teams | Registry Data Operations |
| Kati Kristiansson | THL Biobank / Finnish Institute for Health and Welfare (THL), Helsinki, Finland | kati.kristiansson@thl.fi | FinnGen Teams | Registry Data Operations |
| Susanna Lemmelä | Institute for Molecular Medicine Finland (FIMM), HiLIFE, University of Helsinki, Helsinki, Finland | susanna.lemmela@helsinki.fi | FinnGen Teams | Registry Data Operations |
| Sami Koskelainen | THL Biobank / Finnish Institute for Health and Welfare (THL), Helsinki, Finland | sami.koskelainen@thl.fi | FinnGen Teams | Registry Data Operations |
| Tero Hiekkalinna | THL Biobank / Finnish Institute for Health and Welfare (THL), Helsinki, Finland | tero.hiekkalinna@helsinki.fi | FinnGen Teams | Registry Data Operations |
| Teemu Paajanen | THL Biobank / Finnish Institute for Health and Welfare (THL), Helsinki, Finland | teemu.paajanen@thl.fi | FinnGen Teams | Registry Data Operations |
| Priit Palta | Institute for Molecular Medicine Finland (FIMM), HiLIFE, University of Helsinki, Helsinki, Finland | priit.palta@helsinki.fi | FinnGen Teams | Sequencing Informatics |
| Kalle Pärn | Institute for Molecular Medicine Finland (FIMM), HiLIFE, University of Helsinki, Helsinki, Finland | kalle.parn@helsinki.fi | FinnGen Teams | Sequencing Informatics |
| Mart Kals | Institute for Molecular Medicine Finland (FIMM), HiLIFE, University of Helsinki, Helsinki, Finland | mart.kals@helsinki.fi | FinnGen Teams | Sequencing Informatics |
| Shuang Luo | Institute for Molecular Medicine Finland (FIMM), HiLIFE, University of Helsinki, Helsinki, Finland | shuang.luo@helsinki.fi | FinnGen Teams | Sequencing Informatics |
| Tarja Laitinen | Pirkanmaa Hospital District, Tampere, Finland | tarja.laitinen@pshp.fi | FinnGen Teams | Trajectory |
| Mary Pat Reeve | Institute for Molecular Medicine Finland (FIMM), HiLIFE, University of Helsinki, Helsinki, Finland | mary.reeve@helsinki.fi | FinnGen Teams | Trajectory |
| Shanmukha Sampath Padmanabhuni | Institute for Molecular Medicine Finland (FIMM), HiLIFE, University of Helsinki, Helsinki, Finland | sam.padmanabhuni@helsinki.fi | FinnGen Teams | Trajectory |
| Marianna Niemi | University of Tampere, Tampere, Finland | marianna.niemi@tuni.fi | FinnGen Teams | Trajectory |
| Harri Siirtola | University of Tampere, Tampere, Finland | harri.siirtola@tuni.fi | FinnGen Teams | Trajectory |
| Javier Gracia-Tabuenca | University of Tampere, Tampere, Finland | javier.graciatabuenca@tuni.fi | FinnGen Teams | Trajectory |
| Mika Helminen | University of Tampere, Tampere, Finland | mika.helminen@tuni.fi | FinnGen Teams | Trajectory |
| Tiina Luukkaala | University of Tampere, Tampere, Finland | tiina.luukkaala@tuni.fi | FinnGen Teams | Trajectory |
| Iida Vähätalo | University of Tampere, Tampere, Finland | iida.vahatalo@epshp.fi | FinnGen Teams | Trajectory |
| Jyrki Pitkänen | Institute for Molecular Medicine Finland (FIMM), HiLIFE, University of Helsinki, Helsinki, Finland | jyrki.pitkanen@helsinki.fi | FinnGen Teams | Data protection officer |
| Marco Hautalahti | Finnish Biobank Cooperative - FINBB | marco.hautalahti@finbb.fi | FinnGen Teams | FINBB - Finnish biobank cooperative |
| Johanna Mäkelä | Finnish Biobank Cooperative - FINBB | johanna.makela@finbb.fi | FinnGen Teams | FINBB - Finnish biobank cooperative |
| Sarah Smith | Finnish Biobank Cooperative - FINBB | sarah.smith@finbb.fi | FinnGen Teams | FINBB - Finnish biobank cooperative |
| Tom Southerington | Finnish Biobank Cooperative - FINBB | tom.southerington@finbb.fi | FinnGen Teams | FINBB - Finnish biobank cooperative |

**Supplementary Table 9: Finngen.** List of Finngen authors.

# 3. Supplementary References

Acosta-Herrera, M., Kerick, M., González-Serna, D., Wijmenga, C., Franke, A., Gregersen, P.K., Padyukov, L., Worthington, J., Vyse, T.J., Alarcón-Riquelme, M.E., et al. (2019). Genome-wide meta-analysis reveals shared new loci in systemic seropositive rheumatic diseases. Ann Rheum Dis 78, 311-319.

Allam, J.P., Niederhagen, B., Bücheler, M., Appel, T., Betten, H., Bieber, T., Bergé, S., and Novak, N. (2006). Comparative analysis of nasal and oral mucosa dendritic cells. Allergy 61, 166-172.

Allam, J.P., Novak, N., Fuchs, C., Asen, S., Bergé, S., Appel, T., Geiger, E., Kochan, J.P., and Bieber, T. (2003). Characterization of dendritic cells from human oral mucosa: a new Langerhans' cell type with high constitutive FcepsilonRI expression. J Allergy Clin Immunol 112, 141-148.

Aswath, N., Swamikannu, B., Ramakrishnan, S.N., Shanmugam, R., Thomas, J., and Ramanathan, A. (2014). Heterozygous Ile453Val codon mutation in exon 7, homozygous single nucleotide polymorphisms in intron 2 and 5 of cathepsin C are associated with Haim-Munk syndrome. Eur J Dent 8, 79-84.

Banda, Y., Kvale, M.N., Hoffmann, T.J., Hesselson, S.E., Ranatunga, D., Tang, H., Sabatti, C., Croen, L.A., Dispensa, B.P., Henderson, M., et al. (2015). Characterizing Race/Ethnicity and Genetic Ancestry for 100,000 Subjects in the Genetic Epidemiology Research on Adult Health and Aging (GERA) Cohort. Genetics 200, 1285-1295.

Boutboul, D., Kuehn, H.S., Van de Wyngaert, Z., Niemela, J.E., Callebaut, I., Stoddard, J., Lenoir, C., Barlogis, V., Farnarier, C., Vely, F., et al. (2018). Dominant-negative IKZF1 mutations cause a T, B, and myeloid cell combined immunodeficiency. J Clin Invest 128, 3071-3087.

Brooke, G., Holbrook, J.D., Brown, M.H., and Barclay, A.N. (2004). Human lymphocytes interact directly with CD47 through a novel member of the signal regulatory protein (SIRP) family. J Immunol 173, 2562-2570.

Cai, X.Y., Zheng, X.D., Fang, L., Zhou, F.S., Sheng, Y.J., Wu, Y.Y., Yu, C.X., Zhu, J., and Xiao, F.L. (2017). A variant on chromosome 2p13.3 is associated with atopic dermatitis in Chinese Han population. Gene 628, 281-285.

Cappellano, G., Orilieri, E., Comi, C., Chiocchetti, A., Bocca, S., Boggio, E., Bernardone, I.S., Cometa, A., Clementi, R., Barizzone, N., et al. (2008). Variations of the perforin gene in patients with multiple sclerosis. Genes Immun 9, 438-444.

Chang, C.C., Chow, C.C., Tellier, L.C., Vattikuti, S., Purcell, S.M., and Lee, J.J. (2015). Second-generation PLINK: rising to the challenge of larger and richer datasets. Gigascience 4, 7.

Clementi, R., Emmi, L., Maccario, R., Liotta, F., Moretta, L., Danesino, C., and Arico, M. (2002). Adult onset and atypical presentation of hemophagocytic lymphohistiocytosis in siblings carrying PRF1 mutations. Blood 100, 2266-2267.

Delaneau, O., Marchini, J., and Zagury, J.F. (2011). A linear complexity phasing method for thousands of genomes. Nat Methods 9, 179-181.

Demenais, F., Margaritte-Jeannin, P., Barnes, K.C., Cookson, W.O.C., Altmüller, J., Ang, W., Barr, R.G., Beaty, T.H., Becker, A.B., Beilby, J., et al. (2018). Multiancestry association study identifies new asthma risk loci that colocalize with immune-cell enhancer marks. Nat Genet 50, 42-53.

Ellinghaus, D., Jostins, L., Spain, S.L., Cortes, A., Bethune, J., Han, B., Park, Y.R., Raychaudhuri, S., Pouget, J.G., Hübenthal, M., et al. (2016). Analysis of five chronic inflammatory diseases identifies 27 new associations and highlights disease-specific patterns at shared loci. Nat Genet 48, 510-518.

Feinberg, H., Rowntree, T.J., Tan, S.L., Drickamer, K., Weis, W.I., and Taylor, M.E. (2013). Common polymorphisms in human langerin change specificity for glycan ligands. J Biol Chem 288, 36762-36771.

Franke, A., McGovern, D.P., Barrett, J.C., Wang, K., Radford-Smith, G.L., Ahmad, T., Lees, C.W., Balschun, T., Lee, J., Roberts, R., et al. (2010). Genome-wide meta-analysis increases to 71 the number of confirmed Crohn's disease susceptibility loci. Nat Genet 42, 1118-1125.

Hallberg, P., Smedje, H., Eriksson, N., Kohnke, H., Daniilidou, M., Öhman, I., Yue, Q.Y., Cavalli, M., Wadelius, C., Magnusson, P.K.E., et al. (2019). Pandemrix-induced narcolepsy is associated with genes related to immunity and neuronal survival. EBioMedicine 40, 595-604.

Hallmayer, J., Faraco, J., Lin, L., Hesselson, S., Winkelmann, J., Kawashima, M., Mayer, G., Plazzi, G., Nevsimalova, S., Bourgin, P., et al. (2009). Narcolepsy is strongly associated with the T-cell receptor alpha locus. Nat Genet 41, 708-711.

Han, F., Faraco, J., Dong, X.S., Ollila, H.M., Lin, L., Li, J., An, P., Wang, S., Jiang, K.W., Gao, Z.C., et al. (2013). Genome wide analysis of narcolepsy in China implicates novel immune loci and reveals changes in association prior to versus after the 2009 H1N1 influenza pandemic. PLoS Genet 9, e1003880.

Hayat, S.M.G., Bianconi, V., Pirro, M., Jaafari, M.R., Hatamipour, M., and Sahebkar, A. (2020). CD47: role in the immune system and application to cancer therapy. Cell Oncol (Dordr) 43, 19-30.

Hirota, T., Takahashi, A., Kubo, M., Tsunoda, T., Tomita, K., Sakashita, M., Yamada, T., Fujieda, S., Tanaka, S., Doi, S., et al. (2012). Genome- wide association study identifies eight new susceptibility loci for atopic dermatitis in the Japanese population. Nat Genet 44, 1222-1226.

Howie, B.N., Donnelly, P., and Marchini, J. (2009). A flexible and accurate genotype imputation method for the next generation of genome-wide association studies. PLoS Genet 5, e1000529.

Jin, Y., Birlea, S.A., Fain, P.R., Ferrara, T.M., Ben, S., Riccardi, S.L., Cole, J.B., Gowan, K., Holland, P.J., Bennett, D.C., et al. (2012). Genome-wide association analyses identify 13 new susceptibility loci for generalized vitiligo. Nat Genet 44, 676-680.

Jordan, M.B., Hildeman, D., Kappler, J., and Marrack, P. (2004). An animal model of hemophagocytic lymphohistiocytosis (HLH): CD8+ T cells and interferon gamma are essential for the disorder. Blood 104, 735-743.

Juvodden, H.T., Viken, M.K., Nordstrand, S.E.H., Viste, R., Westlye, L.T., Thorsby, P.M., Lie, B.A., and Knudsen-Heier, S. (2020). HLA and sleep parameter associations in post-H1N1 narcolepsy type 1 patients and first-degree relatives. Sleep 43.

Kash, J.C., Xiao, Y., Davis, A.S., Walters, K.A., Chertow, D.S., Easterbrook, J.D., Dunfee, R.L., Sandouk, A., Jagger, B.W., Schwartzman, L.M., et al. (2014). Treatment with the reactive oxygen species scavenger EUK-207 reduces lung damage and increases survival during 1918 influenza virus infection in mice. Free Radic Biol Med 67, 235-247.

Kichaev, G., Bhatia, G., Loh, P.R., Gazal, S., Burch, K., Freund, M.K., Schoech, A., Pasaniuc, B., and Price, A.L. (2019). Leveraging Polygenic Functional Enrichment to Improve GWAS Power. Am J Hum Genet 104, 65-75.

Lappalainen, T., Sammeth, M., Friedländer, M.R., t Hoen, P.A., Monlong, J., Rivas, M.A., Gonzàlez- Porta, M., Kurbatova, N., Griebel, T., Ferreira, P.G., et al. (2013). Transcriptome and genome sequencing uncovers functional variation in humans. Nature 501, 506-511.

Liu, Q., Zhou, Y.H., and Yang, Z.Q. (2016). The cytokine storm of severe influenza and development of immunomodulatory therapy. Cell Mol Immunol 13, 3-10.

Lowin, B., Beermann, F., Schmidt, A., and Tschopp, J. (1994). A null mutation in the perforin gene impairs cytolytic T lymphocyte- and natural killer cell-mediated cytotoxicity. Proc Natl Acad Sci U S A 91, 11571-11575.

Manku, H., Langefeld, C.D., Guerra, S.G., Malik, T.H., Alarcon- Riquelme, M., Anaya, J.M., Bae, S.C., Boackle, S.A., Brown, E.E., Criswell, L.A., et al. (2013). Trans-ancestral studies fine map the SLE- susceptibility locus TNFSF4. PLoS Genet 9, e1003554.

Marchini, J., Howie, B., Myers, S., McVean, G., and Donnelly, P. (2007). A new multipoint method for genome-wide association studies by imputation of genotypes. Nat Genet 39, 906-913.

Martínez-Pomar, N., Lanio, N., Romo, N., Lopez-Botet, M., and Matamoros, N. (2013). Functional impact of A91V mutation of the PRF1 perforin gene. Hum Immunol 74, 14-17.

Myers, L.M., Tal, M.C., Torrez Dulgeroff, L.B., Carmody, A.B., Messer, R.J., Gulati, G., Yiu, Y.Y., Staron, M.M., Angel, C.L., Sinha, R., et al. (2019). A functional subset of CD8(+) T cells during chronic exhaustion is defined by SIRPα expression. Nat Commun 10, 794.

Ng, W.C., Londrigan, S.L., Nasr, N., Cunningham, A.L., Turville, S., Brooks, A.G., and Reading, P.C. (2016). The C-type Lectin Langerin Functions as a Receptor for Attachment and Infectious Entry of Influenza A Virus. J Virol 90, 206-221.

Noack, B., Gorgens, H., Hempel, U., Fanghanel, J., Hoffmann, T., Ziegler, A., and Schackert, H.K. (2008). Cathepsin C gene variants in aggressive periodontitis. J Dent Res 87, 958-963.

O'Connor, D., Png, E., Khor, C.C., Snape, M.D., Hill, A.V.S., van der Klis, F., Hoggart, C., Levin, M., Hibberd, M.L., and Pollard, A.J. (2019). Common Genetic Variations Associated with the Persistence of Immunity following Childhood Immunization. Cell Rep 27, 3241-3253.e3244.

Onengut-Gumuscu, S., Chen, W.M., Burren, O., Cooper, N.J., Quinlan, A.R., Mychaleckyj, J.C., Farber, E., Bonnie, J.K., Szpak, M., Schofield, E., et al. (2015). Fine mapping of type 1 diabetes susceptibility loci and evidence for colocalization of causal variants with lymphoid gene enhancers. Nat Genet 47, 381-386.

Orilieri, E., Cappellano, G., Clementi, R., Cometa, A., Ferretti, M., Cerutti, E., Cadario, F., Martinetti, M., Larizza, D., Calcaterra, V., et al. (2008). Variations of the perforin gene in patients with type 1 diabetes. Diabetes 57, 1078-1083.

Perisic Nanut, M., Sabotic, J., Jewett, A., and Kos, J. (2014). Cysteine cathepsins as regulators of the cytotoxicity of NK and T cells. Front Immunol 5, 616.

Petukhova, L., Duvic, M., Hordinsky, M., Norris, D., Price, V., Shimomura, Y., Kim, H., Singh, P., Lee, A., Chen, W.V., et al. (2010). Genome-wide association study in alopecia areata implicates both innate and adaptive immunity. Nature 466, 113-117.

Pruim, R.J., Welch, R.P., Sanna, S., Teslovich, T.M., Chines, P.S., Gliedt, T.P., Boehnke, M., Abecasis, G.R., and Willer, C.J. (2010). LocusZoom: regional visualization of genome-wide association scan results. Bioinformatics 26, 2336-2337.

Pulit-Penaloza, J.A., Esser, E.S., Vassilieva, E.V., Lee, J.W., Taherbhai, M.T., Pollack, B.P., Prausnitz, M.R., Compans, R.W., and Skountzou, I. (2014). A protective role of murine langerin+ cells in immune responses to cutaneous vaccination with microneedle patches. Sci Rep 4, 6094.

Sinha, S., Borcherding, N., Renavikar, P.S., Crawford, M.P., Tsalikian, E., Tansey, M., Shivapour, E.T., Bittner, F., Kamholz, J., Olalde, H., et al. (2018). An autoimmune disease risk SNP, rs2281808, in SIRPG is associated with reduced expression of SIRPγ and heightened effector state in human CD8 T-cells. Sci Rep 8, 15440.

Slots, J. (2004). Update on human cytomegalovirus in destructive periodontal disease. Oral Microbiol Immunol 19, 217-223.

Slots, J., and Contreras, A. (2000). Herpesviruses: a unifying causative factor in periodontitis? Oral Microbiol Immunol 15, 277-280.

Sun, Y.X., Tang, L., Wang, P., Abbas, M.N., Tian, J.W., Zhu, B.J., and Liu, C.L. (2018). Cathepsin L- like protease can regulate the process of metamorphosis and fat body dissociation in Antheraea pernyi. Dev Comp Immunol 78, 114-123.

Terrell, C.E., and Jordan, M.B. (2013). Perforin deficiency impairs a critical immunoregulatory loop involving murine CD8(+) T cells and dendritic cells. Blood 121, 5184-5191.

Toyoda, H., Miyagawa, T., Koike, A., Kanbayashi, T., Imanishi, A., Sagawa, Y., Kotorii, N., Kotorii, T., Hashizume, Y., Ogi, K., et al. (2015). A polymorphism in CCR1/CCR3 is associated with narcolepsy. Brain Behav Immun 49, 148-155.

Vicente, C.T., Revez, J.A., and Ferreira, M.A.R. (2017). Lessons from ten years of genome-wide association studies of asthma. Clin Transl Immunology 6, e165.

Voskoboinik, I., Sutton, V.R., Ciccone, A., House, C.M., Chia, J., Darcy, P.K., Yagita, H., and Trapani, J.A. (2007). Perforin activity and immune homeostasis: the common A91V polymorphism in perforin results in both presynaptic and postsynaptic defects in function. Blood 110, 1184-1190.

Westra, H.J., Martínez-Bonet, M., Onengut-Gumuscu, S., Lee, A., Luo, Y., Teslovich, N., Worthington, J., Martin, J., Huizinga, T., Klareskog, L., et al. (2018). Fine-mapping and functional studies highlight potential causal variants for rheumatoid arthritis and type 1 diabetes. Nat Genet 50, 1366-1374.

Yang, C., Li, Z., Kang, W., Tian, Y., Yan, Y., and Chen, W. (2016). TET1 and TET3 are essential in induction of Th2-type immunity partly through regulation of IL-4/13A expression in zebrafish model. Gene 591, 201-208.

Zhu, G.H., Zhang, L.P., Li, Z.G., Wei, A., Yang, Y., Tian, Y., Ma, H.H., Wang, D., Zhao, X.X., Zhao, Y.Z., et al. (2020). Associations between PRF1 Ala91Val polymorphism and risk of hemophagocytic lymphohistiocytosis: a meta-analysis based on 1366 subjects. World J Pediatr 16, 598-606.
