# Supplementary material for: Narcolepsy risk loci outline role of T cell autoimmunity and infectious triggers in narcolepsy
Source: Nat Commun. 2023 May 15;14:2709. doi: 10.1038/s41467-023-36120-z (PMC10185546; doi:10.1038/s41467-023-36120-z)
Supplement: Supplementary file 3 — Description of Additional Supplementary Files [file 41467_2023_36120_MOESM3_ESM.pdf]

Description of Additional Supplementary Files

File Name: Supplementary Data 1
Description: **Known effects of narcolepsy variants.** We summarize earlier literature for the variants and genetic loci.

File Name: Supplementary Data 2
Description: **Tissue specific stratified LD score regression.** We show results from associations by each tissue type and functional annotation (Name) and the respective enrichment coefficient (Coefficient), coefficient standard error (coefficient se) and P-value. Raw two-sided P-values from weighted linear regression are reported.

File Name: Supplementary Data 3
Description: **Association of rs1154155 with T cell receptor repertoire and chain usage.** We provide association statistics between rs1154155 and gene chain usage for the following parameters: Distance between variant and gene, R-value, P-value, P-value - log10, Adjusted r2, Gradient, Chain, Empiric p-value, SNP rank, and SNPs per gene. Raw and adjusted two sided P-values from linear regression are reported.

File Name: Supplementary Data 4
Description: **Association of rs7458379 with T cell repertoire and chain usage.** We provide association statistics between rs7458379 and gene chain usage for the following parameters: Distance between variant and gene, R-value, P-value, P-value - log10, Adjusted r2, Gradient, Chain, Empiric p-value, SNP rank, and SNPs per gene. Raw and adjusted two sided P-values from linear regression are reported.

File Name: Supplementary Data 5
Description: **Association of rs1154155 on T Cell receptor alpha chain expression in T cell subsets.** We provide association statistics for rs1154155 and the chain usage per immune cell subset. Effect size (BETA) and standard error (SE) and P-values are computed for the alternative allele (A1). Raw two sided P-values from linear regression are reported.

File Name: Supplementary Data 6
Description: **Association of rs1483979 on T Cell receptor alpha chain expression in T cell subsets.** We provide association statistics for rs1483979 and the chain usage per immune cell subset. Effect size (BETA) and standard error (SE) and P-values are computed for the alternative allele (A1). Raw two sided P-values from linear regression are reported.
