# Supplementary material for: Narcolepsy risk loci outline role of T cell autoimmunity and infectious triggers in narcolepsy
Source: Nat Commun. 2023 May 15;14:2709. doi: 10.1038/s41467-023-36120-z (PMC10185546; doi:10.1038/s41467-023-36120-z)
Supplement: Supplementary file 10 — Reporting Summary [file 41467_2023_36120_MOESM10_ESM.pdf]

nature portfolio

Corresponding author(s): Emmanuel Mignot

Last updated by author(s): Oct 19, 2022

Reporting Summary

Nature Portfolio wishes to improve the reproducibility of the work that we publish. This form provides structure for consistency and transparency in reporting. For further information on Nature Portfolio policies, see our Editorial Policies and the Editorial Policy Checklist.

Statistics

For all statistical analyses, confirm that the following items are present in the figure legend, table legend, main text, or Methods section.

n/a	Confirmed	
☐	☒	The exact sample size (*n*) for each experimental group/condition, given as a discrete number and unit of measurement
☐	☒	A statement on whether measurements were taken from distinct samples or whether the same sample was measured repeatedly
☐	☒	The statistical test(s) used AND whether they are one- or two-sided<br>*Only common tests should be described solely by name; describe more complex techniques in the Methods section.*
☐	☒	A description of all covariates tested
☐	☒	A description of any assumptions or corrections, such as tests of normality and adjustment for multiple comparisons
☐	☒	A full description of the statistical parameters including central tendency (e.g. means) or other basic estimates (e.g. regression coefficient) AND variation (e.g. standard deviation) or associated estimates of uncertainty (e.g. confidence intervals)
☐	☒	For null hypothesis testing, the test statistic (e.g. *F*, *t*, *r*) with confidence intervals, effect sizes, degrees of freedom and *P* value noted<br>*Give P values as exact values whenever suitable.*
☒	☐	For Bayesian analysis, information on the choice of priors and Markov chain Monte Carlo settings
☐	☒	For hierarchical and complex designs, identification of the appropriate level for tests and full reporting of outcomes
☐	☒	Estimates of effect sizes (e.g. Cohen's *d*, Pearson's *r*), indicating how they were calculated

Our web collection on statistics for biologists contains articles on many of the points above.

Software and code

Policy information about availability of computer code

Data collection	No software was used.
Data analysis	Affypipe, Affymetrix genotyping console, Genome Studio. Samples, PLINK 1.9, SHAPEIT v2.2, IMPUTE2 v2.3.2, SNPTEST v.2.5.2, METAv1.7, GCTA, coloc package in R version 3.4.2 (2017-09-28), QQman, FUMA, R glm package, PRSice, HLA*IMP:02 as implemented in Affymetrix HLA, HIBAG package in R version 3.1.2 (2014-10-31), R version 3.2.2, Encode from Garfield, LCSC package, TopHat, StringTie, Kallisto, cuffcompare, Matrix eQTL, qvalue package (https://github.com/StoreyLab/qvalue), modified version of HTSeq56 (which allows reads to map to a sequence of more than one V/D/J/C-gene), fastqc

For manuscripts utilizing custom algorithms or software that are central to the research but not yet described in published literature, software must be made available to editors and reviewers. We strongly encourage code deposition in a community repository (e.g. GitHub). See the Nature Portfolio guidelines for submitting code & software for further information.

Data

Policy information about availability of data

All manuscripts must include a data availability statement. This statement should provide the following information, where applicable:

- Accession codes, unique identifiers, or web links for publicly available datasets
- A description of any restrictions on data availability
- For clinical datasets or third party data, please ensure that the statement adheres to our policy

All data generated and analysed in this study has been deposited in the sleep disorder knowledge portal database (sumstats) and the supplementary tables and supplementary data of this manuscript.

Field-specific reporting

Please select the one below that is the best fit for your research. If you are not sure, read the appropriate sections before making your selection.

☒ Life sciences ☐ Behavioural & social sciences ☐ Ecological, evolutionary & environmental sciences

For a reference copy of the document with all sections, see nature.com/documents/nr-reporting-summary-flat.pdf

Life sciences study design

All studies must disclose on these points even when the disclosure is negative.

Sample size	6,073 cases and 84,556 controls were used in this study, thus representing the largest GWAS conducted for this disease so far. The number of cases was chosen upon maximal number we were able to collect from collaborators. The number of controls was chosen in order to achieve maximum matching power for principal component analysis (patient vs. controls, 1:10 or more)..
Data exclusions	No data has been excluded, except for quality control failures, see methods for details.
Replication	The replication in post-Pandemrix® cases is detailed and discussed carefully in the text and shown in Table 1, where we show that 7 out of 13 loci replicate nominally, with two loci being even genome-wide significant. Second, the PRS replicates highly significantly in the post-Pandemrix® cases.
Randomization	Samples were segregated by ethnicity and genotyping platform and experimental groups defined based on principal component analysis. Further, immunisation with Pandemrix® and comorbidity with other autoimmune diseases was used to segregate study populations for specific analyses, as outlined in the main text.
Blinding	Blinding was not applicable in this study and it is a requirement to define the phenotype of cases vs. controls in the analysis.

Reporting for specific materials, systems and methods

We require information from authors about some types of materials, experimental systems and methods used in many studies. Here, indicate whether each material, system or method listed is relevant to your study. If you are not sure if a list item applies to your research, read the appropriate section before selecting a response.

Materials & experimental systems

n/a	Involved in the study	
☒	☐	Antibodies
☒	☐	Eukaryotic cell lines
☒	☐	Palaeontology and archaeology
☒	☐	Animals and other organisms
☐	☒	Human research participants
☒	☐	Clinical data
☒	☐	Dual use research of concern

Methods

n/a	Involved in the study	
☒	☐	ChIP-seq
☒	☐	Flow cytometry
☒	☐	MRI-based neuroimaging

Human research participants

Policy information about studies involving human research participants

Population characteristics	An ethnically diverse population was used in this study, including subjects of European (mixed European and nation specific cohorts from Ireland, Sweden, Norway, Finland), East Asian (mixed East Asian, including nation specific cohorts from China and Japan) and African American decent. The studied population included individuals both with and without immunization with Pandemrix®. Information on treatment and disease-comorbidities was recorded and controlled for or taken into considerations for sub-group specific analyses. Relevant population characteristics are outlined in Supplementary Table 1.
Recruitment	Patients were recruited in sleep clinics at multiple locations in the US, Europe and East Asia and diagnosis was confirmed using standard diagnostic guidelines. Collaborating physicians, institutions and clinics were selected based on previous publications and a structured recruiting process, whereby institutions were provided with the aims and objectives of this study and the opportunity to contribute. We do not assume any self-selection bias or other biases in the recruitment that are likely to impact results.
Ethics oversight	Stanford University IRB review board has reviewed and approved this study.

Note that full information on the approval of the study protocol must also be provided in the manuscript.

March 2021
